# Linker histone H1.2 inhibits HSV-1-induced IFN response via cGAS

**DOI:** 10.1128/mbio.03881-25

**Published:** 2026-04-13

**Authors:** Sirui Li, Chenglong Li, Fengyi Zhou, Yihua Zhang, Manman Li, Bingying Xie, Lulu Ning, Xinguang Lin, Baoyu Zhao, Xiaowu Hong, Dapeng Yan

**Affiliations:** 1 Department of Immunology, School of Basic Medical Sciences, Fudan University 58305, Shanghai, China; 2 Ningbo Research Institute, Fudan University, Ningbo, China; 3 Shanghai Institute of Infectious Disease and Biosecurity & Shanghai Public Health Clinical Center, Fudan University 12478, Shanghai, China; 4 Key Laboratory of Medical Molecular Virology, Department of Medical Microbiology and Parasitology, School of Basic Medical Sciences, Fudan University 58305, Shanghai, China; Ulm University Medical Center, Ulm, Baden-Württemberg, Germany

**Keywords:** linker histone H1.2, cGAS, HSV, IFN response

## Abstract

Linker histone H1.2 (H1.2), as a member of the histone H1 family involved in many cellular physiological regulatory functions, plays a vital role in maintaining nucleosome and chromatin stability. Cyclic GMP-AMP synthase (cGAS) is a critical cytoplasmic DNA sensor that activates the downstream STING pathway by synthesizing 2′3′-cGAMP, which, in turn, triggers IFN-I response following double-stranded DNA virus infection. This study demonstrates that histone H1.2 is an essential negative regulator of cGAS, inhibiting antiviral immunity during HSV-1 infection. Mechanistically, H1.2 affects the activity of the Lys240 site of cGAS to affect the combination of cGAS with chromatin and promotes the degradation of cGAS in the nucleus by recruiting TRIM28, finally suppressing its IFN response. Moreover, HSV-1 infection downregulates H1.2 expression by reducing the mRNA levels of its transcription factor Sp1, thus allowing cGAS release to activate type I interferon signaling. Additionally, plicamycin, a selective inhibitor of Sp1, can reduce H1.2 expression and enhance antiviral immunity in mice against HSV-1 infection. These findings elucidate the function and regulatory mechanisms of the Sp1-H1.2-cGAS axis in innate immunity and propose new targets and strategies for antiviral drug development.

**IMPORTANCE:**

Previous studies on histone H1.2 mainly focused on its function of DNA damage repair and chromatin stability. However, our research found the new function and mechanism of H1.2 in anti-infection immune regulation and confirmed H1.2 as an important negative regulatory molecule responsible for inhibition of cGAS, the important sensor in pathogenic recognition. In the nucleus, H1.2 maintained the inactive state of cGAS by promoting its combination to chromatin and recruiting TRIM28 to degrade the inactive cGAS. We revealed the mechanism of host cells regulating antiviral immunity through the Sp1-H1.2-cGAS axis and found plicamycin could be used as a potential anti-infective drug. These data may offer important reference value for innate immune research.

## INTRODUCTION

Innate immunity is the first line of defense against pathogen invasion. It starts with the recognition of pathogen-associated molecular patterns (PAMPs) by pattern recognition receptors (PRRs) (1). Key PRRs include toll-like receptors (TLRs), NOD-like receptors (NLRs), RIG-I-like receptors (RLRs), and cytoplasmic DNA sensors (2, 3), which can identify various microbial components and directly activate specific immune responses.

DNA sensors are a critical, yet sometimes overlooked, component of the PRR family, detecting pathogen and self-damaged DNAs. Among DNA sensors, cyclic GMP-AMP synthase (cGAS) is crucial in numerous cellular biochemical and physiological processes, consisting of an unstructured N-terminal with positive charge and a C-terminal containing NTase and Mab21 region. CGAS specifically recognizes double-stranded DNA (dsDNA) from pathogens and self-damaging cells, as well as complementary DNA (cDNA) synthesized by retroviruses such as HIV (4–6). Upon DNA recognition, cGAS catalyzes the synthesis of 2′3′-cyclic GMP-AMP (2′3′-cGAMP) (7) from GTP and ATP, which then translocates to the endoplasmic reticulum to activate the stimulator of interferon genes (STING) pathway. Activated STING recruits TANK-binding kinase 1 (TBK1) (8), which phosphorylates and promotes the dimerization of interferon regulatory factor 3 (IRF3). The dimerized IRF3 then translocates into the nucleus, initiating the transcription of downstream interferons (IFNs) and inflammatory factors (9). Additionally, STING can activate IκB kinase (IKK), which phosphorylates IκBα, leading to the release of nuclear factor kappa B (NF-κB) and promoting the expression of proinflammatory cytokines, such as tumor necrosis factor (TNF) and interleukin-6 (IL-6), to mediate immune and inflammatory responses (10).

In addition to its role in the antiviral response, the cGAS-STING pathway is implicated in various diseases and cellular physiological and biochemical processes. Tumor cells often undergo chromosome segregation errors during mitosis, forming micronuclei that can rupture and release dsDNA into the cytosol (11). Mitochondria are central participants in innate immunity. Cells experiencing metabolic abnormalities, such as oxidative stress or mitochondrial dysfunction, can also release mitochondrial DNA (mtDNA) into the cytosol (12), which can be recognized by cytoplasmic cGAS, activating downstream IFN pathways and inflammatory responses (13, 14) that can be protective or pathologic. High cGAS and STING expression are associated with increased T-cell infiltration, elevated PD-L1 and PD-1 levels, improved immunotherapy response, and prolonged survival. In tumor cells represented by breast cancer cells, the expression of cGAS-STING is downregulated for immune escape (15). Moreover, the cGAS-STING axis recognizes DNA damage and is involved in autophagy and apoptosis (16, 17). As cGAS is also present in the nucleus and binds with a variety of nuclear substructures such as nucleosomes, the double-stranded breaks (DSBs), centromeres, and line DNA repeats (18–21), its translocation between nucleus and cytoplasm is dynamically regulated (22). The combination of dimer-cGAS with nucleosome inhibits nuclear cGAS activation by inhibiting its enzyme activity and reducing its DNA-sensing ability (19, 23–25). Furthermore, cGAS undergoes various posttranslational modifications, including phosphorylation, ubiquitination, glutamylation, ribosylation, and SUMOylation (26–30), which influence its stability, localization, and function. Given that the dynamic circulation of cGAS between the cytoplasm and nucleus affects numerous cellular processes, understanding the regulation of cGAS within the nucleus is crucial for comprehending its role in immune network modulation.

Histone H1 is a ubiquitous chromatin protein that binds DNA to nucleosomes to stabilize the chromatin complex and is involved in transcription regulation and DNA damage repair (31–33). H1.2 is the most widely distributed histone H1 subtype, exhibiting the highest expression levels and strong species conservation, functioning as a housekeeping protein. Mechanistically, H1.2 interacts with the chromatin remodeling complex BRG1 in an ATP-dependent manner to stabilize chromosomes, leading to chromatin densification and gene suppression (34). Additionally, H1.2 initiates PARP1-dependent DNA repair through poly ADP-ribosylation and maintains genomic integrity by accurately repairing DNA double-strand breaks via homologous recombination (35). H1.2 phosphorylation by DNA-PK on Thr146 reduces its interaction with p53, resulting in p53 activation and ultimately inhibiting tumor cell growth (36). Additionally, H1.2 depletion triggers an interferon response in cancer cells by activating heterochromatic repeats (37). Recent studies have indicated that H1.2 can also contribute to antiviral immunity. For example, H1.2 regulates IFN-β and inhibits influenza virus replication by interacting with IRF3 (38). H1.2 can also affect the nuclear translocation of IRF3 to inhibit the replication of encephalomyocarditis virus (EMCV) in cells (39). Current studies on the antiviral role of H1.2 primarily focus on its effects on RNA viruses; however, its impact on DNA viruses remains unclear.

This study demonstrated that H1.2 interacts with cGAS in the nucleus, inhibiting the cGAS-mediated IFN pathway. Knocking out of H1.2 increased the antiviral immune response of mice to HSV-1 infection. Several residues are crucial for cGAS binding to nucleosomes (40). Our further investigation revealed that H1.2 restricted cGAS to nucleosomes in a Lys240-dependent manner, preventing its translocation into the cytoplasm. Additionally, H1.2 recruited TRIM28 to degrade inactive cGAS. Moreover, HSV-1 infection downregulated H1.2 expression through inhibiting the transcription factor Sp1, and the Sp1 inhibitor plicamycin decreased H1.2 expression and enhanced antiviral immunity, indicating that plicamycin might be a potential antiviral drug candidate.

## RESULTS

### H1.2 deficiency enhances antiviral immune response

**Fig 1:**
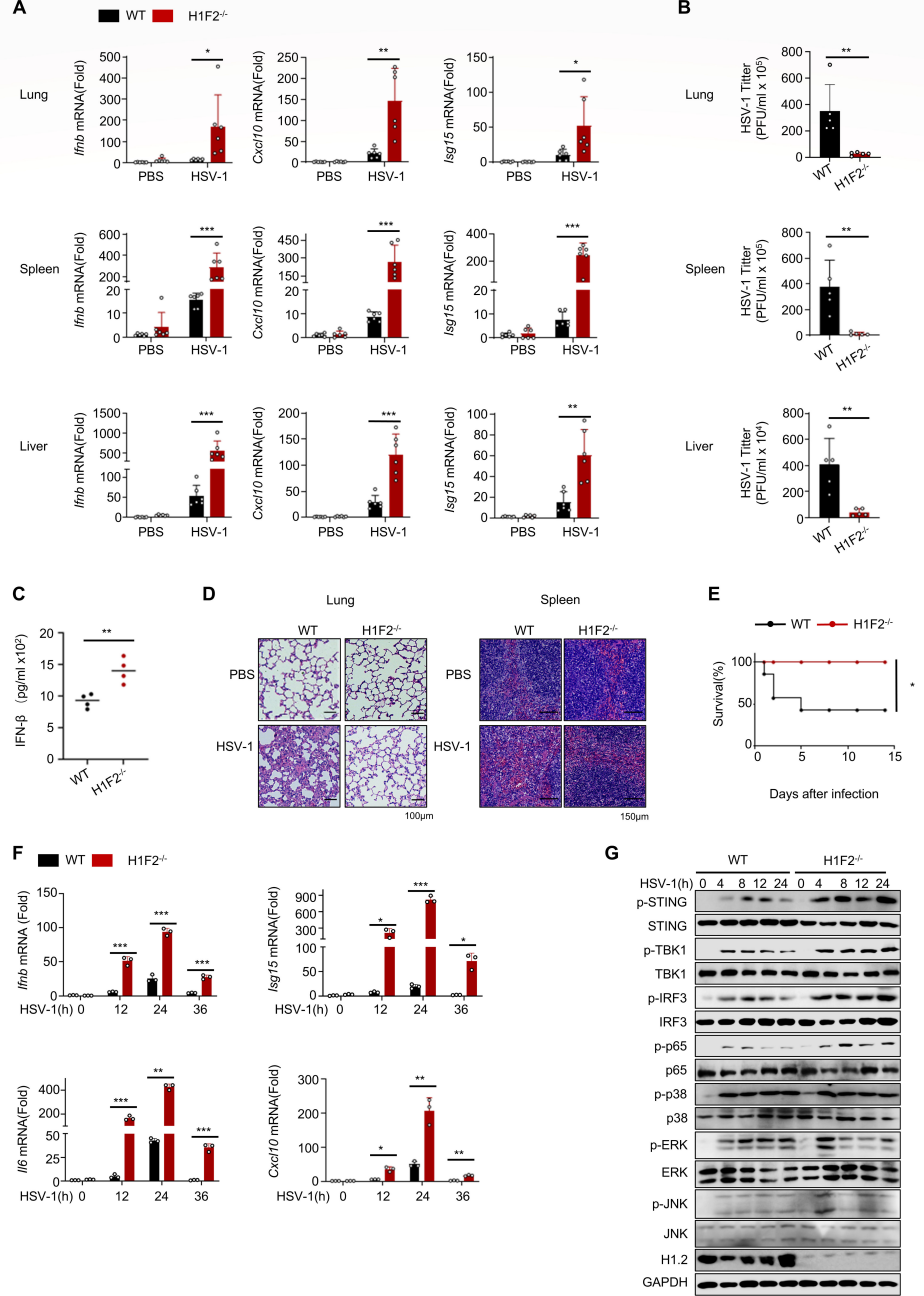
H1.2 deficiency enhances antiviral immune response. (**A**) *Ifnb, Isg15,* and *Cxcl10* mRNA levels (*n* = 6) in the spleens, livers, and lungs of WT or H1F2^−/−^ mice intraperitoneally injected with PBS or HSV-1 (2 × 10^8^ PFU per mouse) for 48 h. (**B**) HSV-1 titers (*n* = 5) of tissues of WT or H1F2^−/−^ mice treated as in panel **A**. (**C**) ELISA assay of IFN-β content in peripheral blood of WT or H1F2^−/−^ mice (*n* = 4) treated as in panel **A**. (**D**) Microscopy of hematoxylin- and eosin-stained lung and spleen sections as in panel **A**. (**E**) Survival of WT or H1F2^−/−^ mice (*n* = 8) infected intraperitoneally with a high dose of HSV-1 (2 × 10^10^ PFU per mouse) and monitored for 15 days. (**F**) *Ifnb, Isg15, Il6,* and *Cxcl10* mRNA levels in peritoneal macrophages of WT or H1F2^−/−^ mice treated with HSV-1 for the indicated times (*n* = 3). (**G**) Immunoblot analysis of lysates of macrophages from WT or H1F2^−/−^ mice treated with HSV-1. Data are means ± SD. *P < 0.05, **P < 0.01, ***P < 0.001 (two-tailed unpaired Student’s *t*-test) in panel **A, B, and C**, or Kaplan–Meier analysis in panel **E**.

To investigate whether H1.2 is involved in DNA virus infection, we generated *H1f2* knockout (H1F2^−/−^) mice (Fig. S1A) and infected them and their wild-type (WT) counterparts with HSV-1 (2 × 10^8^ PFU per mouse). We evaluated the expression of *Ifnb, Cxcl10*, *Isg15,* and *Il6* as well as viral titers in different tissues. After HSV-1 infection, the expression of these cytokines in the lung, spleen, and liver of H1F2^−/−^ mice was substantially increased, while viral titers were markedly lower compared to the control group (Fig. 1A and B; Fig. S1B). Similarly, ELISA analysis revealed that the peripheral blood of H1F2^−/−^ mice contained higher levels of IFN-β than that of WT mice (Fig. 1C). Consistent with these findings, H1F2^−/−^ mice exhibited less structural disruption in the lung and spleen than WT mice (Fig. 1D). Additionally, we injected WT and H1F2^−/−^ mice with a high dose of HSV-1 (2 × 10^10^ PFU per mouse) to assess their survival rate and found that H1F2^−/−^ mice had a higher survival rate (Fig. 1E). We stimulated peritoneal macrophages from WT and H1F2^−/−^ mice with HSV-1 to further explore the immune response at the cellular level and observed that the expression of *Ifnb*, *Isg15*, *Cxcl10*, *Tnf,* and *Il6* was substantially elevated in H1F2^−/−^ peritoneal macrophages (Fig. 1F; Fig. S1C). Consistently, H1.2 deletion substantially enhanced the phosphorylation of STING, TBK1, IRF3, and p65 (Fig. 1G), indicating that H1.2 may regulate the immune response through the cGAS-STING pathway. We treated WT and H1F2^−/−^ peritoneal macrophages with herring testis DNA (HT-DNA) to confirm these results and observed similar outcomes (Fig. S1D and E). These findings indicate that H1.2 is a critical negative regulator in the antiviral immune response to DNA virus infection.

### H1.2 negatively regulates IFN-β signaling

**Fig 2:**
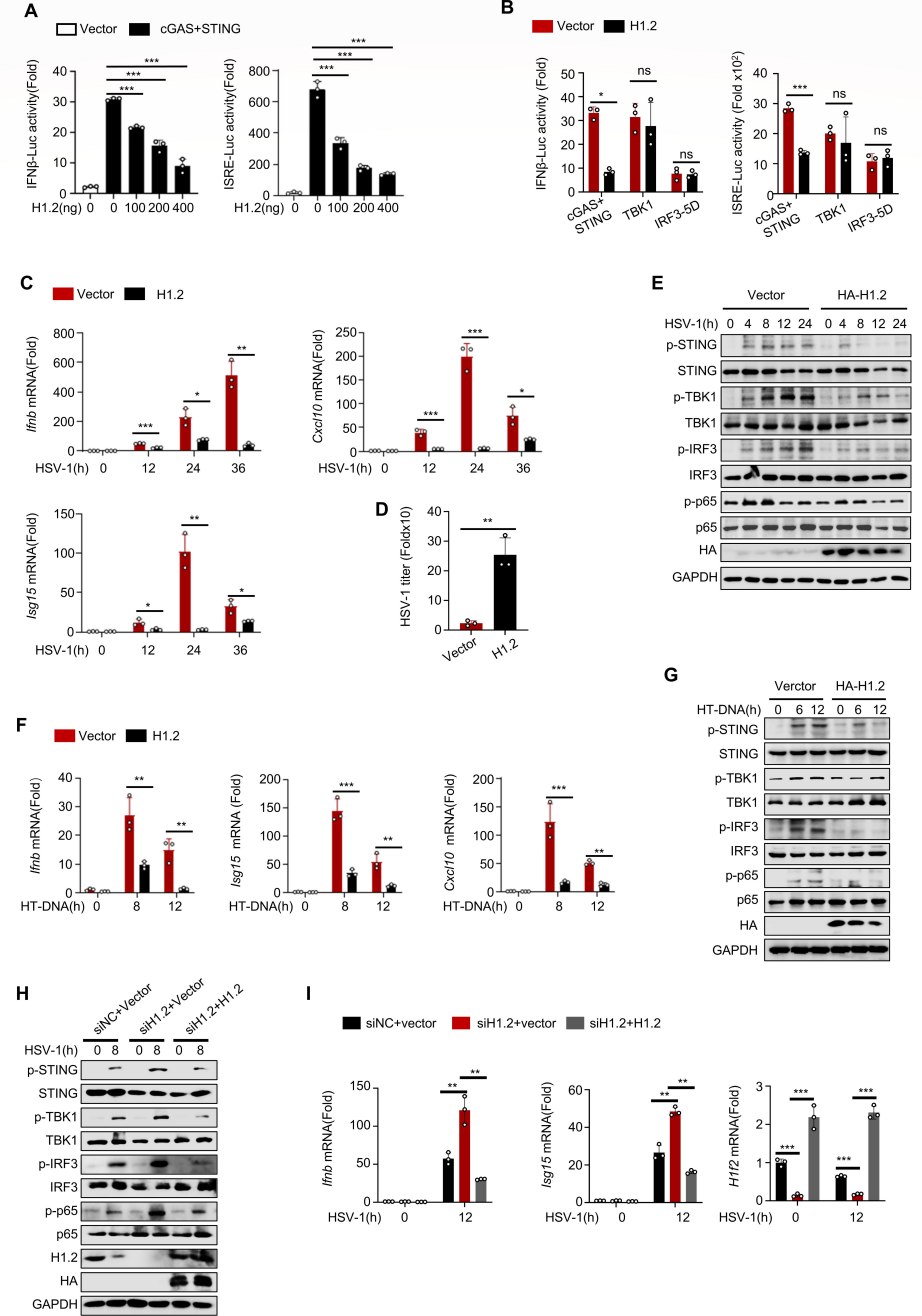
H1.2 negatively regulates IFN-β signaling. (**A**) Luciferase assay of IFN-β and ISRE of HEK293T cells transfected with cGAS, STING, IFN-Luc, TK, and different doses of H1.2, HA-tagged vector was used to ensure that the total amount of transfected plasmids was the same. (**B**) Luciferase assay of IFN-β and ISRE of HEK293T cells transfected with the indicated plasmids, HA-tagged vector was used to ensure that the total amount of transfected plasmids was the same. (**C**) *Ifnb, Isg15,* and *Cxcl10* mRNA levels in control- or H1.2-overexpressing L929s infected with HSV-1 for the indicated time (*n* = 3). (**D**) HSV-1 titers in control- or H1.2-overexpressing L929s infected with HSV-1 for 72 h. (**E**) Immunoblot analysis of lysates of L929s overexpressing control vector or H1.2 treated with HSV-1 for the indicated time. (**F**) Immunoblot analysis of lysates of L929s overexpressing control vector or H1.2 treated with HT-DNA. (**G**) *Ifnb, Isg15,* and *Cxcl10* mRNA levels in control- or H1.2-overexpressing L929s treated with HT-DNA for the indicated time as in panel **F**. (**H**) Immunoblot analysis of lysates of L929 cells transfected with specific siRNA and plasmids, and infected for the indicated time. (**I**) *Ifnb, Isg15,* and *H1f2* mRNA levels in L929 cells transfected with specific siRNA and plasmids and then stimulated with HSV-1 for the indicated time. Data are means ± SD. *P < 0.05, **P < 0.01, ***P < 0.001 (two-tailed unpaired Student’s *t*-test).

The cGAS-STING signaling pathway is crucial for intracellular innate immunity against DNA viruses. We conducted a dual-luciferase assay by overexpressing cGAS, STING, and H1.2 in HEK293T cells to determine whether H1.2 affects HSV-1 infection via the cGAS-STING pathway. Our results revealed that H1.2 inhibited IFN-β and interferon-stimulated response element (ISRE) activations in a dose-dependent manner (Fig. 2A). Using the same approach, we observed that H1.2 overexpression inhibited the activity of luciferase reporters for signaling protein-induced IFN-β and ISRE activation, except for TBK1 and IRF3-5D (Fig. 2B), indicating that H1.2 inhibits the activation of signaling proteins upstream of TBK1. Next, we investigated whether H1.2 overexpression substantially affected IFN-β signaling. In L929 mouse fibroblast cells, H1.2 overexpression substantially decreased the HSV-1-induced expression of *Ifnb*, *Isg15*, *Cxcl10*, *Tnf,* and *Il6* (Fig. 2C; Fig. S2A). Consistent with this finding, the HSV-1 titer was increased in H1.2-overexpressing L929 cells (Fig. 2D). We further examined the phosphorylation of STING, TBK1, IRF3, and p65 after HSV-1 infection. As expected, H1.2 overexpression in L929 cells reduced their phosphorylation compared to the control group (Fig. 2E). Similarly, H1.2 exhibited an inhibitory effect in mouse MEF cells (Fig. S2B and C). We then treated L929 cells with the HT-DNA and found that H1.2 also inhibited HT-DNA-induced IFN-β signaling activation (Fig. 2F and G; Fig. S2D). We transfected L929 cells with a negative control small interfering RNA (siRNA) or H1.2-specific siRNA to further confirm the function of H1.2 (Fig. S2E). Knocking down H1.2 in L929 cells could improve the transcription activity of IFNβ upon HSV-1 stimulation, which increased with the increase in knocking down level (Fig. S2F). We transfected L929 cells with either control siRNA (siNC) or H1.2-specific siRNA, followed by rescue with empty vector or H1.2-expressing plasmid, and assessed activation of the IFN pathway post-HSV-1 infection. Results demonstrated that H1.2 knockdown ‌upregulated phosphorylation of STING, TBK1, IRF3, and P65 (Fig. 2H)‌ and ‌enhanced production of inflammatory factors‌ (e.g., Ifnb, Isg15, Cxcl10). These effects were suppressed upon H1.2 rescue, ‌demonstrating H1.2 as an inhibitory molecule of the cGAS-STING pathway‌ (Fig. 2I; Fig. S2G). Altogether, these results indicate that H1.2 inhibits IFN-β signaling and the antiviral immune response induced by DNA viruses.

### H1.2 interacts with cGAS in the nucleus

**Fig 3:**
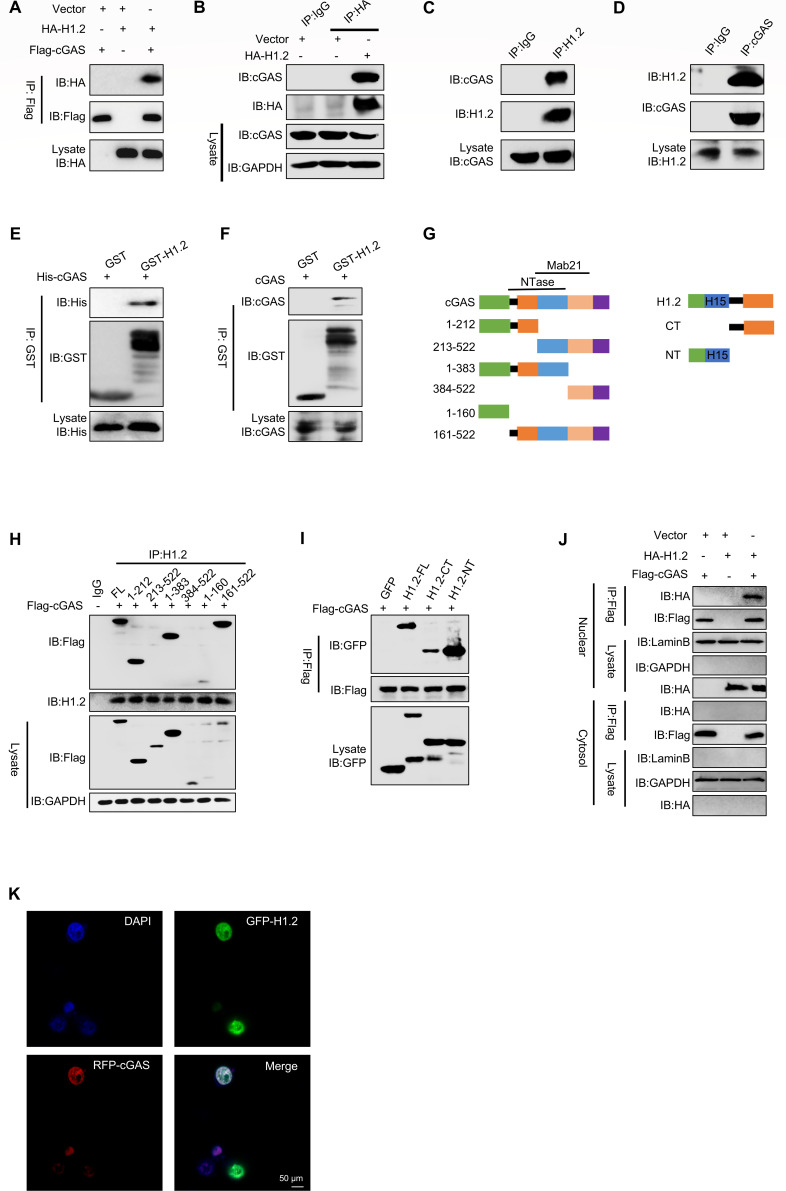
H1.2 interacts with cGAS in the nucleus. (**A**) Co-immunoprecipitation analysis in HEK293T cells transfected with the indicated plasmids. (**B**) Co-immunoprecipitation analysis in L929 cells transfected with the indicated plasmids. (**C and D**) Immunoblot of lysates of L929 cells. (**E**) GST pull-down assay of His–cGAS with GST-4T1 or GST-H1.2. (**F**) GST pull-down assay of GST-H1.2 with cGAS from the cytoplasm of L929 cells. (**G**) Deletion mutant construction of H1.2 and cGAS. (**H**) Immunoprecipitation assay of H1.2 and deletion mutants of cGAS. (**I**) Immunoprecipitation assay of cGAS and deletion mutants of H1.2. (**J**) Immunocoprecipitation analysis was carried out on the nuclear and cytoplasmic components of HEK293T cells transfected with specific plasmids. (**K**) Immunofluorescence of HeLa cells expressing the indicated plasmids.

After verifying that H1.2 affects cGAS-mediated interferon response, we investigated whether H1.2 regulates innate immunity through cGAS. A coimmunoprecipitation (Co-IP)-based experiment demonstrated that H1.2 interacted with cGAS (Fig. 3A and B). To eliminate the influence of DNA bridge on the binding of H1.2 and cGAS, we used benzonase to degrade the nucleic acid chain in cells, and the results showed that H1.2 interacted with cGAS (Fig. S3B). The interaction between endogenous H1.2 and cGAS was further examined in L929 cells (Fig. 3C and D). We expressed and purified GST-H1.2 and His-cGAS in *Escherichia coli* and performed an *in vitro* GST precipitation assay, which indicated that H1.2 directly bound with purified cGAS (Fig. 3E) and endogenous cGAS (Fig. 3F). Next, we constructed a series of truncation mutants for H1.2 and cGAS to identify which of their domains are involved in their interaction (Fig. 3G). Both the N-terminal and C-terminal of H1.2 can bind to cGAS, but the N-terminal is the main binding region. And the 161-212 region of cGAS is also crucial for their interaction (Fig. 3H and I). We analyzed the binding surface between H1.2 and cGAS by protein-protein docking. The results showed that there were many highly confident sites in the 161–212 region of cGAS that could directly interact with H1.2 by hydrogen bonding, which potentially proved the direct binding between H1.2 and cGAS (Fig. S3D). To demonstrate that the interaction between H1.2 and cGAS is not dependent on a DNA bridge, we constructed the H1.2 DNA-binding-deficient mutant, H1.2-ΔH15, and performed co-immunoprecipitation in 293T cells. The results showed that H1.2 lacking the H15 domain still binds to cGAS, albeit with reduced affinity. This indicates that the binding between H1.2 and cGAS is independent of nuclear DNA presence and likely involves multiple domains and binding sites (Fig. S3C). In addition, we overexpressed cGAS in HEK293T cells, and the combination with H1.2 was obtained by mass spectrometry (Fig. S3E). Since H1.2 interacts with chromatin in the nucleus, we next explored the specific location of the H1.2 and cGAS interaction. We separated the cytoplasmic and nuclear components of HEK293T cells transfected with the indicated plasmids and conducted co-immunoprecipitation experiments in both fractions to detect the binding location. The results indicated that these two proteins interact in the nucleus but not in the cytoplasm (Fig. 3J). Immunofluorescence experiments revealed that both H1.2 and cGAS were localized within the nucleus, suggesting that their interaction likely occurred in the nuclear compartment. (Fig. 3K). Thus, H1.2 directly interacts with cGAS in the nucleus.

### H1.2 inhibits cGAS activation

**Fig 4:**
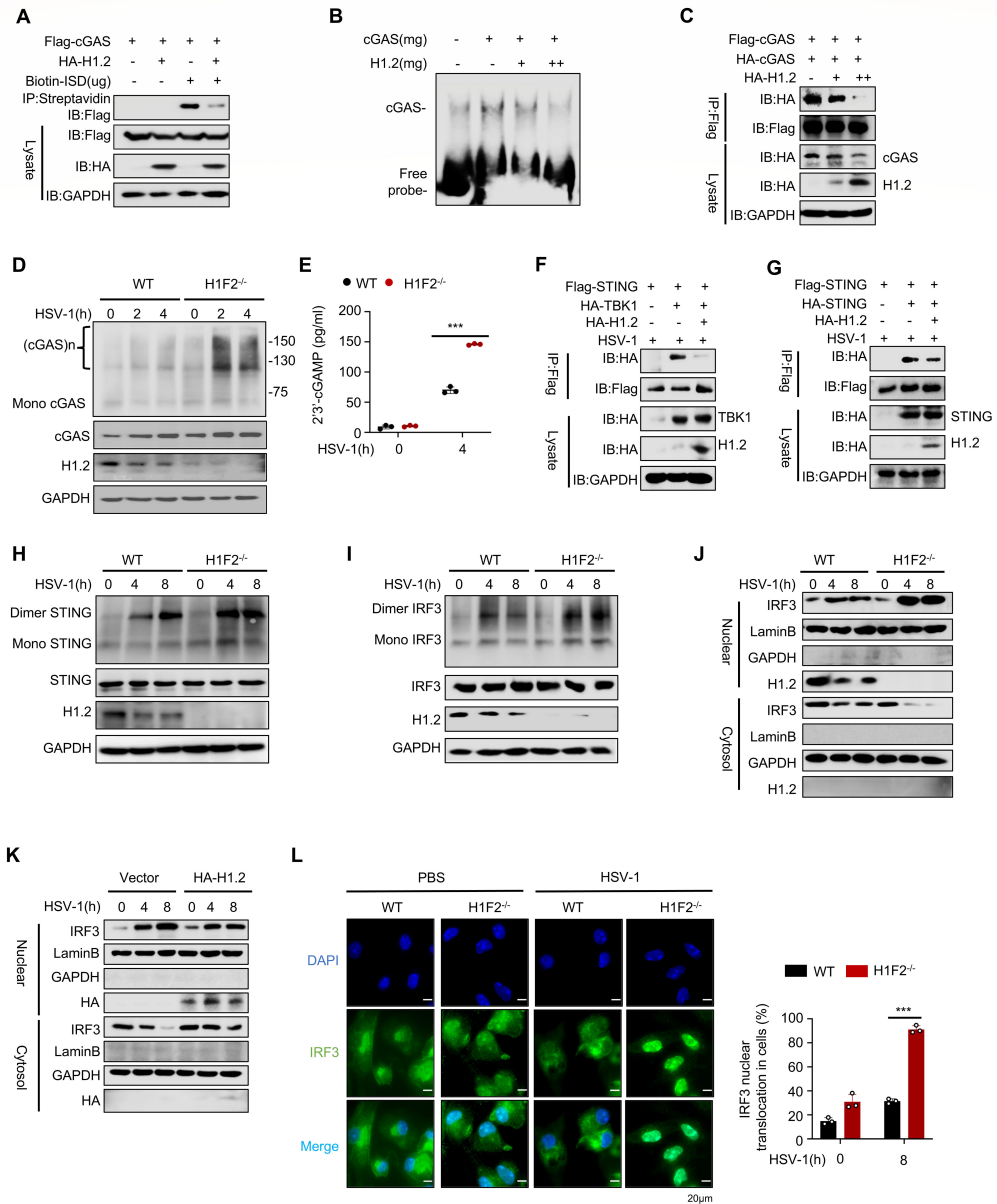
H1.2 inhibits cGAS activation. (**A**) The binding of cGAS and ISD is affected by H1.2 in HEK293T cells. (**B**) EMSA analysis of cGAS with dsDNA affected by H1.2. (**C**) Immunocoprecipitation of two-tagged cGAS in HEK293T cells co-expressing control vector or H1.2. (**D**) Native PAGE analysis of the oligomerization of cGAS. (**E**) ELISA assay of cGAMP of WT or knockout mice PMs. (**F and G**) Coimmunoprecipitation assay of binding of STING and TBK1 (**F**), STING and STING (**G**) in HEK293Ts transfected with the indicated plasmids under HSV-1 infection. (**H and I**) Native PAGE analysis of dimerization of STING (**H**) and IRF3 (**I**). (**J**) Immunoblot analysis of nuclear and cytoplasmic fractions of WT or H1F2^−/−^ peritoneal macrophages treated with HSV-1. (**K**) Immunoblot analysis of IRF3 nuclear localization of L929 cells overexpressing vector or H1.2 and infected with HSV-1. (**L**) Immunofluorescence microscopy and quantitative analysis of peritoneal macrophages from WT or H1F2^−/−^ mice treated with PBS or HSV-1 for 8h. Data are means ± SD. *P < 0.05, ***P < 0.001 (two-tailed unpaired Student’s *t*-test).

We stimulated peritoneal macrophages from H1F2^−/−^ mice and WT controls with the second messenger 2′,3′-cGAMP to explore the specific mechanism by which H1.2 regulates the interferon pathway through the cGAS-STING axis. The results indicated that direct STING stimulation with 2′,3′-cGAMP did not enhance the activity of the IFN-β pathway in the absence of H1.2 (Fig. S4A), indicating that the inhibitory effect of H1.2 occurs upstream of STING. The recognition of dsDNA by cGAS leads to conformational changes and cGAMP production, which subsequently activate STING and trigger IFN-β signaling transduction (6, 7). We performed a pull-down assay using biotin-labeled dsDNA with streptavidin to determine whether H1.2 affects the binding of cGAS with dsDNA. We found that H1.2 substantially inhibited the amount of cGAS pulled down by dsDNA (Fig. 4A). Similar results were obtained from the *in vitro* EMSA experiment (Fig. 4B). Since cGAS forms dimers upon dsDNA binding, we next investigated whether H1.2 influences the formation of cGAS dimers. The results indicated that H1.2 overexpression substantially decreased the interaction between Flag-tagged and HA-tagged cGASs (Fig. 4C). In contrast, knocking out H1.2 increased cGAS oligomerization (Fig. 4D) and promoted the production of cGAMP in infected cells (Fig. 4E; Fig. S4B), indicating that H1.2 could inhibit the DNA-binding activity of cGAS. As active cGAS could enhance the interaction between STING, TBK1, and IRF3, we examined the effects of H1.2 on these interactions. We found that under HSV-1 infection, H1.2 significantly inhibited cGAS-STING or STING-TBK1 interactions (Fig. 4F; Fig. S4C). We further assessed the effect of H1.2 on the dimerization of STING and IRF3 and found that H1.2 overexpression inhibited the dimerization of STING and IRF3 (Fig. 4G; Fig. S4D and E), while H1.2 knockout substantially increased their dimerization (Fig. 4H and I). The IRF3 translocation from the cytoplasm to the nucleus is critical for IFN-β production. We found that H1.2 knockout increased IRF3 translocation from the cytoplasm to the nucleus (Fig. 4J), while H1.2 overexpression decreased this nuclear localization (Fig. 4K; Fig. S4F). Additionally, we assessed IRF3 nuclear localization in mice peritoneal macrophage cells using immunofluorescence confocal microscopy, and the results were consistent with the western blot experiments (Fig. 4L). These data indicate that H1.2 inhibits cGAS-STING signaling activation by preventing cGAS binding to dsDNA, thus inhibiting the translocation of IRF3 from the cytoplasm to the nucleus.

### H1.2 ties cGAS to chromatin

**Fig 5:**
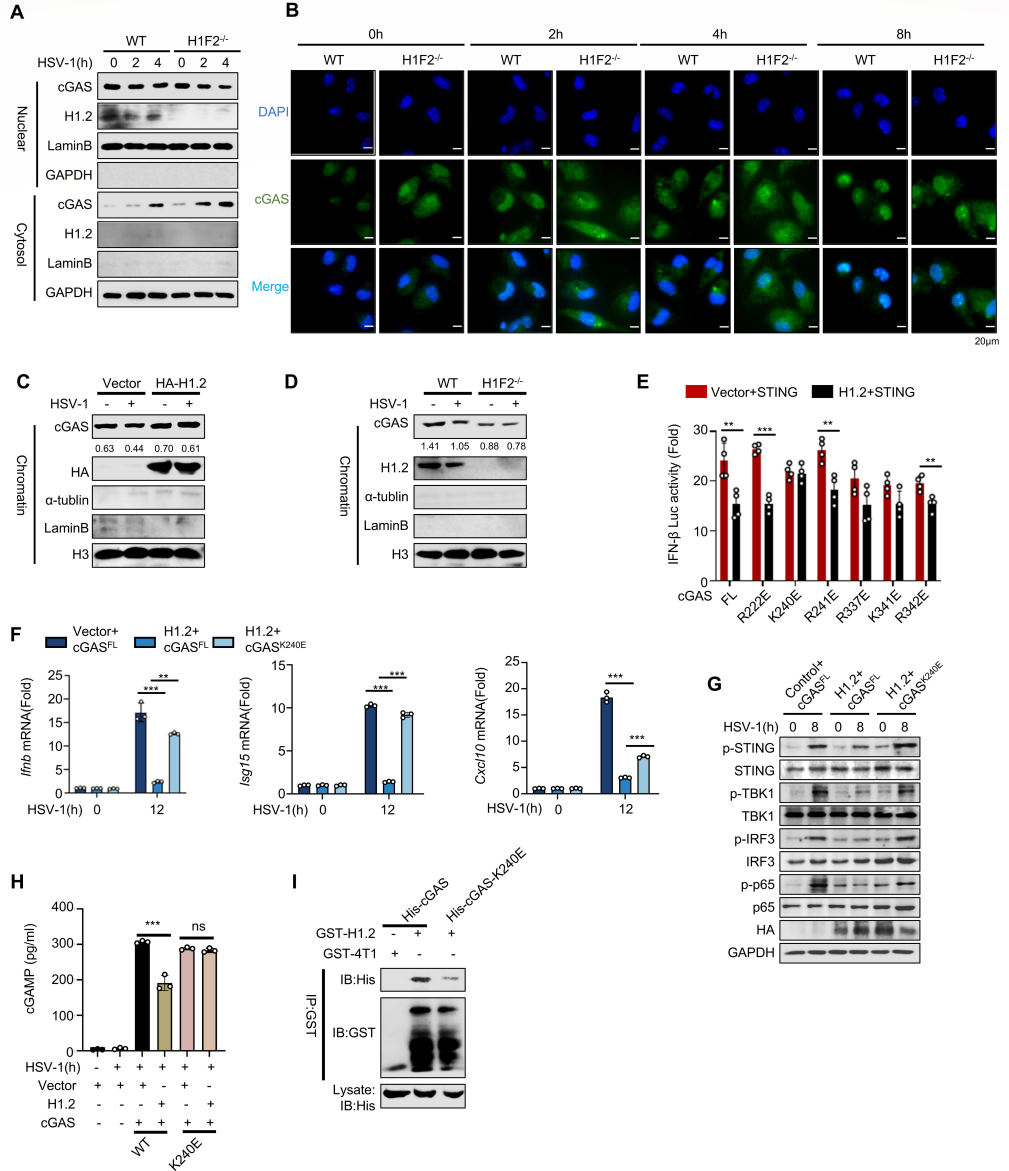
H1.2 ties cGAS to chromatin. (**A**) Immunoblot analysis of nuclear and cytoplasmic fractions of peritoneal macrophages from WT or H1F2^−/−^ mice treated with HSV-1 for the indicated times. (**B**) Immunofluorescence microscopy analysis of peritoneal macrophages from WT or H1F2^−/−^ mice treated with HSV-1 for the indicated hours. (**C and D**) Chromatin extraction analysis of L929 cells overexpressing control or H1.2 (**C**) or peritoneal macrophages from WT or H1F2^−/−^ mice (**D**) infected with HSV-1 for the indicated times. (**E**) Luciferase analysis of HEK293T cells transfected with Flag-STING, HA-H1.2, and Flag-cGAS-mutants. (**F**) *Ifnb*, *Isg15,* and *Cxcl10* mRNA levels of GAS^−/−^ MEFs transfected with vector, H1.2, cGAS-WT, or cGAS-K240E, and infected with HSV-1 for the indicated times (*n* = 3). (**G**) Immunoblot analysis of the phosphorylation of STING, TBK1, IRF3, and p65 of cGAS^−/−^ MEFs transfected with the indicated plasmids and infected with HSV-1 for the indicated times. (**H**) CGAMP ELISA analysis of cGAS^−/−^ MEF cell transfected with the indicated plasmids and infected with HSV-1 for 4 hours. (**I**) GST pull-down assay of GST-H1.2 and His-cGAS or His-cGAS-K240E. Data are means ± SD. *P < 0.05, **P < 0.01, ***P < 0.001 (two-tailed unpaired Student’s *t*-test).

Studies have demonstrated that cGAS has a strong affinity for nucleosomes in the nucleus (41), which buries its DNA-binding site and reduces its ability to recognize nuclear dsDNA and its dimerization and activation (11, 19, 25). Since H1.2 interacts with cGAS in the nucleus, we speculated that H1.2 might restrict cGAS to nucleosomes, preventing its translocation to the cytoplasm and subsequent signaling activation. Therefore, we examined the effect of H1.2 on the translocation of cGAS to test this hypothesis. Since HSV-1 can trigger the release of cGAS from chromatin to the nucleus or cytoplasm (42), we isolated and detected the components of the WT or knockout peritoneal macrophage. We observed that compared to wild-type mice, H1.2-deficient mice exhibited a ‌significant increase‌ in cytoplasmic cGAS accumulation and a ‌decrease‌ in nuclear cGAS accumulation in peritoneal macrophages after HSV-1 infection. Subsequent isolation of chromatin and nuclear soluble fractions revealed that the reduction of nuclear cGAS in H1.2^−/−^ PMS cells was ‌primarily attributed to diminished chromatin-bound cGAS‌ (Fig. 5A; Fig. S5A). Our immunofluorescence experiment showed that the accumulation of cGAS in cytoplasm increased after virus infection, and this effect weakened in the later stage of infection. However, the deletion of H1.2 significantly enhanced the accumulation of cGAS in cytoplasm. This proved that H1.2 could tether cGAS to nucleosome, and this binding effect existed independently of the existence of HSV-1. However, the higher accumulation of cGAS in the cytoplasm caused by the deletion of H1.2 suggested that this binding potentially functions in the process of affecting the accumulation of cGAS in the cytoplasm and responding to pathogens (Fig. 5B; Fig. S5B). Next, we evaluated the binding ability of cGAS to nucleosomes using a chromatin extraction experiment and found that overexpressing H1.2 enhanced their binding, effectively trapping cGAS in the nucleus (Fig. 5C). Conversely, knocking out H1.2 decreased the abundance of cGAS binding to chromatin (Fig. 5D).

The Arg222, Lys240, Arg241, Arg337, Lys341, and Arg342 residues of cGAS are involved in its binding to nucleosomes (40). Therefore, we constructed mutants of all these residues to identify the specific binding residue regulated by H1.2 and found that the Lys240 mutation eliminated the inhibitory effect of H1.2 overexpression on cGAS-induced activation of IFN-β. For the R337E and R341E mutants, the addition of H1.2 could still slightly inhibit the IFN production (Fig. 5E). Additionally, we found that cGAS^K240E^ reversed the inhibitory effect of H1.2 on dimerization of cGAS compared to WT cGAS^FL^ (Fig. S5C). Here, we hypothesized that H1.2 could potentially maintain the stability of nucleosome as well as the tethered environment between K240 site of cGAS and nucleosome, thus playing an inhibitory role on cGAS. Consistently, H1.2-inhibited cytokine production was reversed by the cGAS^K240E^ mutation (Fig. 5F; Fig. S5D). H1.2 inhibited cGAS-induced phosphorylation of STING, TBK1, IRF3, and p65, while the cGAS^K240E^ mutation reversed this inhibitory effect (Fig. 5G). We overexpressed vector H1.2, cGAS, and cGAS mutants in cGAS knockout MEF cells and detected the content of cGAMP in the cells. Overexpression of H1.2 could inhibit the cGAMP production of cGAS-WT overexpressed MEFs but failed to further inhibit the cGAMP production of cGAS-K240E (Fig. 5H). Additionally, we performed GST pull-down assays to examine the binding between H1.2 and both wild-type cGAS and the cGAS-K240E mutant. Results demonstrated that ‌H1.2 retained binding capacity to cGAS-K240E‌, albeit with ‌significantly reduced affinity‌ compared to H1.2-cGAS-WT interaction. This implies that the K240E site has an important influence on the stability of the H1.2-cGAS complex. The nucleosome bridge is not absolutely necessary for the combination of the two molecules; however, the binding integrity critically influences H1.2-mediated suppression of cGAS activity (Fig. 5I; Fig. S5E). Together, these results demonstrate that H1.2 inhibited cGAS activation and antiviral immunity by affecting the activity of the Lys240 site of cGAS.

### H1.2 degrades inactive cGAS by recruiting TRIM28

**Fig 6:**
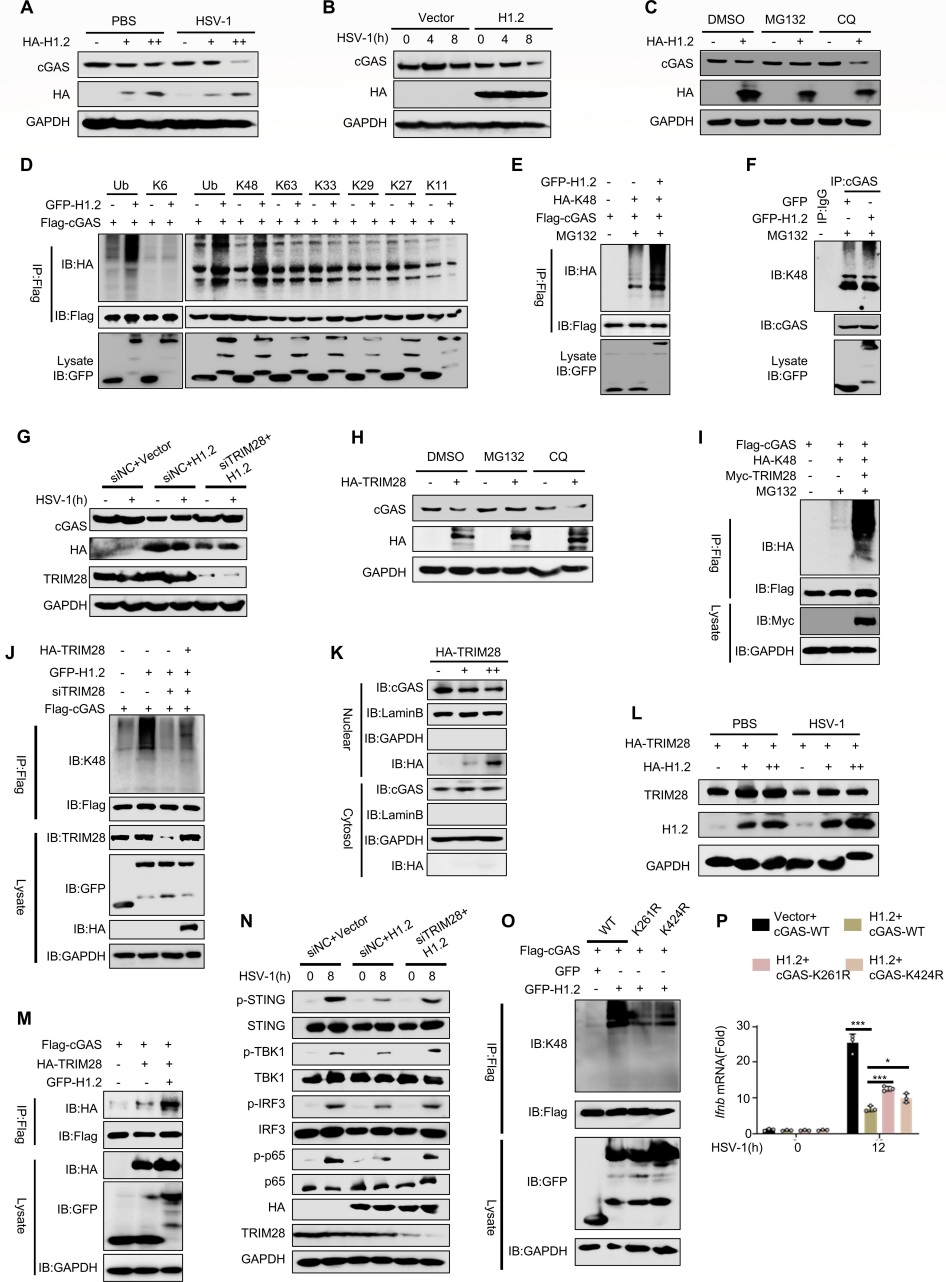
H1.2 promotes the degradation of inactive cGAS by recruiting TRIM28. (**A and B**) Immunoblot analysis of cGAS expression levels in L929 cells transfected with the indicated plasmids. (**C**) Immunoblot analysis of cGAS expression levels in L929 cells overexpressing vector or H1.2 and treated with DMSO or MG132 (10 μM for 6 h) or chloroquine (20 μM for 12 h). (**D–F**) Coimmunoprecipitation analysis of ubiquitination levels of cGAS in HEK293T (**D and E**) or L929 (**F**) cells overexpressing the indicated plasmids. (**G**) Results of endogenous cGAS in the immunoblotting experiment of L929 cells transfected with siRNA and DNA. (**H**) Immunoblot analysis of cGAS expression levels in vector or TRIM28 overexpressing L929 cells treated with MG132 (10 μM for 6 h) or CQ (20 μM for 12 h). (**I**) Immunoprecipitation analysis of K48-linked ubiquitination levels of cGAS affected by TRIM28 in HEK293T cells. (**J**) Immunoprecipitation analysis in L929 cells transfected with the indicated siRNA and plasmids. (**K**) Immunoblot analysis of expression levels of cGAS mediated by TRIM28 in nuclear and cytoplasmic fractions of HEK293T cells. (**L**) Immunoblot analysis of TRIM28 expression levels in L929 cells overexpressing the indicated plasmids. (**M**) Immunoblot analysis of the combination of cGAS and TRIM28 in HEK293T cells transfected with the indicated plasmids. (**N**) Immunoblot analysis of phosphorylation levels of STING, TBK1, IRF3, and p65 in L929s transfected with the indicated siRNA and plasmids. (**O**) Coimmunoprecipitation analysis of K48-ubiquitination levels of cGAS in L929s transfected with the indicated plasmids. (**P**) Analysis of mRNA levels of *Ifnb* in cGAS^−/−^ MEFs transfected with the indicated plasmids. Data are means ± SD. *P < 0.05, **P < 0.01, ***P < 0.001 (two-tailed unpaired Student’s *t*-test).

In our previous experiment, we strikingly found that overexpressing H1.2 inhibited cGAS expression in cell lysates (Fig. 3B). To confirm this phenomenon, we examined the effect of H1.2 on the expression of endogenous cGAS in L929 cells. The results indicated H1.2 overexpression inhibited cGAS in a dose-dependent manner, which existed independently of the stimulation of HSV-1 (Fig. 6A and B). Protein degradation occurs primarily through the ubiquitin-proteasome or the p62-mediated autophagy pathway (43, 44). We overexpressed H1.2 in L929 cells and treated them with the proteasome inhibitor MG132 or the autophagy inhibitor chloroquine (CQ). The results indicated that cGAS degradation by H1.2 was blocked by MG132 but not CQ treatment (Fig. 6C), indicating that H1.2 promotes cGAS degradation mainly via the proteasome pathway. We tested different ubiquitination forms of cGAS under H1.2 overexpression to determine the type of ubiquitination influenced by H1.2. The results indicated that overexpressing H1.2 substantially enhanced the total and K48-linked ubiquitination of cGAS (Fig. 6D through F).

As H1.2 is not a ubiquitin ligase, it must promote cGAS ubiquitination through another ubiquitin ligase. We overexpressed H1.2 in HEK293T cells, detected cGAS-binding proteins by mass spectrometry, and found that the ubiquitin ligase TRIM28 potentially interacted with cGAS in H1.2-overexpressing cells (Fig. S6A). TRIM28 is an E3 ubiquitin ligase involved in various cellular activities, including cell proliferation (45), gene expression (46), and DNA damage response (47, 48). Consistent with our hypothesis, we found that TRIM28 overexpression substantially inhibited cGAS accumulation, irrespective of HSV-1 infection (Fig. S6B). Additionally, we found that TRIM28 mediated cGAS degradation primarily through the proteasome pathway, but not the p62-mediated autophagy pathway (Fig. 6H; Fig. S6C). TRIM28 also promoted the K48-linked ubiquitination of cGAS (Fig. 6I; Fig. S6D and E), which were consistent with the results of the ubiquitination experiment *in vitro* (Fig. S6F). We constructed the siRNA of TRIM28 (Fig. S6G). To investigate whether the suppression of cGAS by H1.2 is associated with TRIM28, we knocked down TRIM28 and overexpressed H1.2 in L929 cells, followed by the detection of endogenous cGAS content in cell lysates. The results showed that H1.2 overexpression significantly inhibited cGAS accumulation. However, upon TRIM28 knockdown, the suppressive effect mediated by H1.2 was abolished, suggesting that H1.2 functions on cGAS degradation through TRIM28 (Fig. 6G). Besides, we found that upon TRIM28 knockdown, the enhancement effect of cGAS ubiquitination caused by overexpression of H1.2 was also inhibited, and this inhibitory effect was reversed when TRIM28 was replenished (Fig. 6J). We analyzed the nucleus and cytoplasmic components of HEK293T cells by immunoprecipitation to verify the location of degradation by TRIM28. The results indicated that cGAS degradation by TRIM28 mainly occurred in the nucleus (Fig. 6K). Moreover, we observed that H1.2 overexpression promoted TRIM28 accumulation, irrespective of HSV-1 infection (Fig. 6L). Overexpression of H1.2 enhanced the combination of TRIM28 and cGAS, while the absence of H1.2 weakened this combination. It is worth noting that the combination of TRIM28 and cGAS does not depend entirely on the existence of H1.2 (Fig. 6M; Fig. S6K). Furthermore, we found that upon TRIM28 knockdown, the suppressive effects of H1.2 overexpression on the cGAS-STING pathway and cytokines (e.g., *Ifnb, Isg15*) were reversed, which demonstrated that H1.2-mediated promotion of cGAS degradation via TRIM28 constitutes a critical mechanism through which H1.2 exerts its inhibitory effect on cGAS (Fig. 6N; Fig. S6H). To map the ubiquitination sites, we overexpressed TRIM28 and cGAS in L929 cells and analyzed cGAS by mass spectrometry. Two conserved lysine sites, Lys261 and Lys424, were the key sites enriched, which were homologous to human Lys275 and Lys439 sites (Fig. S6I and J). We verified the mutants of these two sites in L929 cells. The results demonstrated that both K261R and K424R attenuated the H1.2-induced ubiquitination of cGAS and partially rescued the suppressive effects on cytokines caused by H1.2 overexpression (Fig. 6O and P; Fig. S6L), Taken together, these results indicated that beyond promoting cGAS-chromatin binding to maintain its inactive state, H1.2 could recruit TRIM28 to facilitate ubiquitination degradation of cGAS via its Lys261 and Lys424 sites—this degradation mechanism constitutes a key component of H1.2-mediated suppression of cGAS.

### Plicamycin enhances antiviral immunity through the Sp1-H1.2-cGAS axis

**Fig 7:**
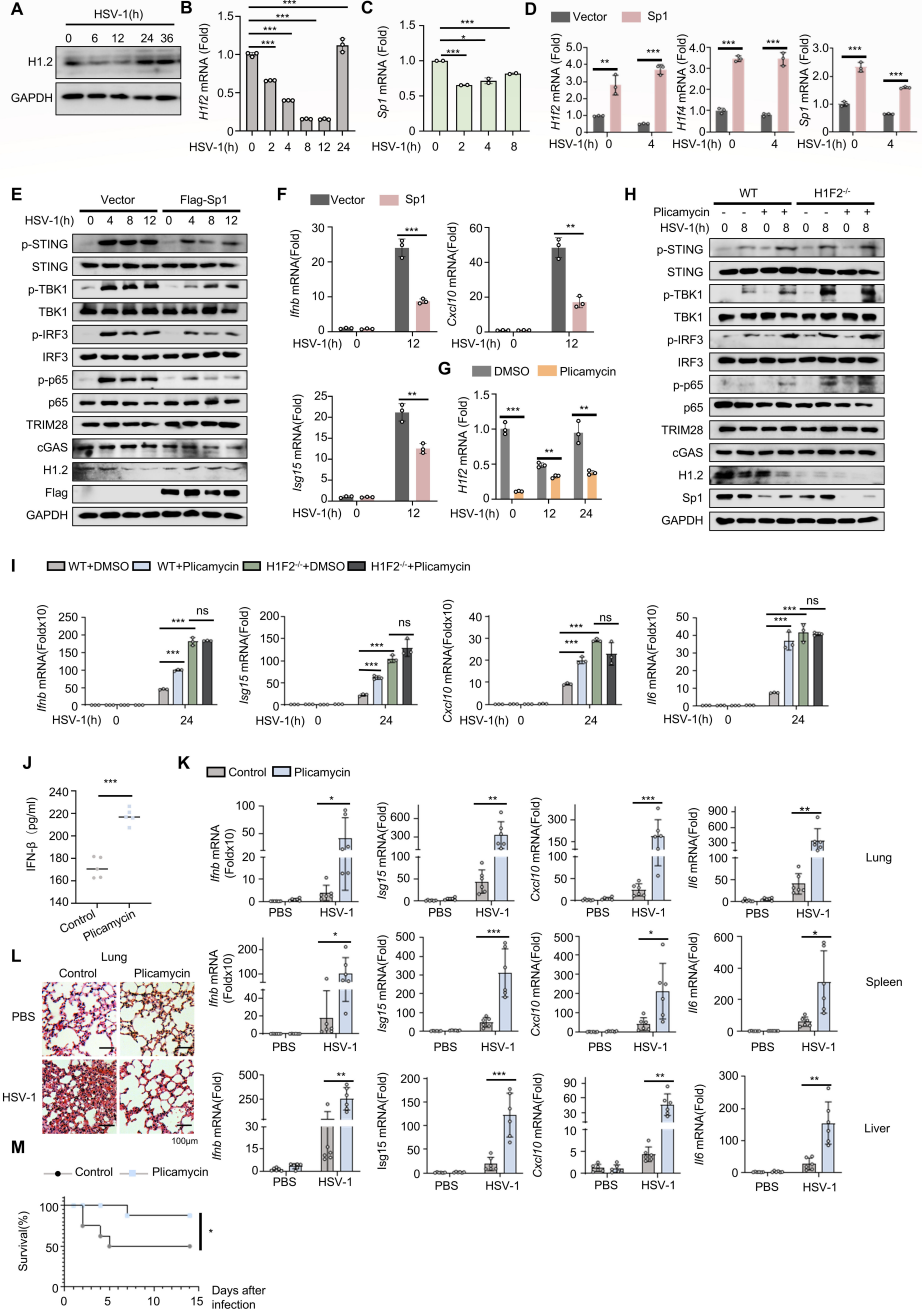
Plicamycin enhances antiviral immunity through the Sp1-H1.2-cGAS axis. (**A**) Immunoblot analysis of H1.2 expression of WT peritoneal macrophages infected with HSV-1 for the indicated times. (**B**) *H1f2* mRNA levels of WT peritoneal macrophages infected with HSV-1 for the indicated times. (**C**) Sp1 mRNA levels of L929 cells infected with HSV-1 for the indicated times. (**D**) *H1f2, H1f4,* and *Sp1* mRNA levels in vector or Sp1 overexpressing L929 cells infected with HSV-1 for the indicated times. (**E**) Immunoblot analysis of lysates of L929 cells transfected with vector or Sp1 and infected for the indicated times. (**F**) *Ifnb*, *Isg15,* and *Cxcl10* mRNA levels of L929 cells treated as in panel **E**. (**G**) H1f2 mRNA level of peritoneal macrophages treated with DMSO or plicamycin and infected with HSV-1. (**H**) Immunoblot analysis of lysate of peritoneal macrophages from WT or H1F2^−/−^ mice treated with or without plicamycin and infected with PBS or HSV-1 for the indicated times. (**I**) *Ifnb, Isg15, Cxcl10,* and *Il6* mRNA levels of peritoneal macrophages from WT or H1F2^−/−^ mice as in panel **H**. (**J**) ELISA analysis of the peripheral blood of WT mice (*n* = 5) intraperitoneally injected with HSV-1 (2 × 10^8^ PFU per mouse) for 24 h and then treated with PBS or plicamycin for 24 h. (**K**) *Ifnb*, *Isg15*, *Cxcl10,* and *Il6* mRNA levels in the lungs, spleens, and livers of WT mice as in panel **J**. (**L**) Microscopy of hematoxylin- and eosin-stained lung sections of WT mice (*n* = 6 per group) treated as in panel **J**.(**M**) Survival of WT mice (*n* = 8) infected intraperitoneally with a high dose of HSV-1 (2 × 10^10^ PFU per mouse) and treated with PBS or plicamycin 24 h after infection and monitored for 2 weeks. Data are means ± SD. *P < 0.05, **P < 0.01, ***P < 0.001 (two-tailed unpaired Student’s *t*-test), or Kaplan–Meier analysis in panel **M**.

To explore how H1.2 functions in host anti-infection immunity, we analyzed H1.2 expression in peritoneal macrophages following HSV-1 infection to investigate whether and how H1.2 is regulated in the antiviral innate immune response. We observed that H1.2 expression decreased shortly after HSV-1 infection and increased again at the late stage of infection (Fig. 7A and B). Similar results were obtained in HEK293T cells (Fig. S7A). This is consistent with our observation in Fig. 1G. We found that ‌H1.2 expression rebounded to basal levels‌ at 24 h post-infection, coinciding with ‌decreased phosphorylation of STING, TBK1, IRF3, and P65‌. We propose the hypothesis that downregulation of H1.2 post-infection may represent a host regulatory mechanism: Reduced H1.2 expression potentiates cGAS-mediated innate immune activation, thereby eliciting antiviral responses. Herpesviruses employ multiple host shutoff proteins (e.g., the virion host shutoff (VHS) protein and immediate-early regulatory protein ICP27 of HSV-1) to directly target messenger RNAs (mRNAs), leading to broad downregulation of host gene expression, constituting an essential process that reallocates cellular resources toward viral replication and evasion of host antiviral immune responses (49, 50). However, in the HT-DNA-stimulated PMS cell model, we similarly observed a trend of transient downregulation followed by upregulation in Sp1 and H1.2, indicating that the regulation does not originate from virus-host shutoff induced by HSV-1, but rather stems from an autonomous antiviral regulatory mechanism of the host cells (Fig. S7B). Based on these findings, we propose that upon pathogen DNA invasion, host cells actively downregulate H1.2 expression to facilitate cGAS release and accumulation, thereby triggering STING-mediated immune responses.

Sp1 is a transcription factor of H1.2 that binds to the H1.2 promoter and enhances its expression (51). GC-boxes and related motifs are frequently occurring DNA elements present in many promoters and enhancers. In contrast to other elements, it was generally thought that the transcription factor Sp1 is the only factor acting through these motifs (52). The replication-dependent histones are rich in CG box, including 16 genes for histone H2A, 22 genes for histone H2B, 14 genes for histone H3, 14 genes for histone H4, and 6 genes for histone H1 (53). The promoter structure of H3.3 is different from that of replication-dependent histone, and it lacks these enriched GC frames (54). We measured the Sp1 mRNA level in L929 cells to determine whether Sp1 is involved in antiviral immunity and found that its expression decreased substantially after viral infection (Fig. 7C). Furthermore, we found that overexpression of Sp1 led to an increase in the mRNA expression of two replication-dependent histones, H1.2 and H1.4, without affecting the expression of the replication-independent histone H3F3B, demonstrating that Sp1 promoted the transcription of H1.2, and this promoting effect was irrespective of an HSV-1 infection (Fig. 7D; Fig. S7C). We observed the same effect at the protein level, confirming that the promoting effect of Sp1 overexpression on H1.2 mRNA expression was ultimately reflected at the protein level (Fig. S7D). Consistently, overexpressing Sp1 inhibited the phosphorylation of STING, TBK1, IRF3, and p65 (Fig. 7E), as well as cytokine production (Fig. 7F; Fig. S7E).

Plicamycin is reported to be an Sp1 inhibitor that substantially inhibits the growth of cancer cells (55, 56). We found that plicamycin treatment inhibited H1.2 expression following HSV-1 infection (Fig. 7G). Additionally, plicamycin effectively enhanced the phosphorylation of STING, TBK1, IRF3, and p65, consistent with the results of Sp1 knockdown experiment (Fig. S7G and H), and the mRNA levels of cytokines, such as *Ifnb*, *Il6, Isg15*, and *Cxcl10* (Fig. S7F), and consistently inhibited the replication of HSV-1 (Fig. S7F). However, plicamycin did not further enhance the immune response when H1.2 was knocked out (Fig. 7H and I).

We next infected 6-week-old mice with HSV-1 to investigate the role of plicamycin in the antiviral immune response *in vivo*. We treated mice with or without plicamycin for 24 h before evaluating the immune response. The results indicated that plicamycin increased the IFN-β level in peripheral blood (Fig. 7J) and the expression of *Ifnb*, *Isg15*, *Cxcl10*, *Il6,* and *Tnf* in the lungs, spleens, and livers (Fig. 7I; Fig. S7I). Consistent with these results, we observed that plicamycin substantially inhibited the replication of HSV-1 in the lungs, spleens, and livers, as well as the viral titer in these tissues (Fig. S7I and M) and reduced inflammatory damage in the lungs and spleens (Fig. 7L; Fig. S7J). In addition, we assessed the effect of plicamycin on the survival rate of mice and found that the plicamycin-treated group had a higher survival rate (Fig. 7M). Additionally, we monitored body weight changes in control and drug-treated mice throughout the survival study. Two weeks post-treatment, serum was collected for ALT and AST measurements. Notably, while plicamycin-treated mice exhibited higher survival rates under high-titer HSV-1 challenge, they showed lower weight gain and higher serum ALT and AST levels compared to controls, especially ALT—though remaining within normal limits—potentially attributable to our dosing regimen. This ALT elevation represents a clinically relevant observation that could not be ignored despite the survival benefit (Fig. S7K and L).

Furthermore, to determine whether the suppression of cGAS-mediated IFN response is unique to H1.2 or common among histone H1 family members, we constructed plasmids expressing H1.0 to H1.5. Using IFN-β luciferase reporter assays in HEK293T cells, we demonstrated that ‌H1.2, H1.3, and H1.5 significantly inhibited IFN-β transcriptional activity‌, while H1.4 showed a mild suppressive effect. Notably, H1.2 exhibited the most pronounced suppression (Fig. S8A). Through co-immunoprecipitation assays in HEK293T cells co-expressing individual H1 proteins and cGAS, we confirmed that ‌H1.2, H1.3, and H1.5 physically interact with cGAS (Fig. S8B)‌. Additionally, we validated that these three isoforms (H1.2/H1.3/H1.5) promote cGAS ubiquitination, while H1.2 demonstrated the strongest effect (Fig. S8C).

These data reveal an Sp1-H1.2-IFN regulatory axis in host cells. Upon pathogen DNA invasion, host cells downregulate H1.2 expression through Sp1, thereby modulating the cGAS-mediated IFN pathway to initiate immune responses. Pharmacological inhibition of Sp1 accumulation by plicamycin enhances interferon production dependent on H1.2, demonstrating that the Sp1-H1.2 axis critically governs upstream innate immunity. Both Sp1 and H1.2 could represent potential targets for developing antiviral immunomodulatory agents. The regulatory effect of H1.2 on cGAS may be common among subtypes of the H1 family.

## DISCUSSION

The histone H1 family consists of linker histones with a tripartite structure. In eukaryotic cells, H1 histones are not part of the core components of the nucleosome; instead, they bind to linker DNA at the entry and exit sites of the nucleosome to stabilize the entire complex (57). Mammalian cells contain 11 different histone H1 subtypes. Among these, five are somatic variants specific to the S-phase of mammalian cells and are expressed in a replication-dependent manner (H1.1, H1.2, H1.3, H1.4, and H1.5) (58). Previous studies have established that histone H1 plays crucial roles in nucleosome stabilization, mRNA metabolism, protein-protein interactions, DNA damage repair, and ribosome function regulation (59). In recent years, studies have also found that members of the H1 family are involved in immune responses to RNA viruses (39, 60). So, does histone H1 also participate in the immune response to DNA viruses? Our research has found that several members of the H1 family (H1.2, H1.3, H1.4, and H1.5) inhibit the transcriptional activation of IFNβ, with histone H1.2 showing the most pronounced inhibitory effect. Furthermore, H1.2, H1.3, and H1.5 all demonstrate significant binding interactions with cGAS and can promote the ubiquitination level of cGAS, with H1.2 showing the strongest promoting effect (Fig. S8A through C). This suggests that the involvement of the H1 family in interferon responses through interaction with cGAS may be a common characteristic of some H1 family members—a characteristic that provides a theoretical basis for the role of histone H1 in regulating immune responses to DNA virus infections.

As a member of the histone H1 family, H1.2 is essential in stabilizing chromatin structure, repairing DNA damage, and regulating tumor immunity (35, 36, 59). Recent studies have demonstrated that H1.2 is involved in the immune response of RNA viruses and promotes IFN-β transcription, thereby inhibiting viral replication (38, 39). However, these studies have not deeply studied the infection regulation mechanism of H1.2, and few studies are available on the effect of H1.2 on DNA virus infection (61, 62). Our study revealed that H1.2 knockout mice exhibited increased IFN-β production, reduced viral titer in tissues, and decreased inflammatory damage from HSV-1 infection. Additionally, H1.2 overexpression inhibited the activation of the IFN-β pathway and reduced the phosphorylation of STING, TBK1, IRF3, and p65 in cells. Importantly, we discovered that while H1.2 did not interfere with the activation of STING by cGAMP, it bound directly to cGAS, impairing its DNA-binding activity and consequently blocking cGAMP formation.

The transcription factor Sp1 is critical in regulating gene transcription, cell proliferation, carcinogenesis, apoptosis, and viral infection (63). Notably, Sp1 acts as a positive transcription factor for replication-dependent histones, including H1.2 (51). Our research demonstrated that HSV-1 infection downregulates the transcription of Sp1 and H1.2. It is hypothesized that Sp1 positively regulates the expression of H1.2 to reduce the phosphorylation of STING, TBK1, IRF3, and p65 and cytokine production, inhibiting antiviral immunity. Thus, the Sp1-H1.2-cGAS axis is a key regulatory pathway in antiviral immunity. Previous studies have proposed that plicamycin inhibits the growth of various cancers by decreasing Sp1 expression (64). When we used plicamycin to reduce Sp1 expression, H1.2 expression was also substantially inhibited, activating the cGAS-STING pathway. Our animal experiments further demonstrated that plicamycin remarkably promoted cytokine production in mice, enhancing their antiviral immune response to HSV-1 and significantly improving the survival performance of mice infected with high-titer HSV-1. However, despite its significant antiviral effects, ‌the hepatotoxicity induced by plicamycin cannot be overlooked‌. Although currently unsuitable for clinical application, we propose that it provides ‌a valuable reference for developing therapeutic strategies against viral co-infections in other diseases‌. Additionally, we believe ‌Sp1 and H1.2 harbor potential as novel drug targets‌.

Most importantly, we discovered a new regulatory molecule of cGAS within the nucleus. Our study identified H1.2 as a crucial negative regulator of nuclear cGAS, promoting cGAS binding to chromatin by affecting the cGAS Lys240 site activation and maintaining cGAS in a highly inactive state. Furthermore, H1.2 recruited the ubiquitin ligase TRIM28, facilitating the interaction between TRIM28 and cGAS, leading to the degradation of inactive cGAS in the nucleus. Our ubiquitination mass spectrometry results show that TRIM28 brings K48-linked ubiquitination at K261 and K424 sites of cGAS, promoting the degradation of the cGAS proteasome pathway. We found that lysine mutations at either the K261 or K424 site can attenuate the H1.2 overexpression-induced enhancement of K48-linked polyubiquitination of cGAS, demonstrating that H1.2 promotes nuclear cGAS degradation by recruiting TRIM28 and targeting the K261 and K424 sites of cGAS. Furthermore, we found that both the K261R and K424R mutants of cGAS can partially rescue the inhibitory effects of H1.2 overexpression on the expression of Ifnb and Isg15. This suggests that the H1.2-TRIM28-cGAS degradation pathway is also a crucial component in the regulation of cGAS function mediated by H1.2. The degradation of nuclear cGAS similarly affects the intracellular cGAS-mediated IFN response against HSV-1 infection, which may be attributed to the reduced release of cGAS into the nucleosol and cytoplasm following nuclear cGAS degradation.

There are still limitations in our research: first, although pull-down assay and Co-IP data can confirm the physical interaction between H1.2 and cGAS, they are limited by the antibody recognition area and precipitation efficiency and cannot distinguish between the direct binding interface and the indirect association site. This limitation is particularly common in the study of complex dynamic interaction. In the future, cross-linked mass spectrometry (XL-MS) or cryoelectron microscopy is needed to analyze the contact interface of H1.2-cGAS. Second, this study confirmed the molecular mechanism of TRIM28 based on human cell lines, while the mouse model was used for *in vivo* function verification. Although it is found that this TRIM28 is highly conserved among human and mouse species (65), species-specific differences may still exist, which is a technical limitation of this study. In addition, our study currently demonstrates that H1.2 interacts with cGAS, enhances cGAS-chromatin binding through its interaction with cGAS, and recruits TRIM28 to promote ubiquitination-mediated degradation of cGAS at lysine residues K261 and K424, thereby modulating cGAS-dependent immune responses during HSV-1 infection. However, we have not yet elucidated the precise mechanism by which H1.2 stabilizes the cGAS-nucleosome structure, nor confirmed the spatial and structural regulation of H1.2 on cGAS conformational activity, or comprehensively proven the temporal dynamics of the H1.2-cGAS-nucleosome complex throughout the cell cycle. Furthermore, as represented by H1.2, the regulation of cGAS by H1 histones appears to be a shared characteristic among certain H1 subtypes. Do other members of the H1 family regulate cGAS function through the same mechanism as H1.2, and are the regulatory effects of these members synergistic or compensatory? Besides, whether the negative regulatory function of H1.2 on cGAS is a common feature among H1 histone members remains to be investigated. Finally, Sp1’s regulation of replication-dependent histones is a broad process. How host cells downregulate Sp1 during infection, how Sp1 thereby controls specific H1 subtypes to impact downstream IFN responses, and whether there are other transcription factors involved in the co-regulation of other histone H1 subtypes in this regulation process remain unclear. These will be the focus of our next research.

Given the cooperative role of H1.2 and cGAS in various biological functions, we proposed H1.2 works as an important negative regulatory factor of cGAS, promoting cGAS to remain inactive to avoid being activated by host self-dsDNA in the nucleus and causing chronic inflammation. H1.2-cGAS axis may represent a broad regulatory pathway involved in numerous physiological and pathological processes. In our follow-up research, we will further study the regulatory relationship between H1.2 and cGAS in different pathological states, which will complement the puzzle of human innate immunity theory.

**Fig 8:**
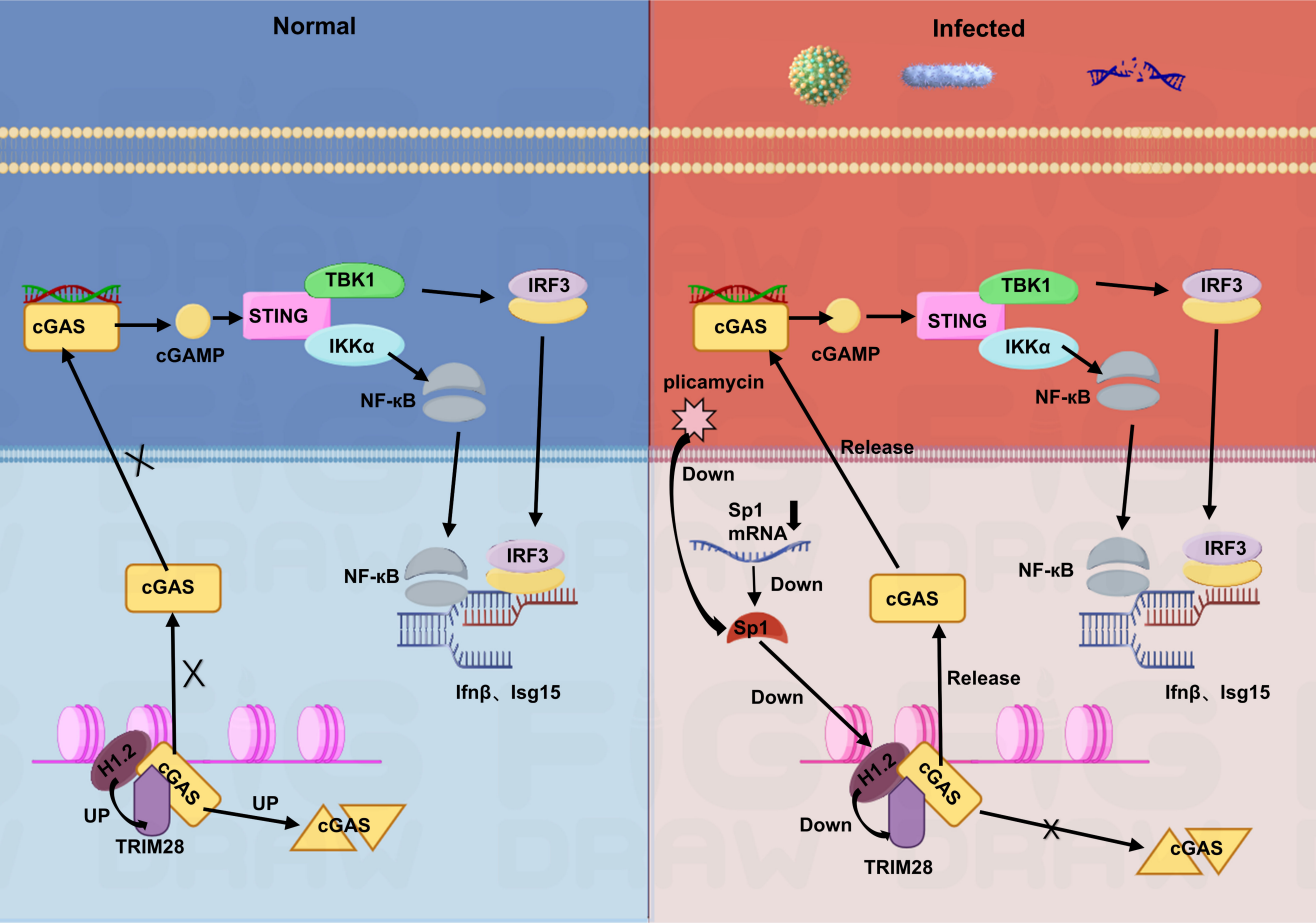
Diagram depicting the plicamycin-Sp1-H1.2-cGAS-STING-TBK1-IRF3-type I IFN axis.

In summary, our study identified H1.2 as a new negative regulator of cGAS in the nucleus, influencing antiviral immunity through the Sp1-H1.2-cGAS axis. Additionally, we uncovered the potential of plicamycin as an antiviral drug (Fig. 8). Thus, our research has illuminated a previously undiscovered mechanism of immune regulation by cGAS and highlighted the possibility of H1.2 as a target.

## MATERIALS AND METHODS

### Reagents and plasmids

The following antibodies were used in this study: anti-TBK1 (3504), anti-phospho-TBK1 (5483), anti-phospho-IRF3 (29047), anti-IRF3 (4302), anti-phospho-IRF7 (24129), anti-IRF7 (72073), anti-phospho-p65 (3033), anti-p65 (8242), anti-phospho-p38 (9215), anti-p38 (8690), anti-phospho-Erk1/2 (9101), anti-Erk1/2 (4695), anti-STING (13647), anti-cGAS (15102), and anti-phospho-STING (72971), all from Cell Signaling Technology; anti-cGAS (A8385), anti-P62 (A19700), anti-TRIM28 (A19568), anti-Sp1 (A19649), anti-α-tubulin (AC007) from Abclonal; anti-H1.2 (19649-1-AP), anti-GFP (50430-2-AP), anti-Myc (16286-1-AP), anti-HA (81290-1-RR), anti-Flag (66008-4-Ig); all from Proteintech; anti-GST (CW0085M) and anti-His (CW0083M), both from Abclonal.

Expression constructs for Flag-cGAS, HA-cGAS, and HA-Ubs were obtained from Dr. B. Ge (Tongji University, Shanghai, China), and Flag-STING was obtained from Dr. B. Sun (Shanghai Institute of Biochemistry and Cell Biology, Shanghai, China). H1.2 and TRIM28 were inserted into pcDNA3.0 and pEGFP-C1 vectors. Site-directed point mutagenesis of cGAS was performed using the Mut Express MultiS Fast Mutagenesis Kit V2 (C215-01, Vazyme) according to the manufacturer’s instructions. Small interfering RNAs were synthesized by GenePharma (Shanghai, China). The primer sequences used to amplify human and mouse genes are described in Table S1.

### Cells and viruses

Mouse peritoneal macrophages, MEFs, L929, and HEK293T cells were maintained in Dulbecco’s modified Eagle’s medium (DMEM; Hyclone) supplemented with 10% (vol/vol) heat-inactivated FBS (Gibco) and 100 U/mL penicillin and streptomycin (Hyclone). HeLa cells were cultured in RPMI-1640 medium supplemented with 10% (vol/vol) heat-inactivated FBS (Gibco) and 100 U/mL penicillin and streptomycin (Hyclone). Vero cells were cultured in DMEM supplemented with 3% (vol/vol) heat-inactivated FBS (Gibco). HSV-1 was obtained from Dr. B. Ge (Tongji University, Shanghai, China), cultured, or titer determined by Vero cells.

### Mouse strains

Homozygous H1F2^−/−^ mice (from Cyagen Biosciences, Suzhou, China) were bred under specific pathogen-free conditions at the Shanghai Medical College Experimental Animal Research Center for Biomodel Organisms. Six-week-old male-specific pathogen-free (SPF) H1F2^−/−^ mice and their WT littermates were used in the experiments.

### Isolation of mouse peritoneal macrophages and MEFs

For preparing mouse peritoneal macrophages, 6-week-old homozygous H1F2^−/−^ mice and their WT littermates were injected with 1.5 mL *Brucella* broth (4%) intraperitoneally. After 3 days, the peritoneal lavage fluid was collected from the mice and washed three times with PBS. Peritoneal macrophages were cultured in DMEM supplemented with 10% FBS. For the preparation of MEFs, 13-day-old embryos of WT C57BL/6 mice were digested without head and entrails using type IV collagenase (17104019, Gibco) at 37°C for 1 h and terminated with PBS. The digested samples were ﬁltered with a 200-mesh sieve and then centrifuged at 25°C and 1,000 rpm for 3 min. The cell pellets were suspended and cultured in DMEM supplemented with 10% FBS and 100 U/mL penicillin and streptomycin for 24 h. The cells were continuously cultured for passage or frozen when they had reached 90%–95% conﬂuence.

### Virus infection

For *in vitro* virus infection, L929, HeLa, HEK293T, MEF cells, and peritoneal macrophages (2 × 10^6^ cells) were cultured in DMEM for 12 h and infected with HSV-1 (MOI = 10) for the indicated times. For *in vivo* virus infection, 6-week-old homozygous H1F2^−/−^ mice and their WT littermates were injected with HSV-1 (2 × 10^8^ PFU/mouse) for the indicated times, or a high dose of HSV-1 (2 × 10^10^ PFU/mouse) for survival detection.

### Plasmids and siRNA transfection

Plasmids were transiently transfected with polyethylenimine (PEI, 23966-2, Polysciences) according to the manufacturer’s instructions. HEK293Ts, L929s, and MEFs were used for transfection experiments. For transfection dose, the total amount was 2 μg/six-well plate per well. The transfection system was 200 μL serum-free medium/six-well plate per well. For DNA, PEI = 1:3. After transfection for 4–6 h, a new 10% medium was replaced. Samples were collected 48 h after transfection.

L929 cells were used for transfection of small interfering RNA (sequence supplement in Table S1). Small interfering RNAs were transfected with CALNP RNAi *in vitro* (D-nano Therapeutics, DN001) according to the manufacturer’s instructions. Transfection concentration was 50 nM. After 24 h of transfection of siRNA, a plasmid feeding experiment was carried out. After transfection of the plasmid for 24 h, HSV-1 was added to stimulate the corresponding time point. Empty vectors or siNC were used to ensure that the total amount of transfected plasmids or siRNA in the control group and the experimental group was the same.

### Ubiquitin experiment *in vitro*

UB, E1, and E2 (UBE2D) were purchased from YEASON recombinant human ubiquitin-binding enzyme screening kit (20440ES10). cGAS and TRIM28 were overexpressed and purified in HEK293T cells. The configuration of the reaction system is guided by the instructions. The configuration of the reaction system is guided by the instructions. After incubation at 37°C for 30 min, 5× loading buffer was added, and SDS-PAGE was used to measure the degree of ubiquitination.

### CCK8 analysis

293T cells or L929 cells were transfected with vector or target gene. After 48 h, the cells were digested and plated in 98-well plates. After the cells adhered to the wall, CCK-8 detection reagent was added. Cell Counting Kit-8 was purchased from Beyotime (C0037), and the experimental method was carried out according to the instructions.

### Mass spectrum

Protein binding mass spectrometry: HEK293T cells overexpressed GFP and cGAS (Fig. S3E) or GFP-H1.2 and cGAS (Fig. S6A), respectively. After 48 h, cells were lysed, cGAS protein was purified by the IP method in the cell lysate, and then samples were prepared by SDS-PAGE and Coomassie brilliant blue staining. Subsequently, the samples were identified and analyzed by liquid chromatography-tandem mass spectrometry and LC-MS/MS, and the identification and analysis service was provided by Nomi Biotechnology.

Protein ubiquitination modified mass spectrometry: TRIM28 and cGAS were overexpressed in L929 cells. After 48 h, cells were lysed, cGAS protein was purified by the IP method in the cell lysate, and then samples were prepared by SDS-PAGE and Coomassie brilliant blue staining. Subsequently, the samples were identified and analyzed by liquid chromatography-tandem mass spectrometry and LC-MS/MS, and the identification and analysis service was provided by Applied Protein Technology.

### Quantitative real-time PCR

Cells were incubated for 12 h with DMEM and then infected with the viruses for the indicated times. Total RNA was isolated using RNAiso Plus (9109, Takara) according to the manufacturer’s instructions. Then 1 µg of RNA was reverse-transcribed using the HiScript IV RT SuperMix for qPCR (R423-01, Vazyme) to generate cDNA. The LightCycler (LC480, Roche) and SYBR RT-PCR Kit (Q411-02/03, Vazyme) were used for quantitative real-time PCR analysis. Gene amplification was performed using the ΔΔCt method. Gene expression was normalized to that of GAPDH. The primer sequences used to amplify human and mouse genes are described in Table S1.

### Immunoprecipitation and western blotting

Cells were transfected with the indicated plasmids. After 48 h, cells were lysed in a lysis buffer (50 mM Tris [pH 7.4], 150 mM NaCl, 1% Triton X-100, and 1 mM EDTA [pH 8.0]) supplemented with a protease cocktail (Roche, 04693159001) consisting of 1 mM PMSF, 1 mM Na_3_VO_4_, and 1 mM NaF for 30 min on ice. The lysates were centrifuged at 13,200 rpm for 15 min at 4°C to remove the debris. Cell lysates were incubated with anti-Flag M2 Affinity Gel or Protein A/G Sepharose Fast Flow plus prespecified antibodies at 4°C overnight. For immunoprecipitation of endogenous protein, mouse peritoneal macrophages or L929 cells were lysed and incubated with the specified antibodies and Protein A/G Sepharose at 4°C overnight. The sepharose samples were centrifuged and washed three times with ice-cold PBST buffer (1% Triton X-100 in PBS). Precipitates or cell lysates were boiled in 1× SDS loading buffer at 100°C for 10 min and then analyzed by immunoblotting.

### GST pull-down

His or GST fusion proteins were expressed in BL-21(DE3) (Tiangen Biotech) according to the manufacturer’s instructions. The bacteria were collected by centrifugation and ultrasonically cracked in the lysate (1% Triton X-100 in PBS) with benzonase (BeyoZonase) added (according to the instructions, 25 U/mL, room temperature, 30 min). GST-fusion proteins were incubated with GST beads for 4 h at 4°C, and then washed three times with TBST to collect the precipitates. Ni column (SA005GC, Smart-Lifesciences, Shanghai, China) purified His-fusion protein or lysates of L929s were incubated with the collected precipitates for 4 h at 4°C. After centrifugation and washing, the beads were boiled in 1× SDS loading buffer at 100°C for 10 min and then analyzed by immunoblotting.

### ELISA

The primary cells of mice stimulated by virus for a specific time or the peripheral blood of mice infected by virus were treated according to the requirements of the kit instructions, and the content of cGAMP (EN-XS94549, BIOSCIENCES; CB15107, COIBO BIO)/IFNb (ml001982, mlBio) was determined by kits.

### Native PAGE

The dimerization assay was performed as described. In brief, mouse peritoneal macrophages or L929 cells were cultured in DMEM for 12 h and infected with the virus for the indicated times. Cells were harvested using 100 μL ice-cold lysis buffer (50 mM Tris, pH 7.4; 150 mM NaCl; 1% Triton X-100; 1 mM EDTA, pH 8.0; and a protease inhibitor cocktail consisting of 1 mM PMSF, 1 mM Na_3_VO_4_, and 1 mM NaF). After centrifugation at 13,000 × *g* for 15 min at 4°C, the supernatants were quantified and diluted with 2× native PAGE sample buffer (125 mM Tris-HCl, pH 6.8; 30% glycerol; and 0.1% bromophenol blue). Then, 30 μg of protein was applied to a pre-run 7.5% native gel. After electrophoresis, the proteins were transferred onto a nitrocellulose membrane for immunoblotting.

### Dual-luciferase reporter assay

HEK293T cells were transiently transfected with pRL-IFN-β–Luc or pRL-ISRE–Luc, pRL-TK, and the indicated plasmids for 24 h. The dual-luciferase reporter assay system (RG028, Beyotime) was used to detect the luciferase activity according to the manufacturer’s instructions.

### Cell staining and confocal microscopy

Peritoneal macrophages were infected with viruses directly. Cells were fixed with 4% formaldehyde for 20 min at room temperature, permeabilized for 30 min in PBS containing 0.3% Triton X-100, and then blocked for 1 h at 4°C in a blocking buffer (1% BSA in PBS). Then, the cells were incubated with the indicated antibodies at 4°C overnight and secondary antibodies at room temperature for 1 h. After staining with DAPI, images were obtained using a Leica LAS X microscopy system. The statistics of positive transposed cells were counted with the help of Image J, and the significance of the data was analyzed by PRISM9.

HeLa cells were transfected with the appropriate plasmids for 48 h and infected with HSV-1 for the indicated times. Cells were fixed with 4% formaldehyde for 20 min at room temperature, permeabilized for 30 min in PBS containing 0.3% Triton X-100, and then blocked for 1 h at 4°C in a blocking buffer (1% BSA in PBS). After staining with DAPI, images were obtained using a NOVEL NCF950 Laser confocal microscope. Image analysis is completed by NPX-C software.

### Electrophoretic mobility shift assay

To conduct the electrophoretic mobility shift assay (EMSA), we first prepared the lysates of BL-21(DE3) transformed with H1.2 and cGAS plasmids for the DNA–protein conjugation reaction and measured the protein concentration by BCA protein assay kit (P0012, Beyotime). We synthesized biotin-labeled ISD probe for EMSA assay (Sangon Biotec) and executed the DNA–protein binding reactions using EMSA Gel-Shift Kit (GS009, Beyotime) following the manufacturer’s instructions at 25°C. Finally, the reaction mixture was separated through 6% non-denaturing PAGE gels and developed.

### Cell component separation assay

Cells transfected with indicated plasmids were collected in cold PBS and washed three times (3,000 rpm, 4°C, 5 min). Then the collection was incubated in 4°C with buffer E1 (50 mM HEPES-KOH, pH 7.5; 140 mM NaCl; 1 mM EDTA, pH 8.0; 10% glycerol; 0.5% NP-40; 0.25% Triton X-100; 1 mM DTT; 1× protease inhibitor cocktail) and centrifuged. The supernatant was collected, and the deposit was treated with buffer E2 (10 mM Tris-HCl, pH 8.0; 200 mM NaCl; 1 mM EDTA; 0.5 mM EGTA; 1× protease inhibitor cocktail), and then incubated and centrifuged. Then the supernatant was collected, and the deposits were treated with buffer E3 (500 mM Tris-HCl, pH 6.8; 500 mM NaCl; 1× protease inhibitor cocktail). The chromatin fraction was either sonicated to shear the DNA or treated with benzonase to digest nucleic acids. The purity of each fraction was controlled by Western blot for α-tubulin, lamin B, and histone H3 to depict the cytoplasmic, nuclear, and chromatin fraction, respectively.

### Protein-protein interaction analysis

This study employed the GRAMM platform to perform rigid-body docking experiments for target proteins. This docking mode maintains the three-dimensional structures of both ligand and receptor proteins as rigid bodies (i.e., no conformational changes occur), using algorithms to search the receptor surface for the optimal geometric fit with the ligand. The experimental workflow was as follows: target protein structures were retrieved from the UniProtKB database using known gene names, and then both target protein structures were input into the GRAMM system. Calculations were initiated using the platform’s default protein-protein docking parameters. Upon completion, the top 10 docking results (automatically ranked by binding efficacy, with Rank 1 representing the theoretically optimal conformation) were collected. Binding energy served as the core evaluation criterion (industry standard: <−4 kcal/mol; lower values indicate greater complex stability). The top-ranked docking conformation was further analyzed using metrics including interaction surface area, hydrogen bonding, and key amino acid residues. Following docking, binding free energy was calculated using PDBePISA and visualized with PyMOL 3.1.

### Statistical analysis

Data are expressed as data are presented as mean ± SD. Prism 9 (GraphPad) was used for statistical analysis. The statistical tests conducted in this study are indicated in the figure legends as follows: *P < 0.05, **P < 0.01, ***P < 0.001, two-tailed unpaired Student’s *t*-test. Leica LAS X microscopy system microscopy system is used for immunohistochemistry data. ViiA 7 Software v1.2.4 on ViiA 7DX is used for qRT-PCR data. ImageQuant LAS 4000 mini and Amersham Imager 600 are used for western blot data.

## SUPPLEMENTAL MATERIAL

Supplemental material mbio.03881-25-s0001.docx Figures S1 to S7, Table S1, and results of protein spectrum experiments.

ASM does not own the copyrights to Supplemental Material that may be linked to, or accessed through, an article. The authors have granted ASM a non-exclusive, world-wide license to publish the Supplemental Material files. Please contact the corresponding author directly for reuse.
