# Supplementary material for: Linker histone H1.2 inhibits HSV-1-induced IFN response via cGAS
Source: mBio. 2026 Apr 13;17(5):e03881-25. doi: 10.1128/mbio.03881-25 (PMC13170280; doi:10.1128/mbio.03881-25)
Supplement: Supplemental material — Figures S1 to S7, Table S1, and results of protein spectrum experiments. [file mbio.03881-25-s0001.docx]

***Supplementary Material***

**Linker histone H1.2 inhibits HSV-1-induced IFN response via cGAS.**

This file includes:

Figures of S1 to S8

Table S1

**Supplementary Figures**


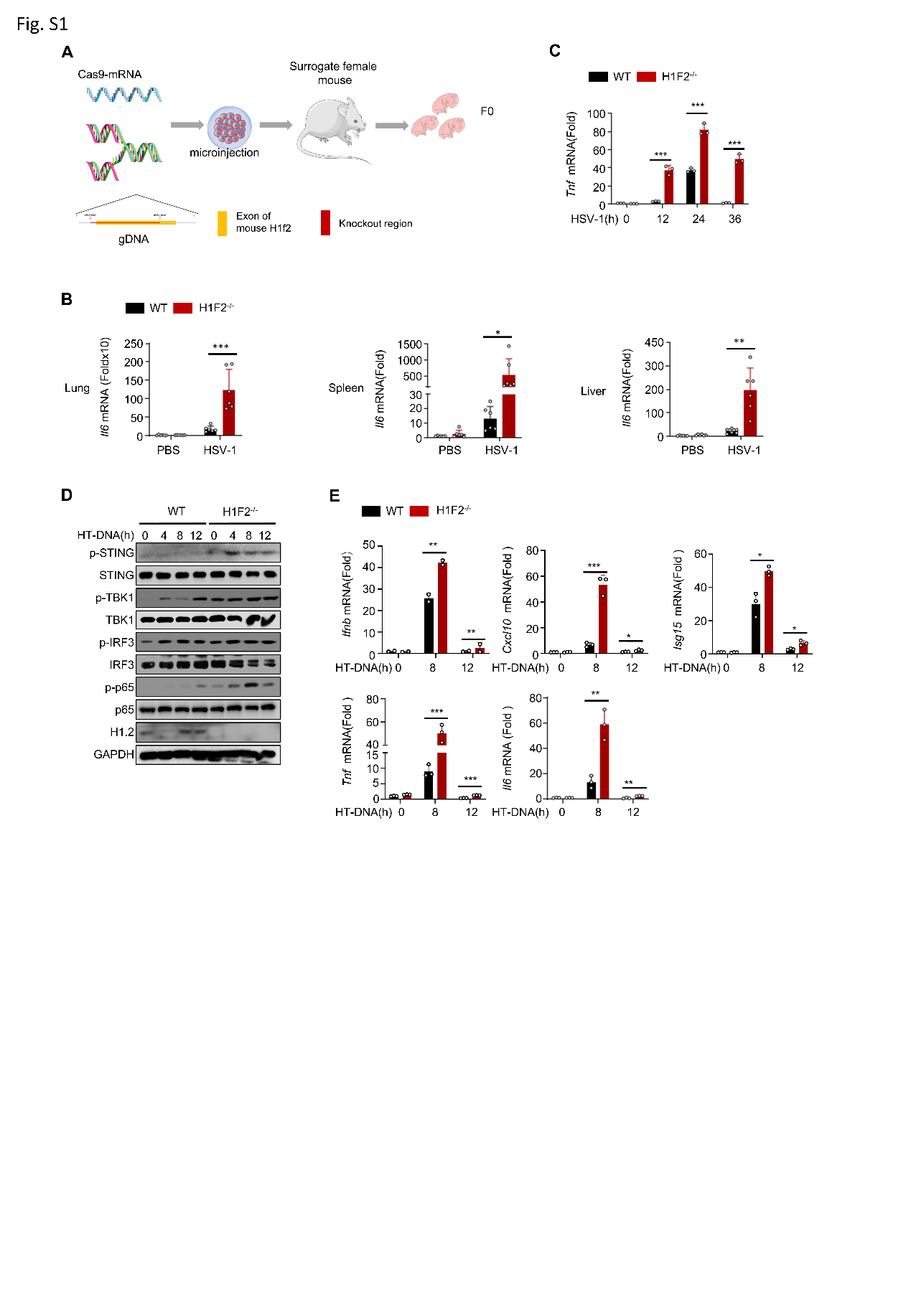


**Supplementary Figure 1. H1.2 deficiency enhances antiviral immune response. (A)** Construction of H1F2^−/−^ mice. **(B)** Il6 mRNA level of lung, spleen and liver of WT or H1F2^−/−^ mice infected with HSV-1. **(C)** Tnf mRNA levels in peritoneal macrophage of WT or H1F2^−/−^ mice treated with HSV-1 for indicated times (n = 3). **(D)** Immunoblot analysis of lysates of peritoneal macrophage of WT or H1F2^−/−^ mice treated with HT-DNA for indicated times. **(E)** Ifnb, Tnf, Il6, Isg15 and Cxcl10 mRNA levels in peritoneal macrophage treated as in **(D)** (n = 3). Data are means ± SD. *P < 0.05, **P < 0.01, ***P < 0.001 (two-tailed unpaired Student’s t-test).

**
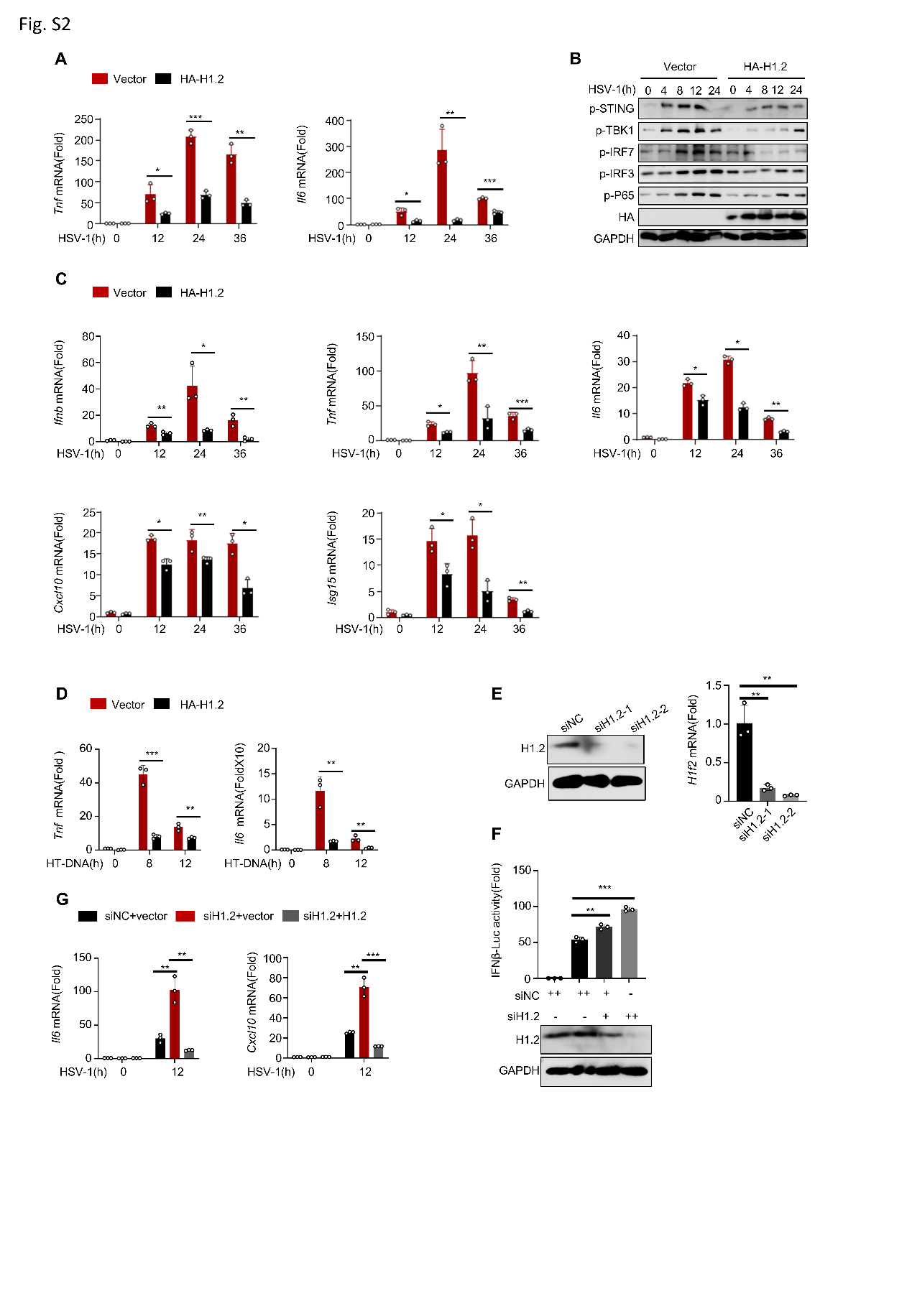
**

**Supplementary Figure 2. H1.2 negatively regulates IFN-β signaling. (A)** Tnf, and Il6 mRNA levels in L929s treated as in **(Fig.2C)**. **(B)** Immunoblot analysis of lysates of MEFs overexpressing vector or H1.2 and infected with HSV-1 for indicated times. **(C)** Ifnb, Tnf, Il6, Isg15, and Cxcl10 mRNA levels in MEFs expressing vector or H1.2 treated as in **(B)**. **(D)** Tnf, and Il6 mRNA levels in L929 cells treated as in **(Fig.2F)**. **(E)** Validation of siH1.2. 24 hours after L929 cells were transfected with siNC or siH1.2, RNA was extracted to detect the changes of transcription level of H1.2, and 48 hours after transfection, the cell contents were extracted to detect the changes in protein level. SiH1.2-2 was used for further detection. **(F)** Luciferase assay of IFN-β of L929 cells transfected with different dose of siH1.2-2 and IFN-Luc, TK or vector, and infected with HSV-1 for 4 hours. SiNC was used to ensure that the total amount of transfected siRNA was same. Mock only transfected with siNC. **(G)** Cxcl10 and Il6 mRNA levels in L929 cells treated as in **(Fig.2I)**. Data are means ± SD. *P < 0.05, **P < 0.01, ***P < 0.001 (two-tailed unpaired Student’s t-test).


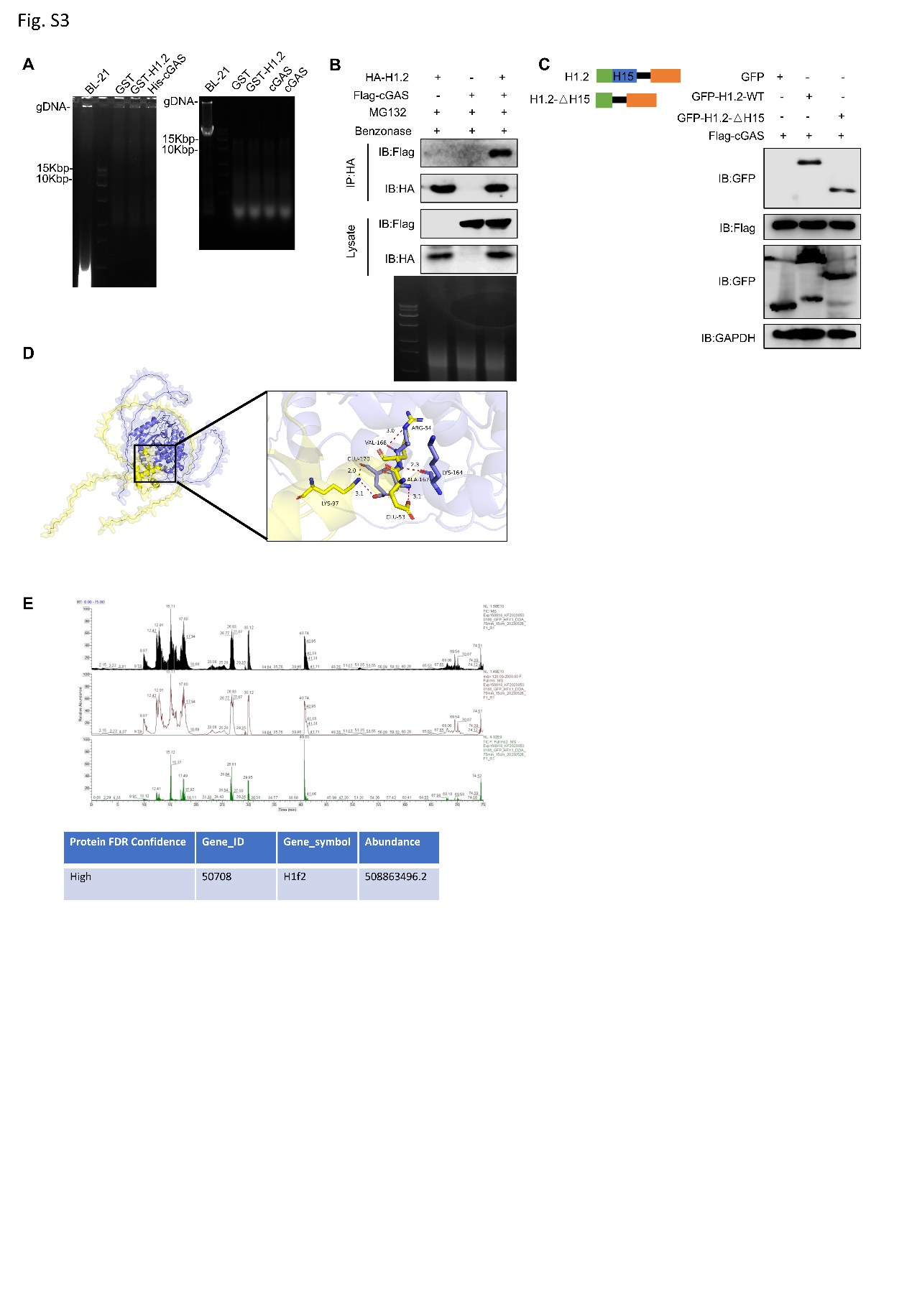


**Supplementary Figure 3.** **H1.2 interacts with cGAS in the nucleus.** **(A)** The validation of benzonnase digestion effect is related to FIG.3E (Left) and 3F (Right) (25U/ml, 25℃, 30min). **(B)** Co-Immunoprecipitation analysis in 293T cells transfected with HA-H1.2 and Flag-cGAS. **(C)** Construction of H1.2 DNA-binding defective mutant (left) and immunoprecipitation results of H1.2 DNA-binding defective mutant with cGAS (Right) in HEK293Ts. **(D)** Protein-protein docking and docking site analysis in Surface form of cGAS (purple) and H1.2 (yellow). **(E)** Mass spectrogram results of HEK293T cells overexpressed GFP and cGAS.


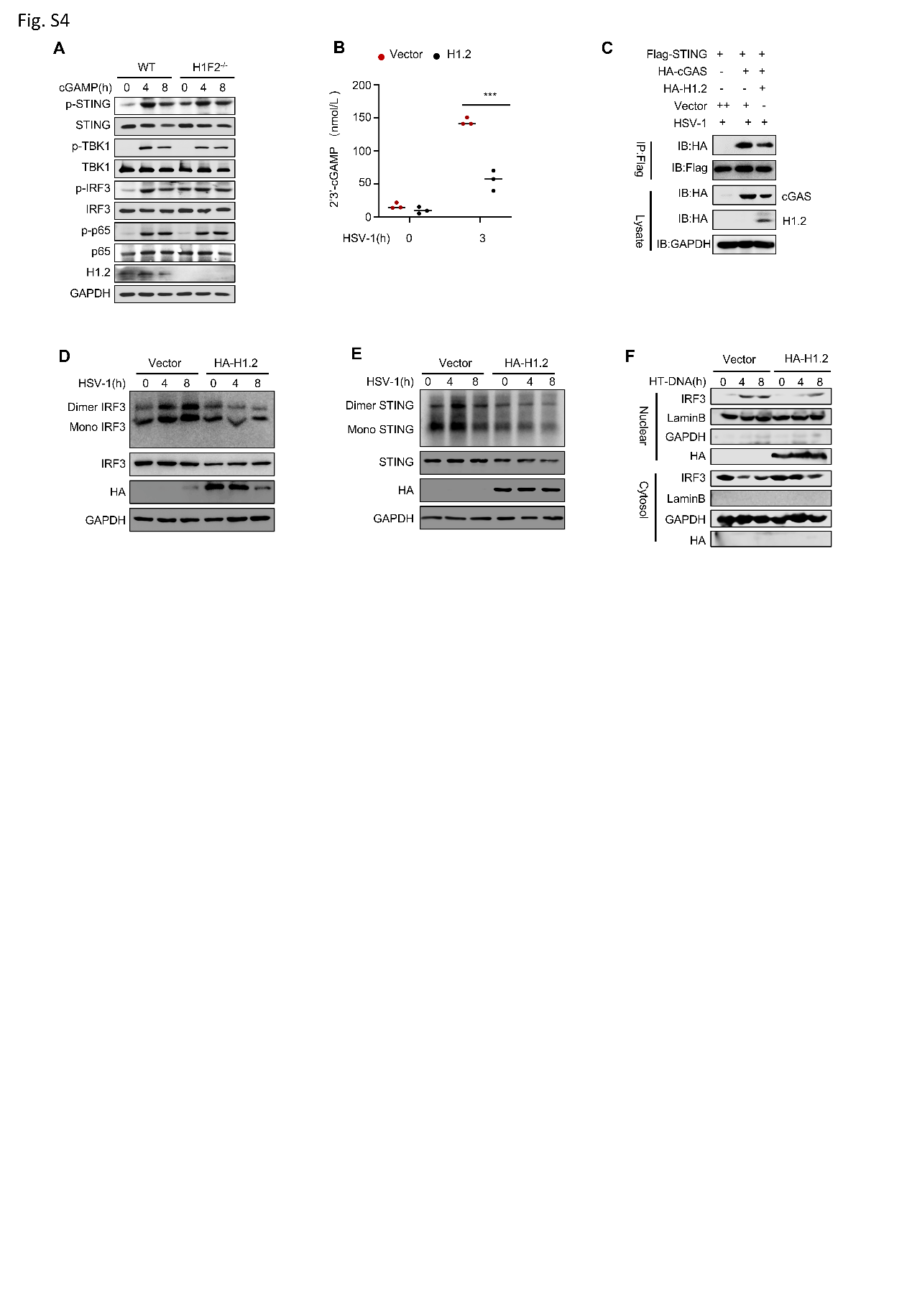


**Supplementary Figure 4. H1.2 inhibits cGAS activation. (A)** Immunoblot analysis of lysates of peritoneal macrophage of WT or H1F2^−/−^ mice treated with cGAMP for indicated times. **(B)** Elisa assay of cGAMP of L929s overexpressing vector or H1.2. **(C)** Coimmunoprecipitation assay of binding of STING and cGAS. **(D, E)** Dimerization assay of STING **(E)** and IRF3 **(D)** in L929 cells overexpressing control vector or H1.2 and treated with HSV-1 for indicated times. **(F)** Immunoblot analysis of nuclear and cytoplasmic fractions in L929 cells overexpressing control or H1.2 and treated with HT-DNA for indicated time.


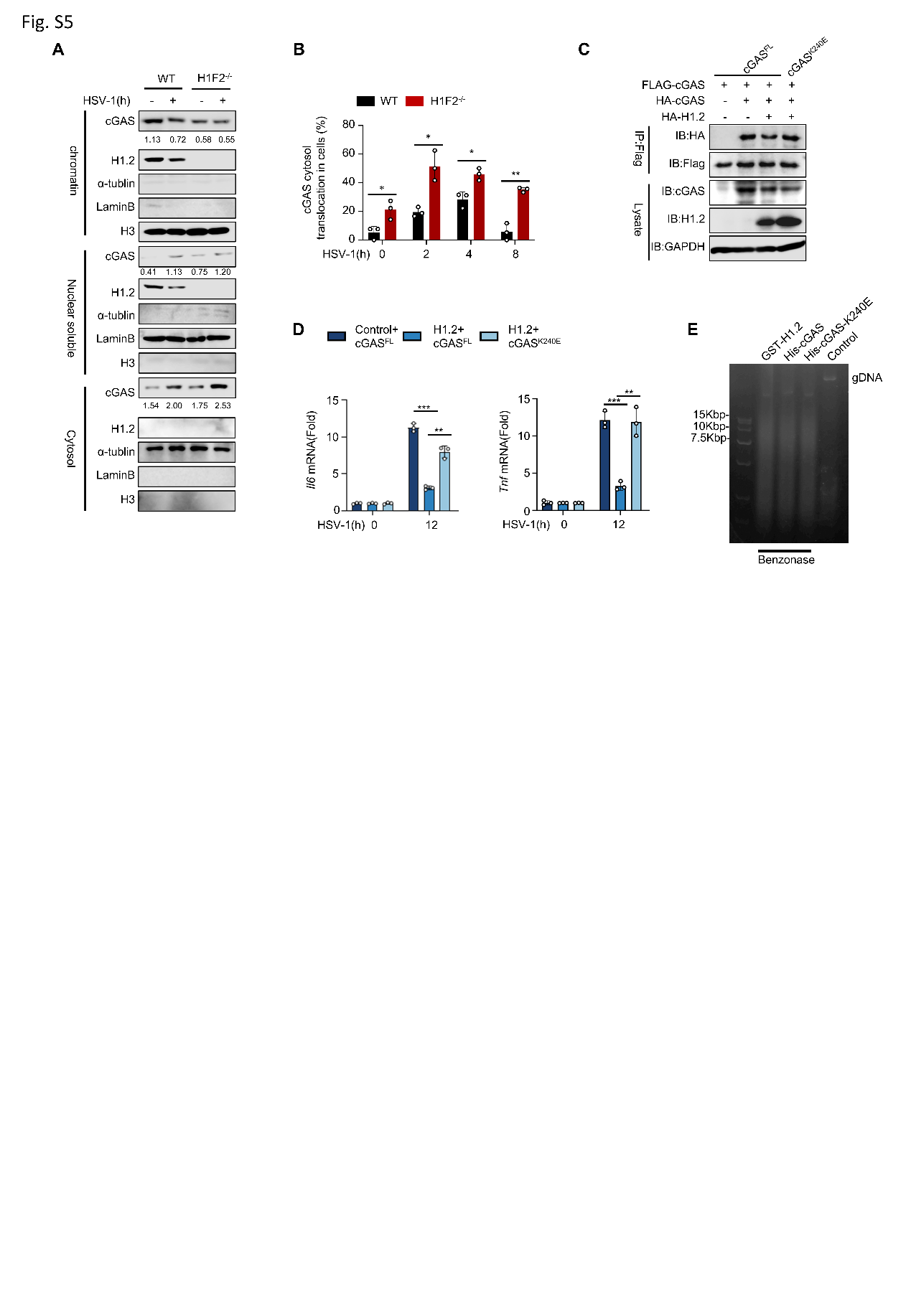


**Supplementary Figure 5.** **H1.2 ties cGAS to chromatin.** **(A)** Immunoblot analysis of chromatin, nuclear soluble and cytoplasmic fractions of WT or knockout peritoneal macrophage infected with HSV-1 for indicated times. **(B)** Quantitative data of peritoneal macrophages from WT or H1F2^−/−^ mice treated as in **(Figure.5B)**. **(C)** Immunoprecipitation analysis of HEK293T cells transfected with indicated plasmids. **(D)** Tnf and Il6 mRNA level of cGAS^-/-^ MEFs transfected with indicated plasmids and infected with HSV-1 (n = 3). **(E)** The validation of benzonnase digestion effect is related to FIG.5I (25U/ml, 25℃, 30min). Data are means ± SD. *P < 0.05, **P < 0.01, ***P < 0.001 (two-tailed unpaired Student’s t-test).

**
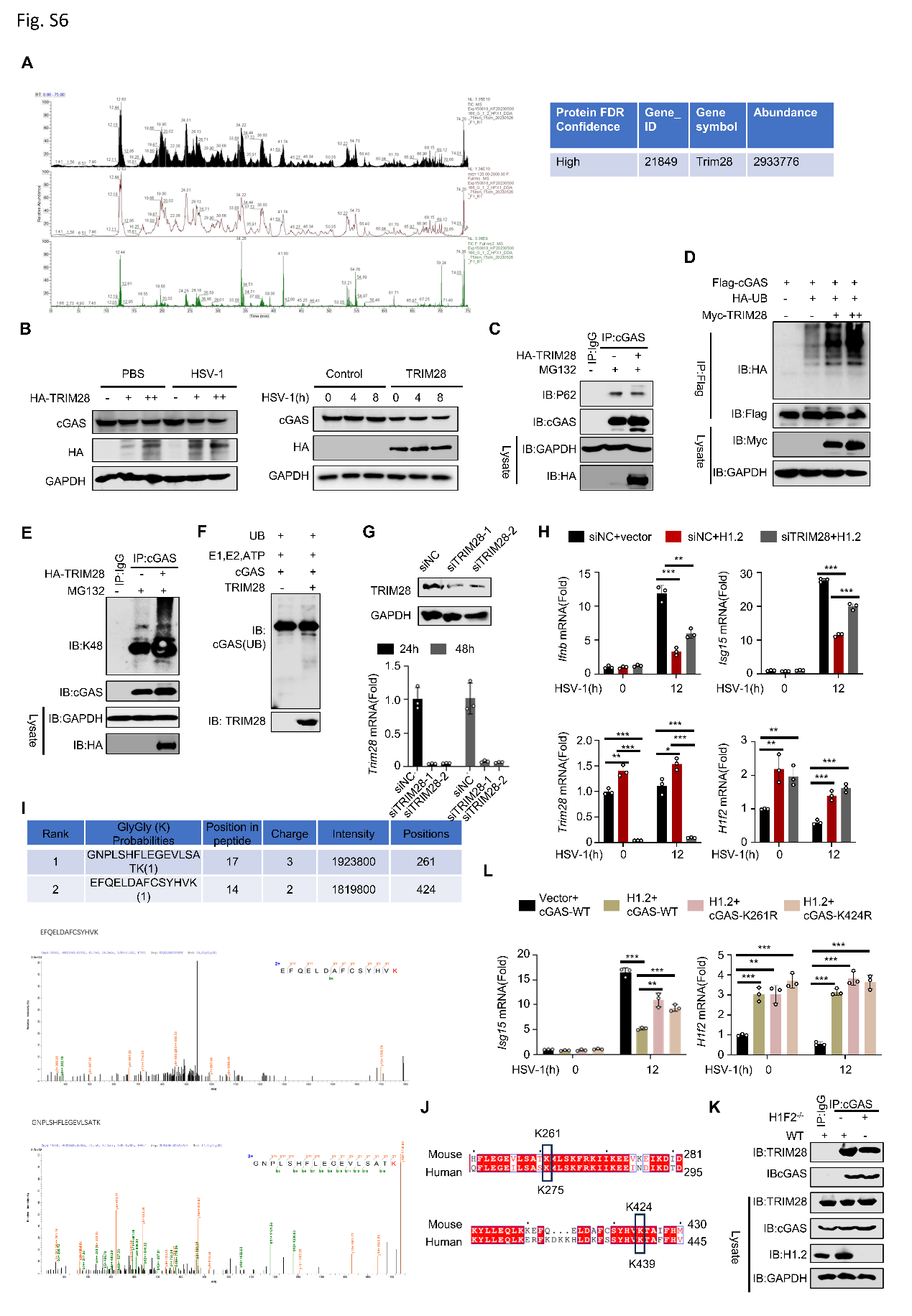
**

**Supplementary Figure 6. H1.2 degrades inactive cGAS by recruiting TRIM28. (A)** The results of mass spectrometry ion current and TRIM28 abundance of HEK293T cells overexpressed with H1.2 and cGAS. **(B)** Immunoblot analysis of cGAS expression levels in L929s overexpressing different dose of TRIM28(Left) and endogenous immunoblot analysis of L929 cells transfected with or without TRIM28 and treated with HSV-1 for indicated times (Right). **(C)** Endogenous co-immunoprecipitation analysis of L929s transfected with or without TRIM28 and treated with MG132(10μM,6h). **(D)** Immunoprecipitation analysis of ubiquitination levels of cGAS in HEK293T cells overexpressing different dose of TRIM28. **(E)** Immunoprecipitation analysis of endogenous K48-linked ubiquitination levels of cGAS affected by TRIM28 in L929 cells. **(F)** TRIM28 promotes the ubiquitination of cGAS in vitro. **(G)** Validation of siTRIM28. 24 hours after transfection, samples were collected to detect the expression of Trim28 mRNA and 48 hours after transfection, samples were collected to detect the knock-down effect of protein. SiTRIM28-1 was used in the subsequent knock-down experiment. **(H)** Analysis of mRNA levels of *Ifnb, Isg15, Trim28* and *H1f2* in L929s transfected with indicate siRNA and plasmids. **(I)** Ubiquitin-modified mass spectrometry of cGAS in L929 cells overexpressed TRIM28 and cGAS. **(J)** Conservative analysis of cGAS lysine loci in human and mouse. **(K)** Endogenous co-immunoprecipitation analysis of PMs from WT or H1F2^-/-^ mice. **(L)** Analysis of mRNA levels of *Isg15, H1f2* in cGAS^-/-^ MEFs transfected with indicate plasmids. Data are means ± SD. *P < 0.05, **P < 0.01, ***P < 0.001 (two-tailed unpaired Student’s t-test).


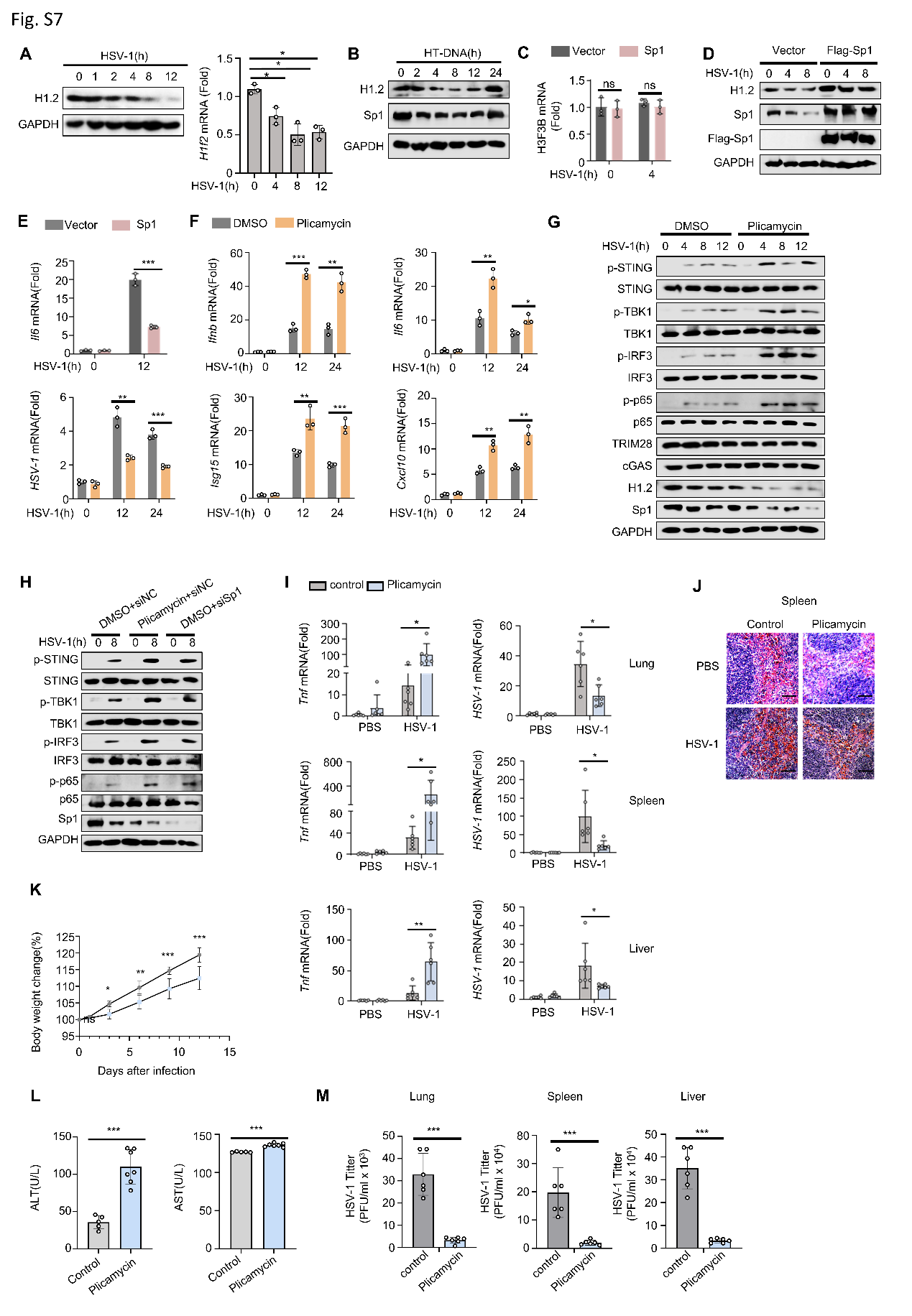


**Supplementary Figure 7.** **Plicamycin enhances antiviral immunity through the Sp1-H1.2-cGAS axis. (A)** Immunoblot analysis of H1.2 protein level of HEK293T cells infected with HSV-1(Left) and H1f2 mRNA level of HEK293T cells treated with HSV-1 for indicated times (Right). **(B)** Immunoblot analysis of H1.2 and Sp1 protein level of PMs infected with HT-DNA. **(C)***H3f3b* mRNA in vector or Sp1 overexpressing L929 cells infected with HSV-1 for indicated times. **(D)** Immunoblot analysis of H1.2 protein level of L929s overexpressed with vector or Sp1. **(E)** *Il6* mRNA levels of L929 cells as in **(Fig. 7F)**. **(F)** *Ifnb, Isg15*, *Il6*, *Cxcl10* mRNA levels and *HSV-1* mRNA level of WT peritoneal macrophage treated as in (**Fig.7G**) **(G)** Immunoblot analysis of lysates of WT peritoneal macrophage treated with or without plicamycin and infected for indicated times. **(H)** Immunoblot analysis of L929s transfected with siRNA and treated with DMSO or plicamycin. **(I)** Tnf and HSV-1 mRNA levels in lungs and spleens and livers as in **(Fig.7J)**. **(J)** Microscopy of hematoxylin-and eosin-stained spleen sections of WT mice treated as in (Fig.7J). **(K)** Weight change curve in the process of survival experiment. **(L)** ALT and AST contents in peripheral blood serum of mice treated as in (Fig.7M). **(M)** HSV-1 titers (n = 6) of tissues of WT mice treated as in **(Fig.7J)**. Data are means ± SD. *P < 0.05, **P < 0.01, ***P < 0.001 (two-tailed unpaired Student’s t-test).


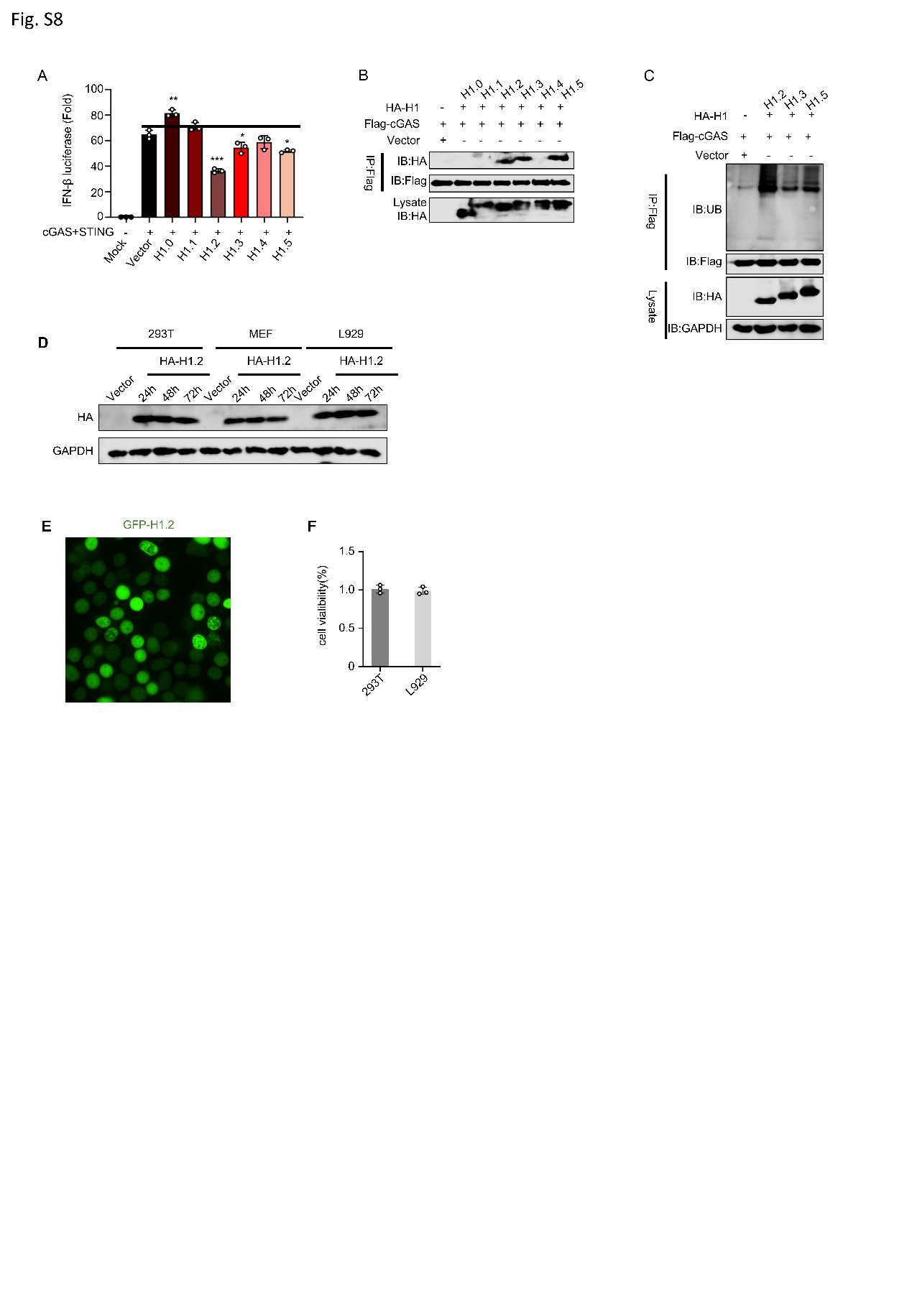


**Supplementary Figure 8.** **The role of H1 family members in cGAS regulation. (A)** Luciferase assay of IFN-βof HEK293T cells transfected with cGAS, STING and vector or H1 family members. The Mock only transfected with vector. **(B)** Coimmunoprecipitation analysis of combination of cGAS and H1 family members in HEK293T.**(C)** Immunoprecipitation analysis of ubiquitination levels of cGAS affected by H1.2,H1.3 or H1.5 in HEK293T cells. **(D)** Verification of transfection efficiency of H1.2. **(E)** Immunofluorescence analysis of GFP-H1.2 in L929s. **(F)** Cck8 analysis of 293T or L929 cells overexpressed vector or H1.2.

**Supplementary table 1**

| qPCR |  |
| --- | --- |
| mIFNb-F | AGCTCCAAGAAAGGACGAACAT |
| mIFNb-R | GCCCTGTAGGTGAGGTTGATCT |
| HSV-1-F | TGGGACACATGCCTTCTTGG |
| HSV-1-R | ACCCTTAGTCAGACTCTGTTACTTACCC |
| mCxcl10-F | TCAGGCTCGTCAGTTCTAAGTT |
| mCxcl10-R | GATGGTGGTTAAGTTCGTGCTT |
| mTNF-F | TTCTGTCTACTGAACTTCGGGGTGATCGGTCC |
| mTNF-R | GTATGAGATAGCAAATCGGCTGACGGTGTGGG |
| m-IL6-F | TCC AGT TGC CTT CTT GGG AC |
| m-IL6-R | GTG TAA TTA AGC CTC CGA CT TG |
| mGAPDH-Y-F | CCC ACT AAC ATC AAA TGG GG |
| mGAPDH-Y-R | CCT TCC ACA ATG CCA AAG TT |
| mISG15-F | CCTCTGAGCATCCTGGTGAG |
| mISG15-R | ACTGGTCTTCGTGGACTTGTT |
| mSp1-F | TGCAAACCAACAGATCATCCC |
| mSp1-R | TGACAGGTAGCAAGGTGATGT |
| hSp1-F | AAGGAACAGAGTGGCAGCAGTAC |
| hSp1-R | GCCTCCACTTCCTCGATTTGT |
| mH1.2-F | AACCCCAGGCTAAGAAGGC |
| mH1.2-R | TGGCTTTACGGCTTTAGACGC |
| hH1.2-F | CCGCCTCTAAAGAGCGTAGC |
| hH1.2-R | AGACCAAGTTTGATACGGCTG |
| hH3.3-F | GTTGGGCGGTGCTGGTTTT |
| hH3.3-R | GGGCCATTTTCTTTCACCCAA |
| hH1.4-F | GACCCCAAAGAAGGCGAAGA |
| hH1.4-R | AGCAGTTGGCCAAAGGAACT |
| mH3.3-F | CTTCGCCGCTCCCCTTTTAT |
| mH3.3-R | TTTCACCGGAAAGGAGGACC |
| mH1.4-F | CTTCCGGCTCGAGTTCTCTC |
| mH1.4-R | AGCCTTGGTGATGAGTTCGG |
| Plasmids |  |
| m-H1.2-F | ATGTCTGAGGCTGCTCCTGC |
| m-H1.2-R | CTACTTTTTCTTGGCTGC |
| h-H1.2-F | ATGTCCGAGACTGCTCC |
| h-H1.2-R | CTATTTCTTCTTGGGCGCC |
| m-Sp1-F | ATGAGCGACCAAGATCACTC |
| m-Sp1-R | TTAGAAACCATTGCCACTGA |
| m-Trim28-F | ATGGCGGCCTCGGCGGCA |
| m-Trim28-R | TCAGGGGCCATCACCAGG |
| siRNA |  |
| siNC-F | UUCUCCGAACGUGUCACGUTT |
| siNC-R | ACG UGC CAC GUU CGG AGA ATT |
| siTRIM28-1-F | CCAAAGACAUCGUGGAGAAUU |
| siTRIM28-1-R | UUCUCCACGAUGUCUUUGGUU |
| SiTRIM28-2-F | GGGAUAUGGCUUUGGGUCAUU |
| SiTRIM28-2-R | UGACCCAAAGCCAUAUCCCUU |
| SiH1.2-1-F | CCUUCAAACUCAACAAGAATT |
| SiH1.2-1-R | UUCUUGUUGAGUUUGAAGGTT |
| siH1.2-2-F | GAAGGUUGCAGCCAAGAAATT |
| siH1.2-2-R | UUUCUUGGCUGCAACCUUCTT |
| siSp1*^[1]^* | CAGCACATTTGTCACATCCAA |

**Supplementary Table 1**. Primers of RT-qpcr, plasmids, dsDNA and siRNA used in article

**References:**

1. Dąbrowska, K. and M. Zielińska. 2019. Silencing of Transcription Factor Sp1 Promotes SN1 Transporter Regulation by Ammonia in Mouse Cortical Astrocytes. *International Journal of Molecular Sciences* 20.

**Protein spectrum results**

**Related to Fig.S3E**

**
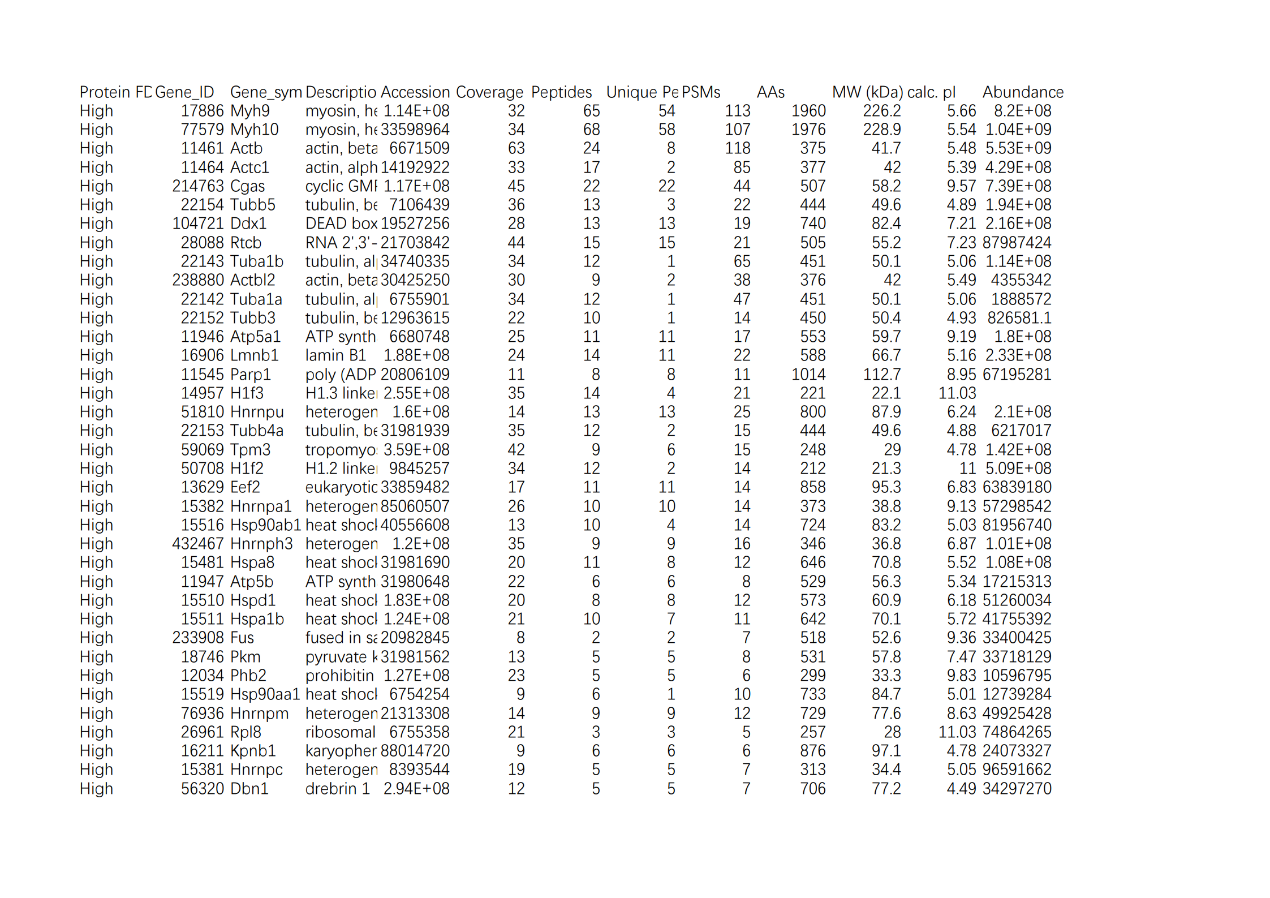
**

**
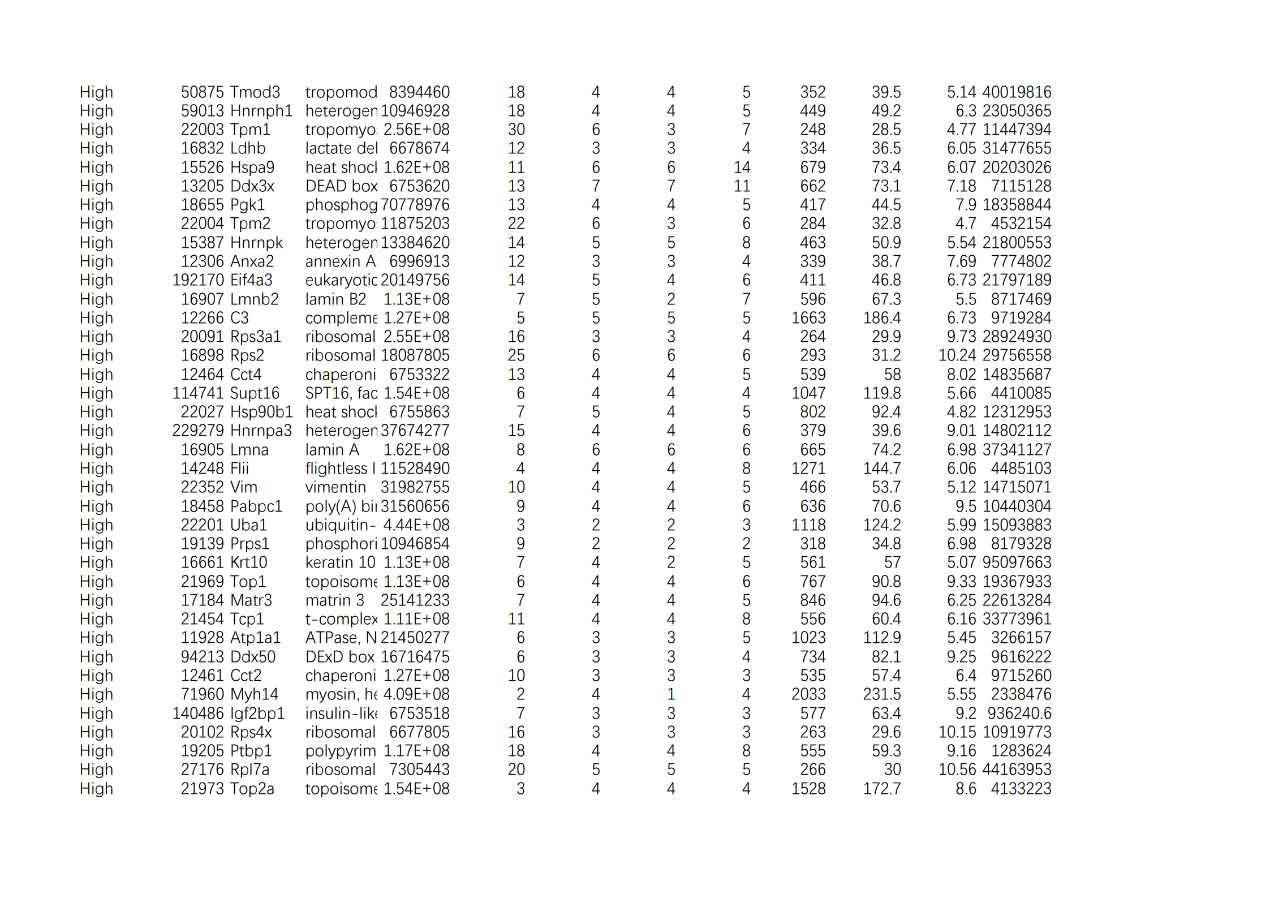
**

**
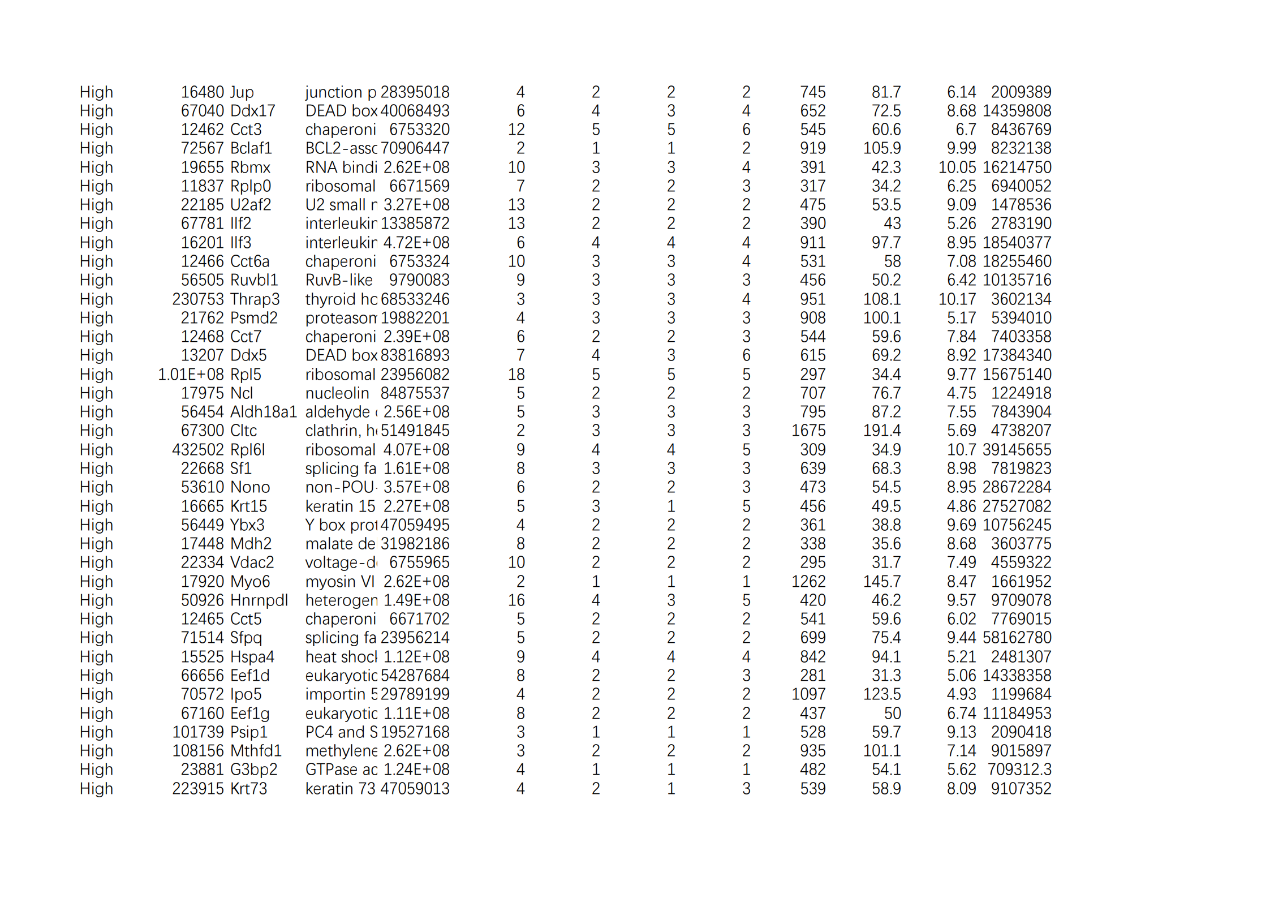
**

**
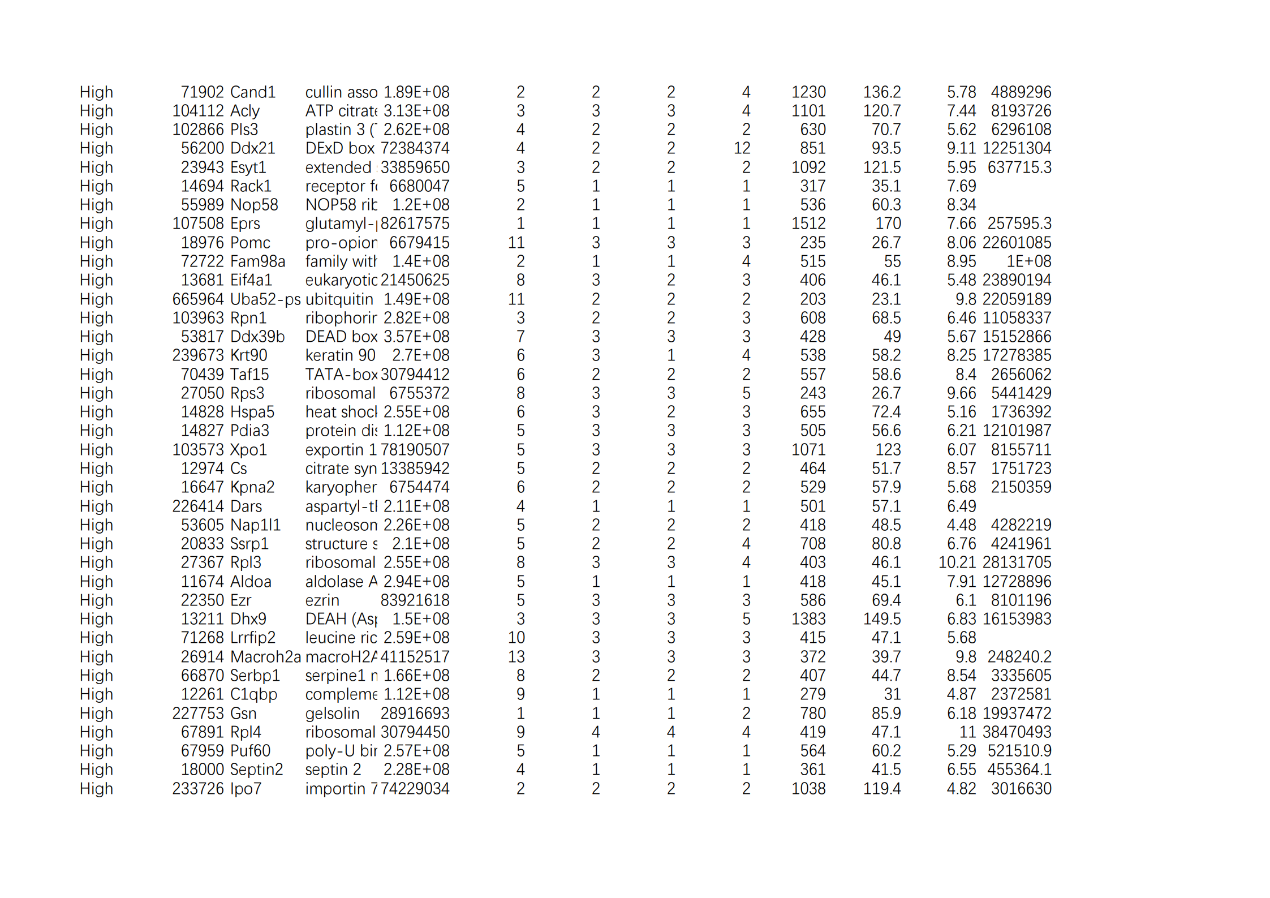
**

**
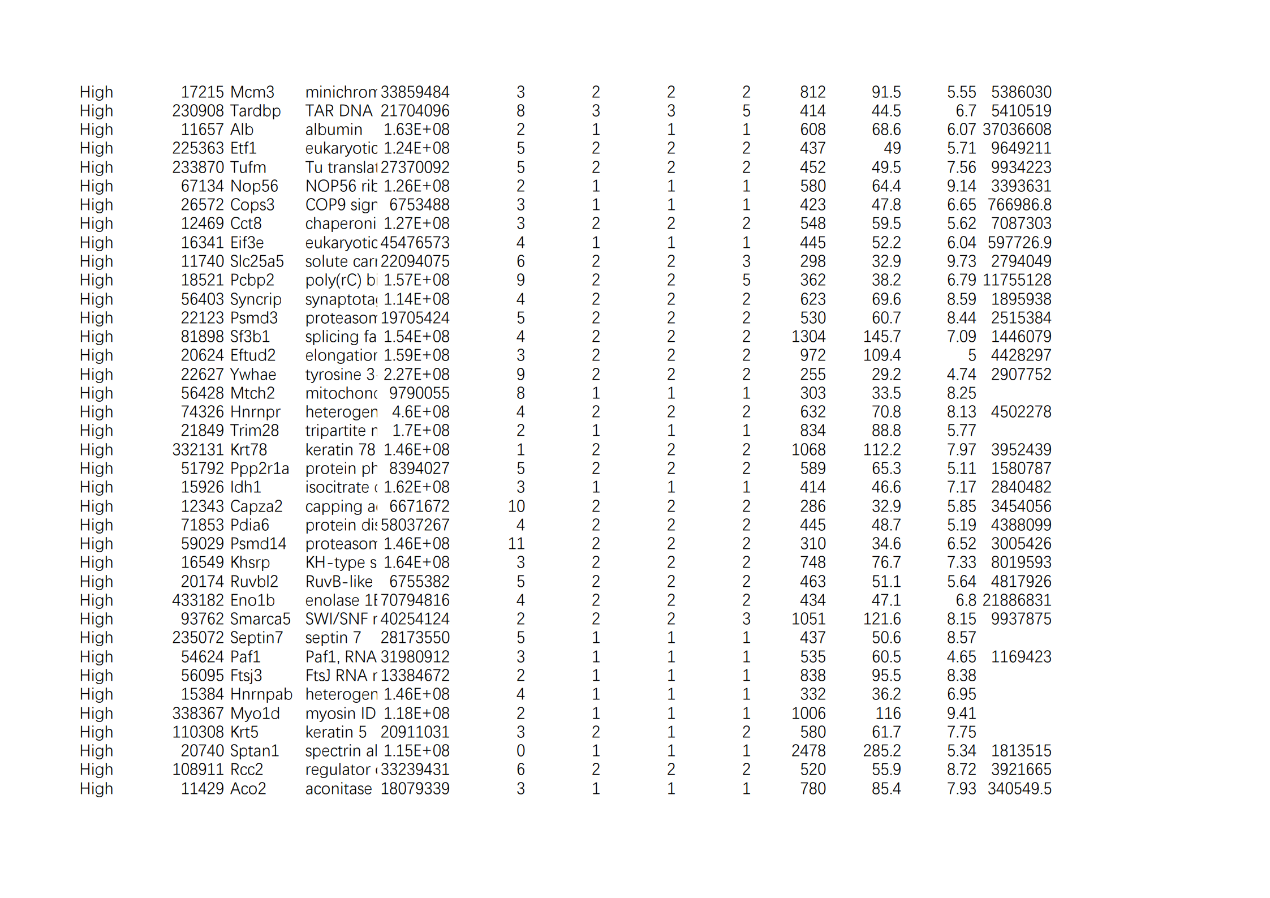
**

**
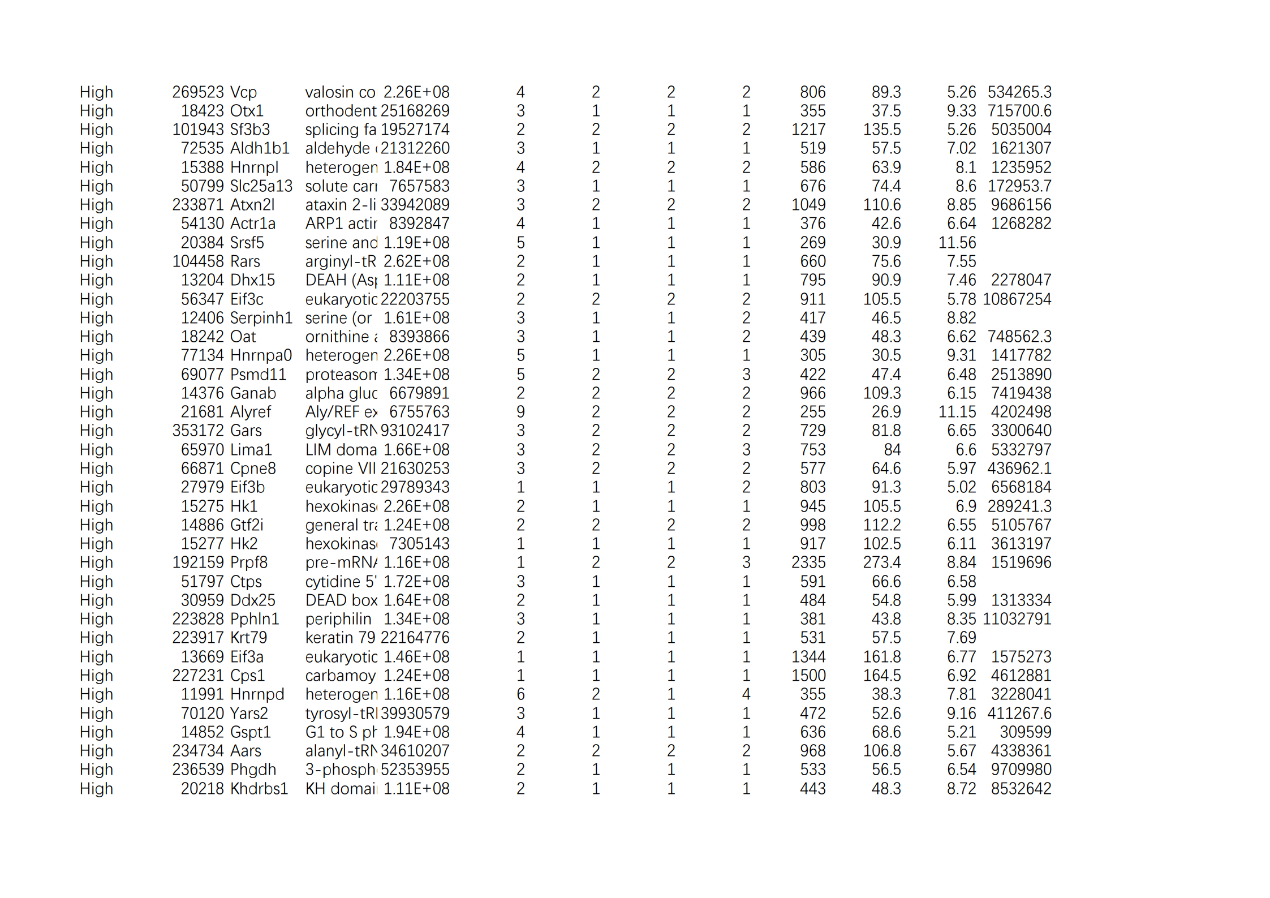
**

**
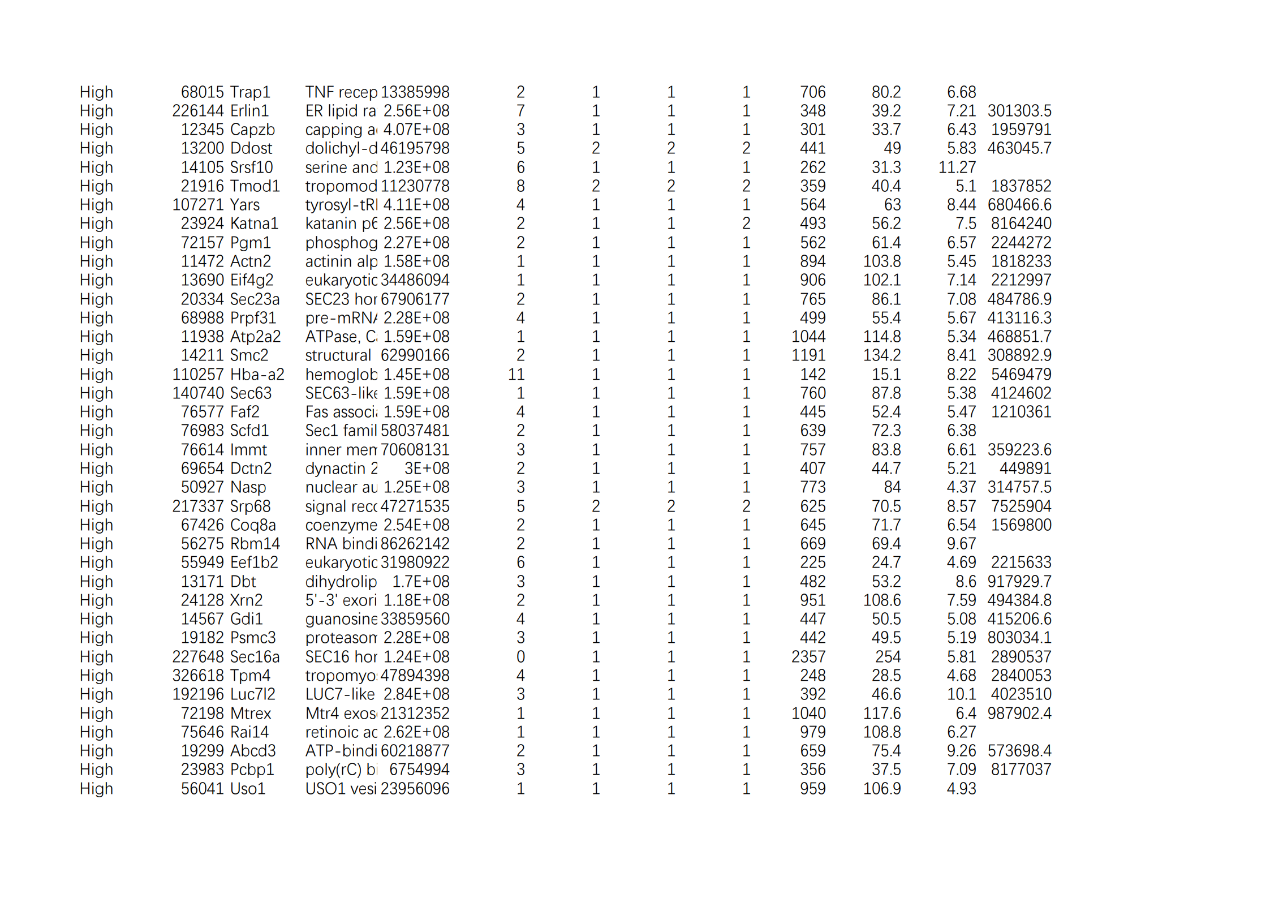
**

**
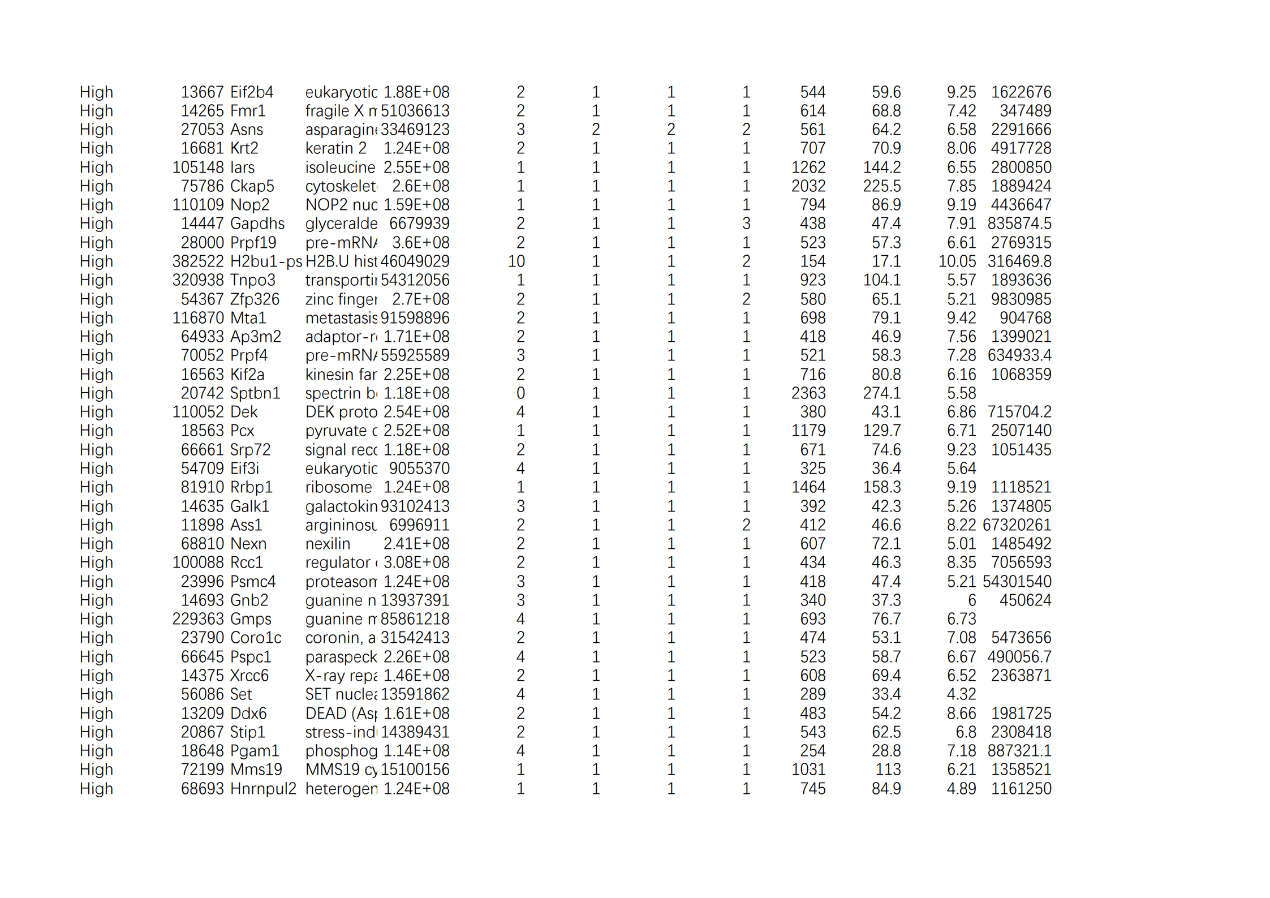
**

**
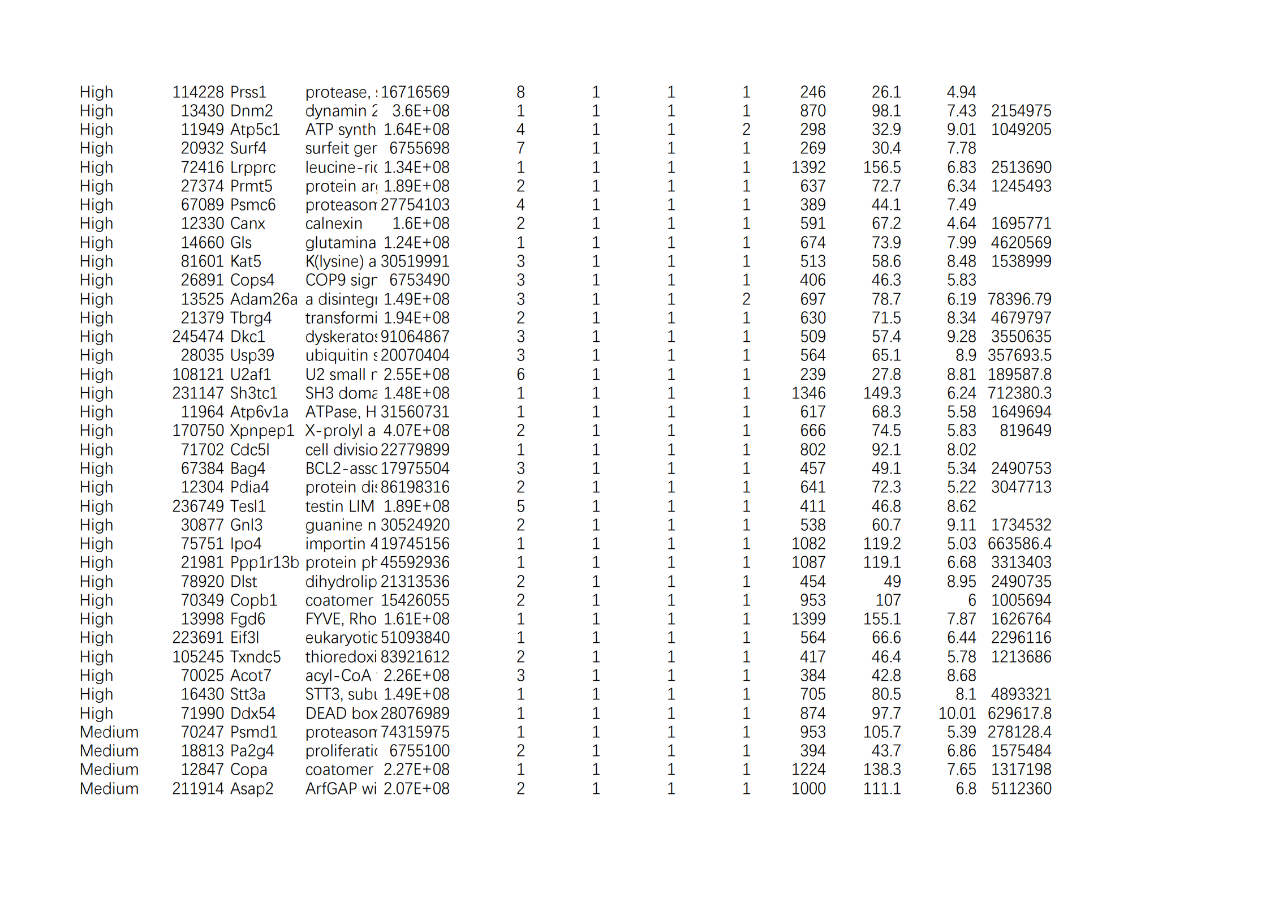
**

**
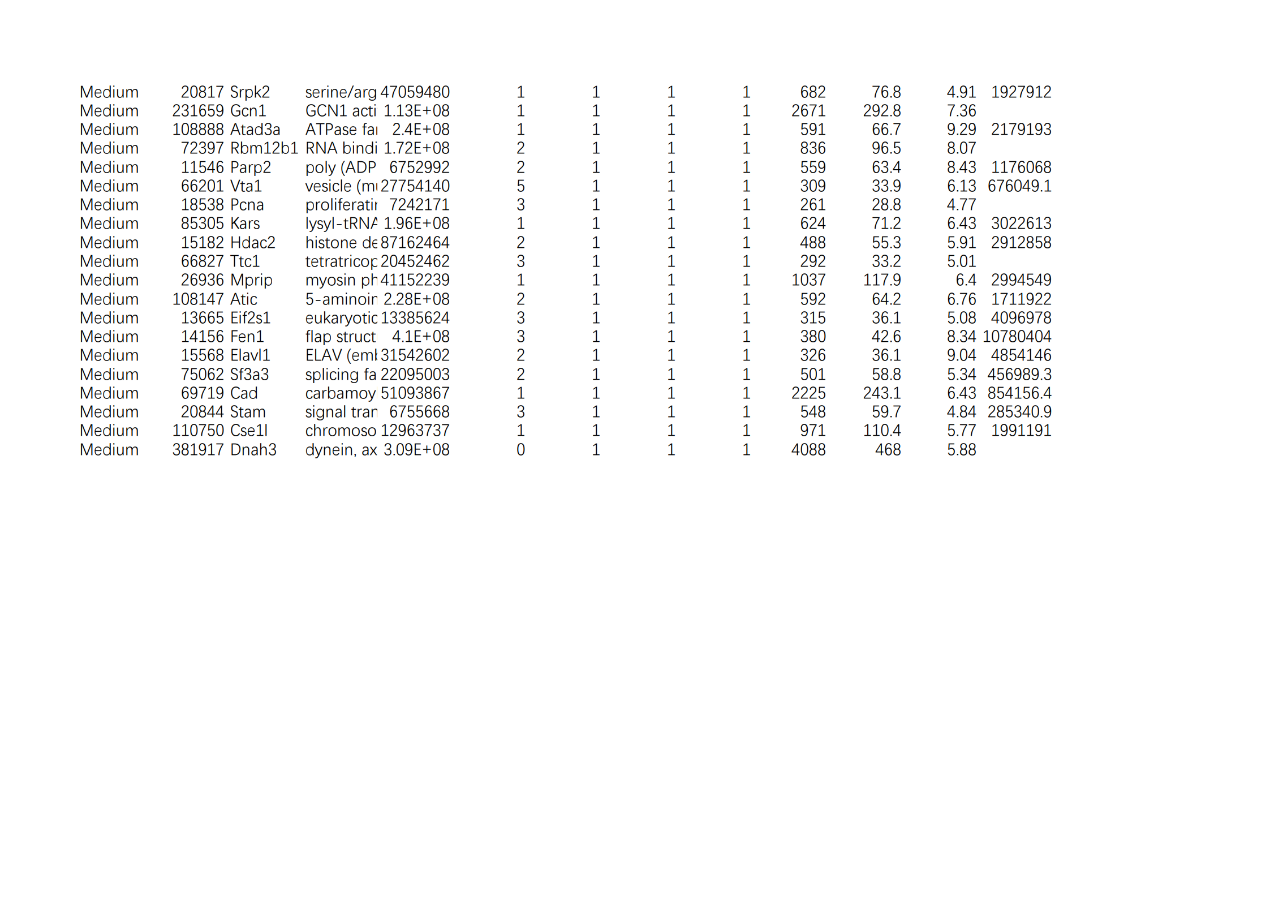
**

**Related to Fig.S6A**

**
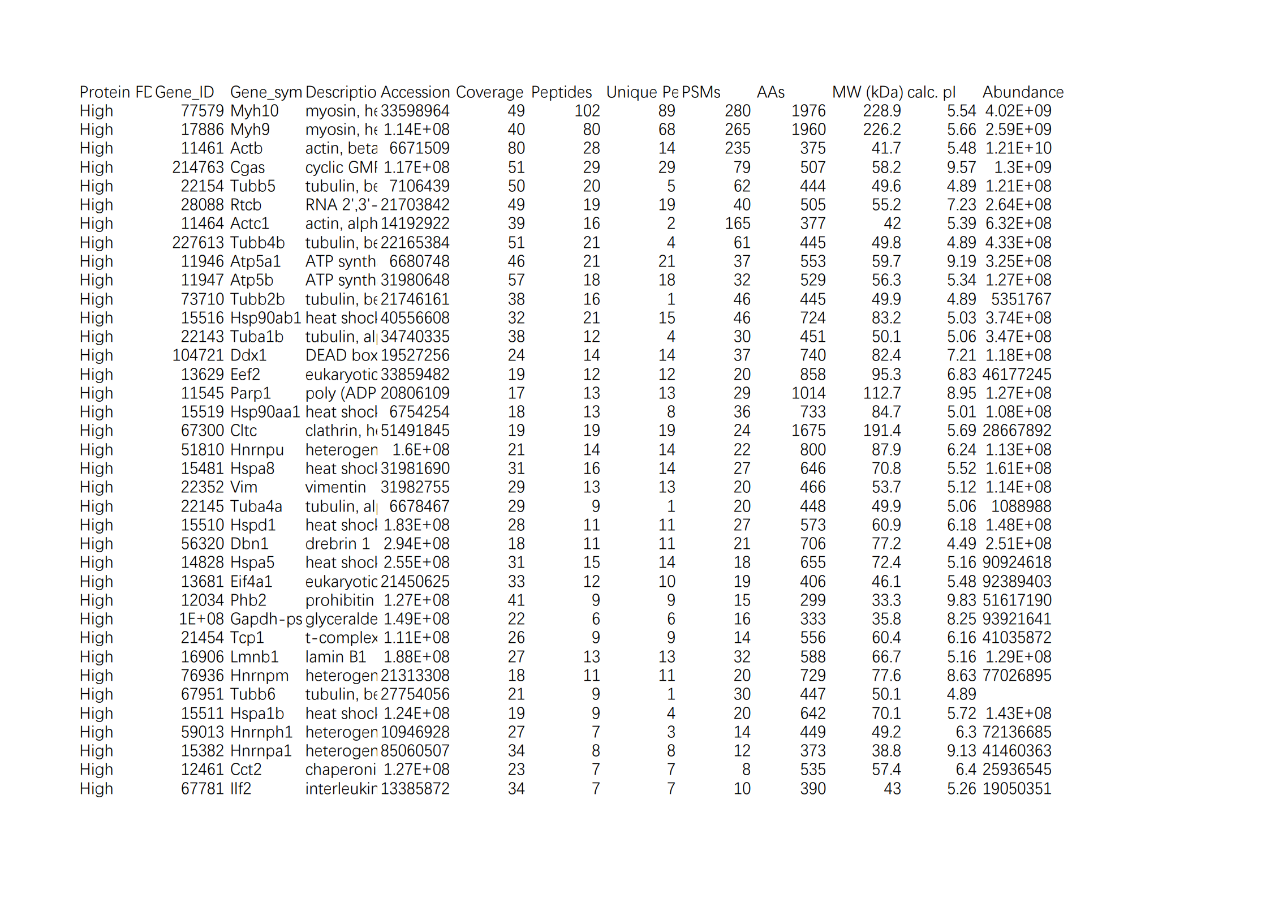
**

**
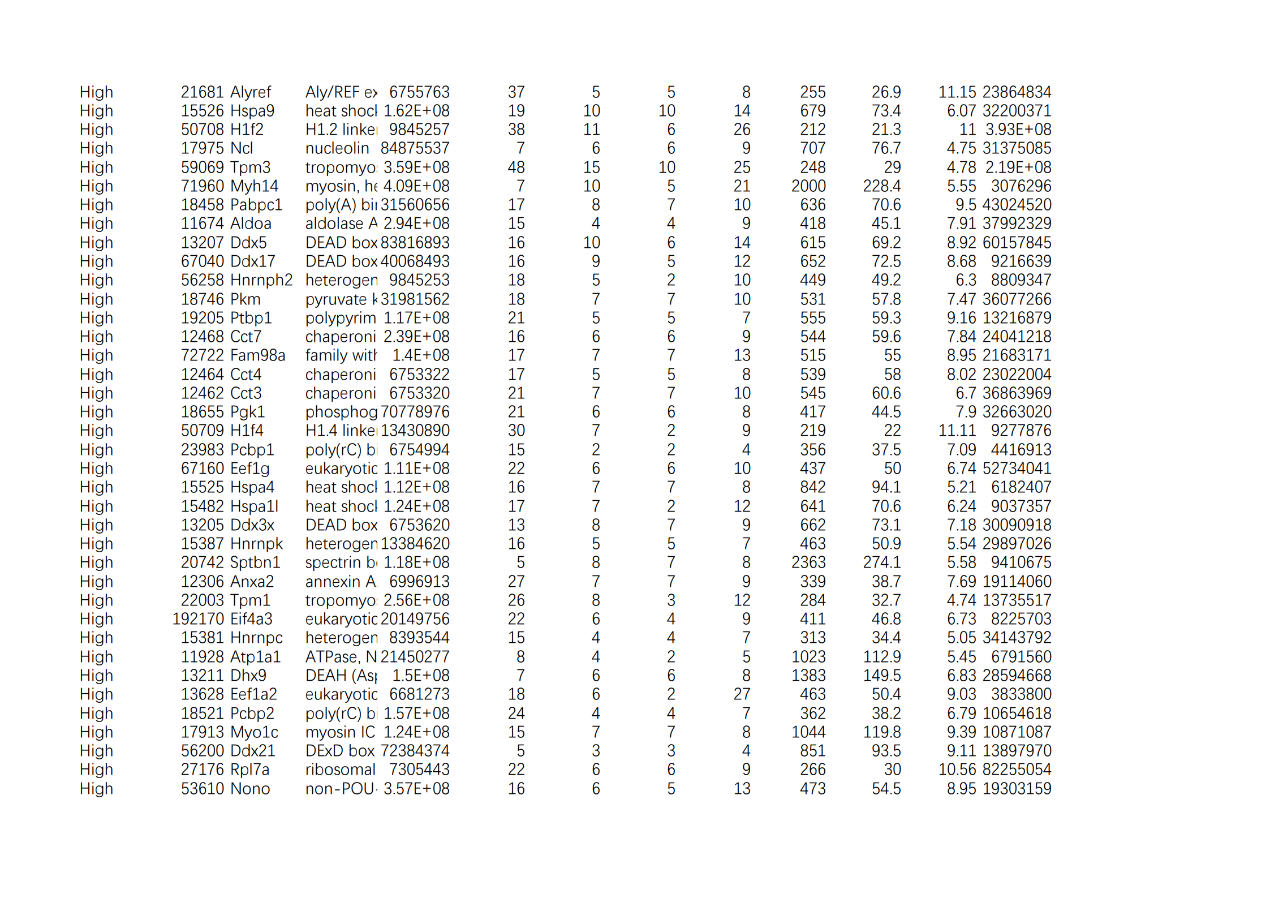
**

**
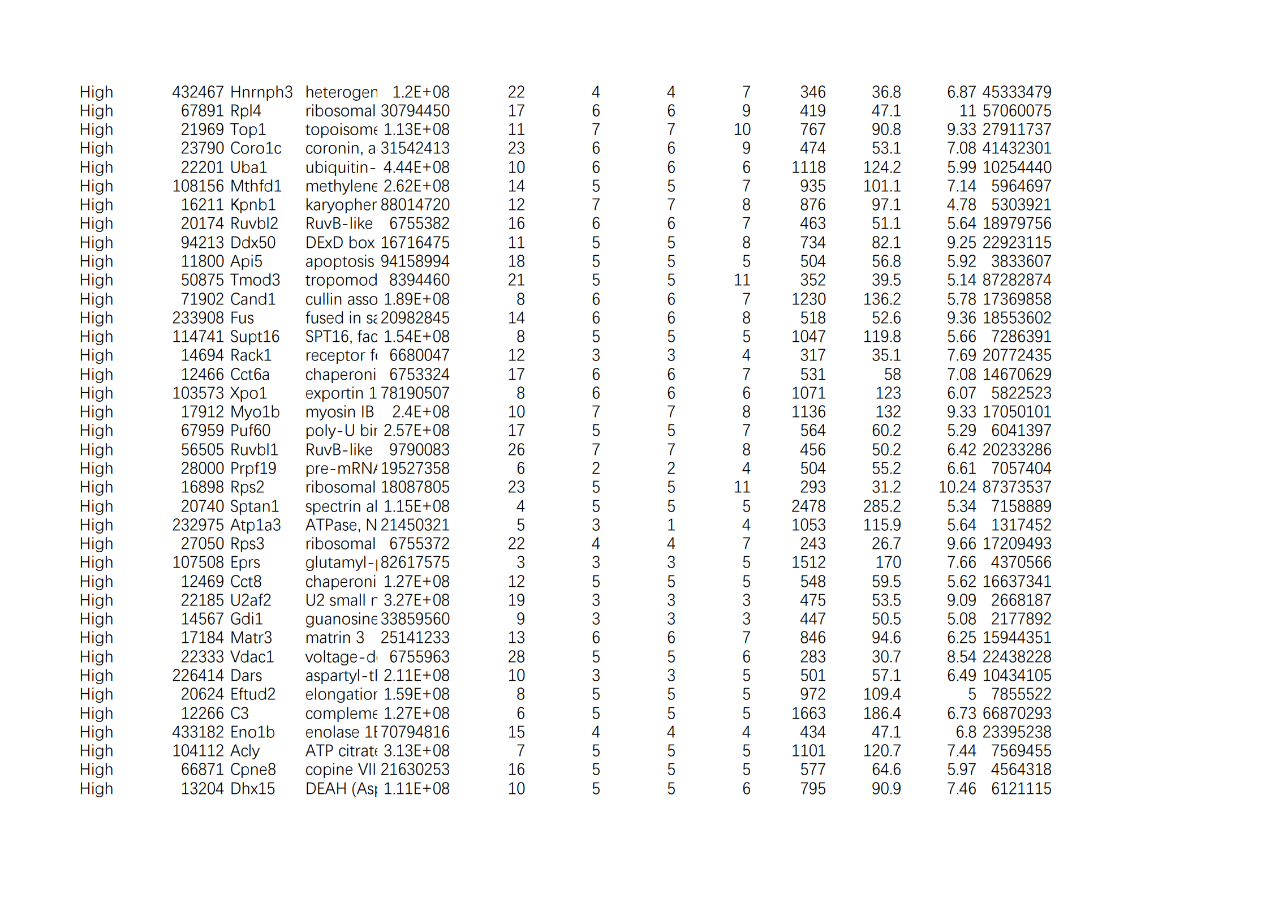
**

**
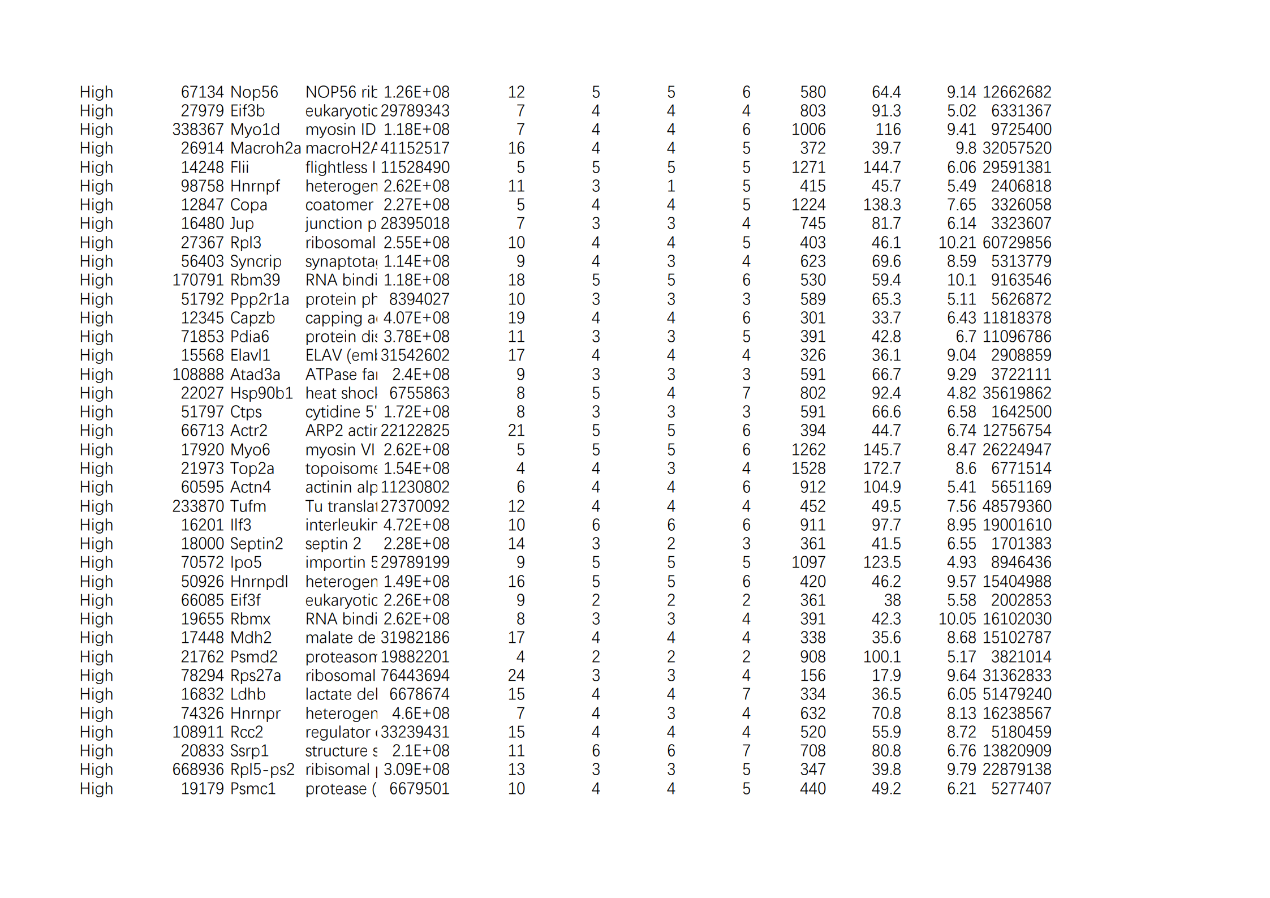
**

**
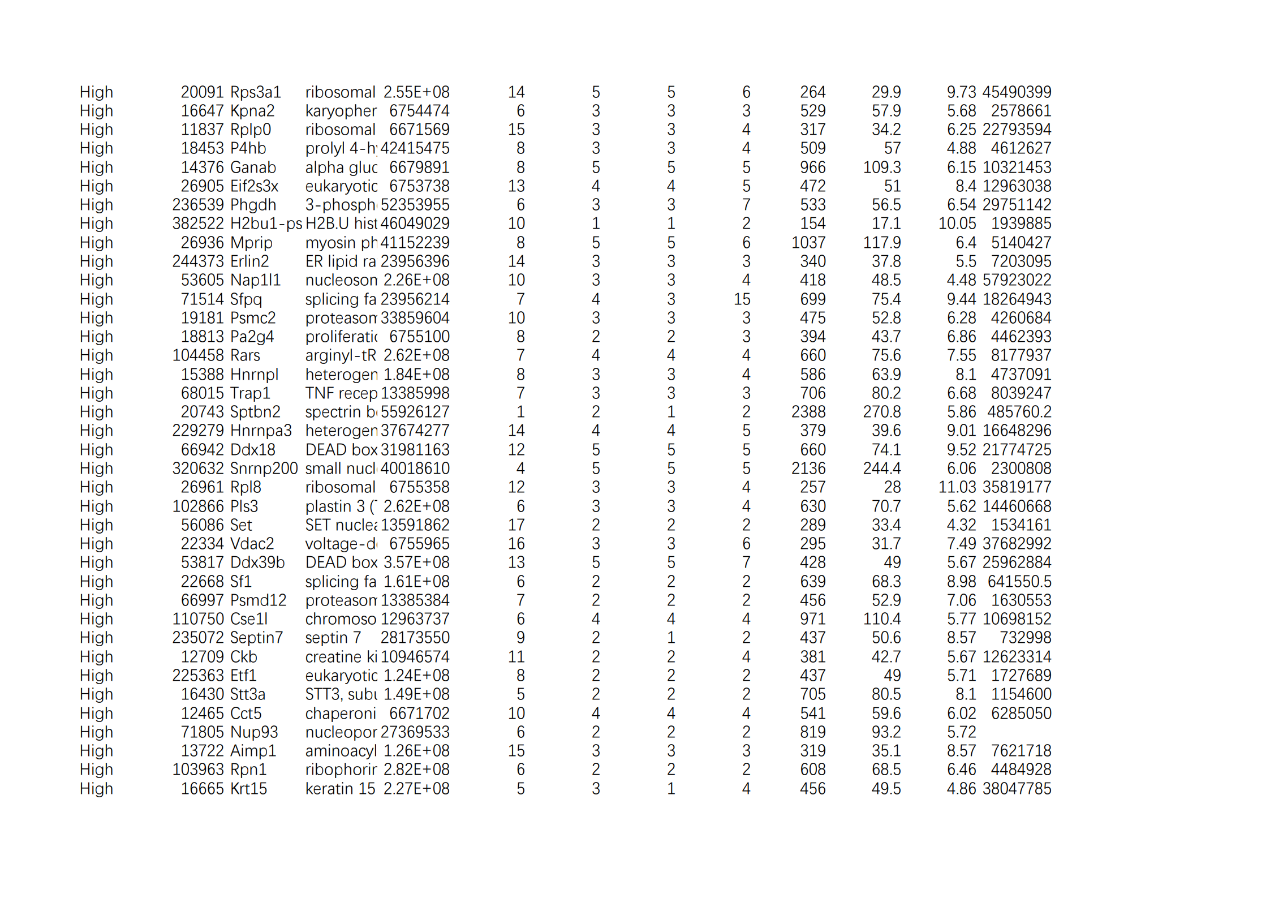
**

**
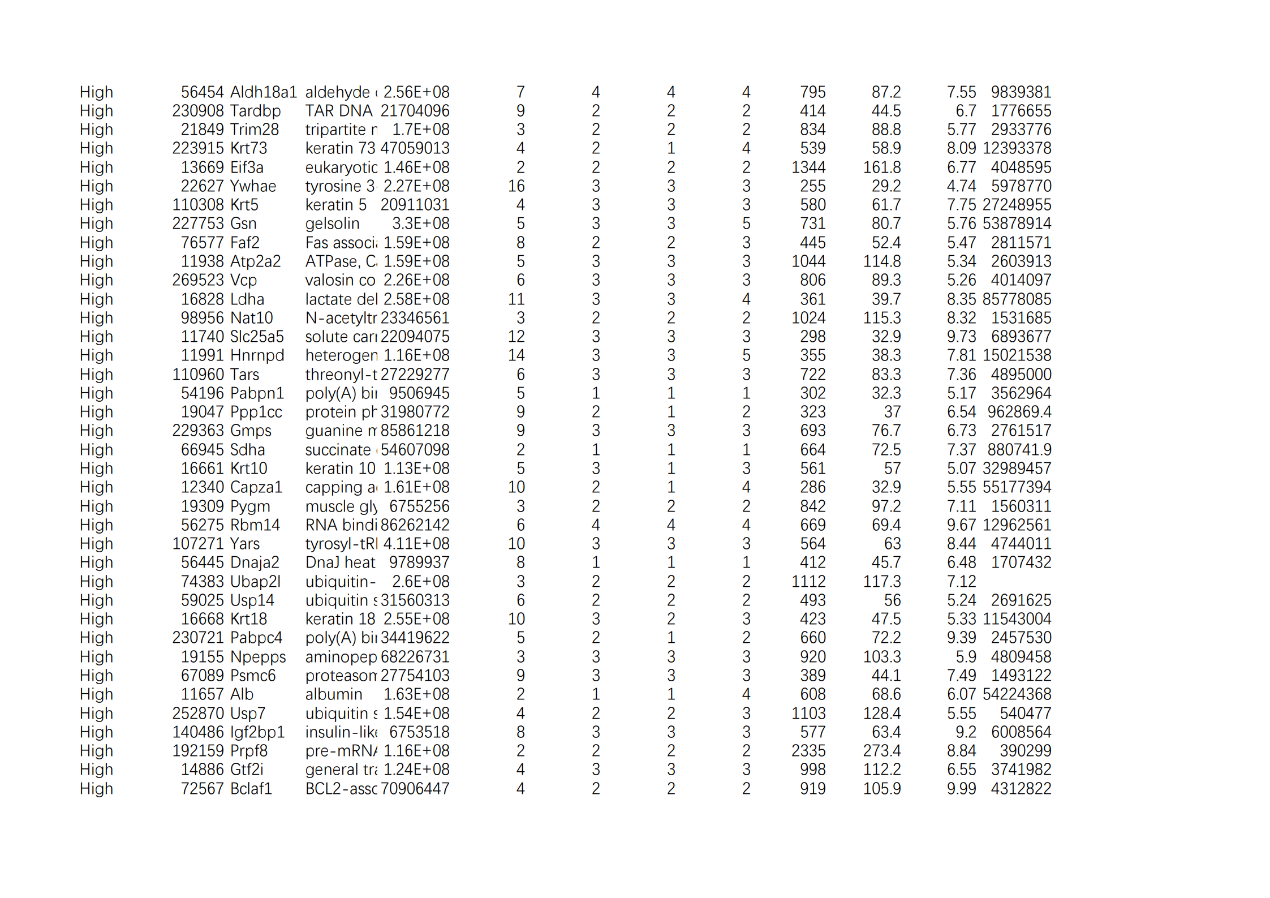
**

**
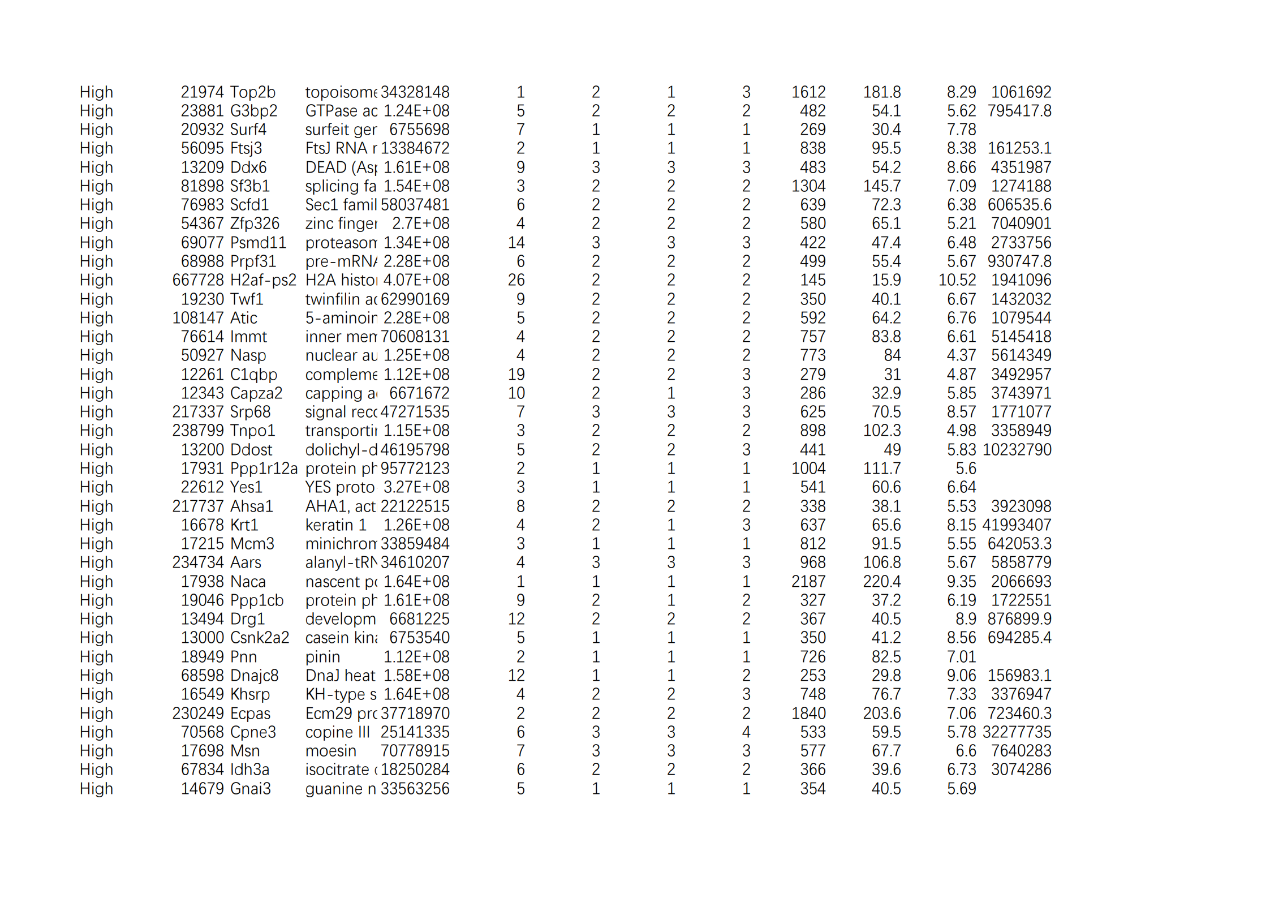
**

**
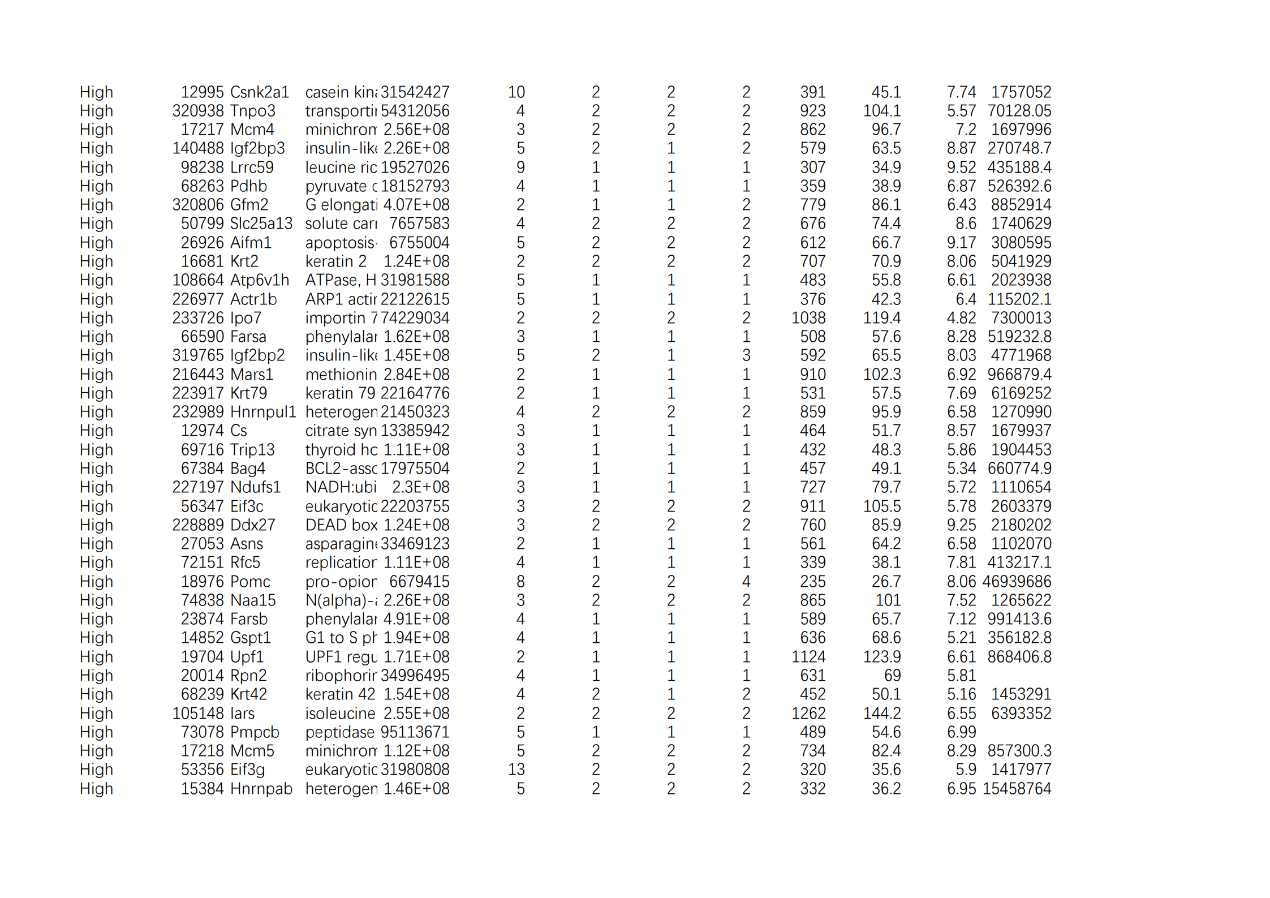
**

**
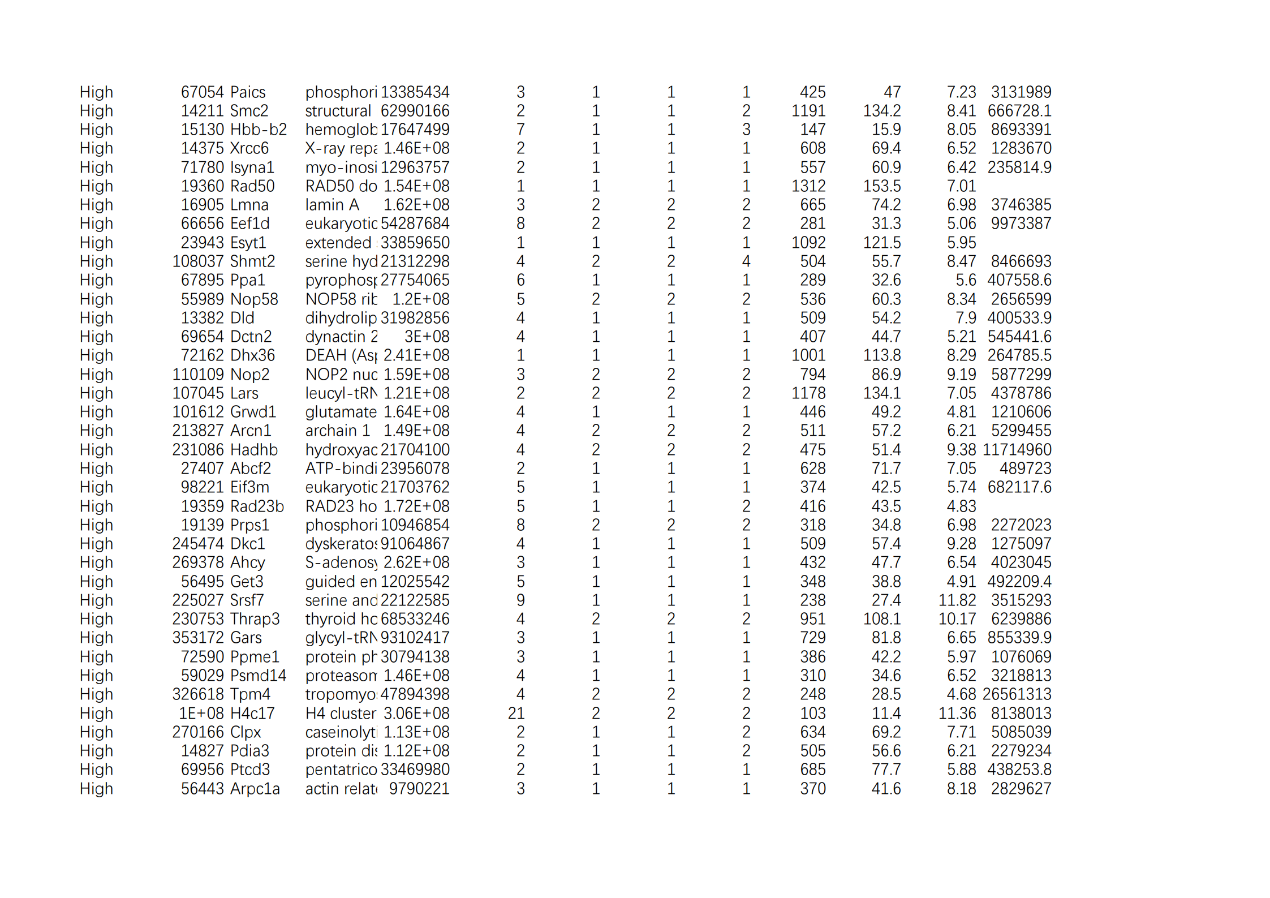
**

**
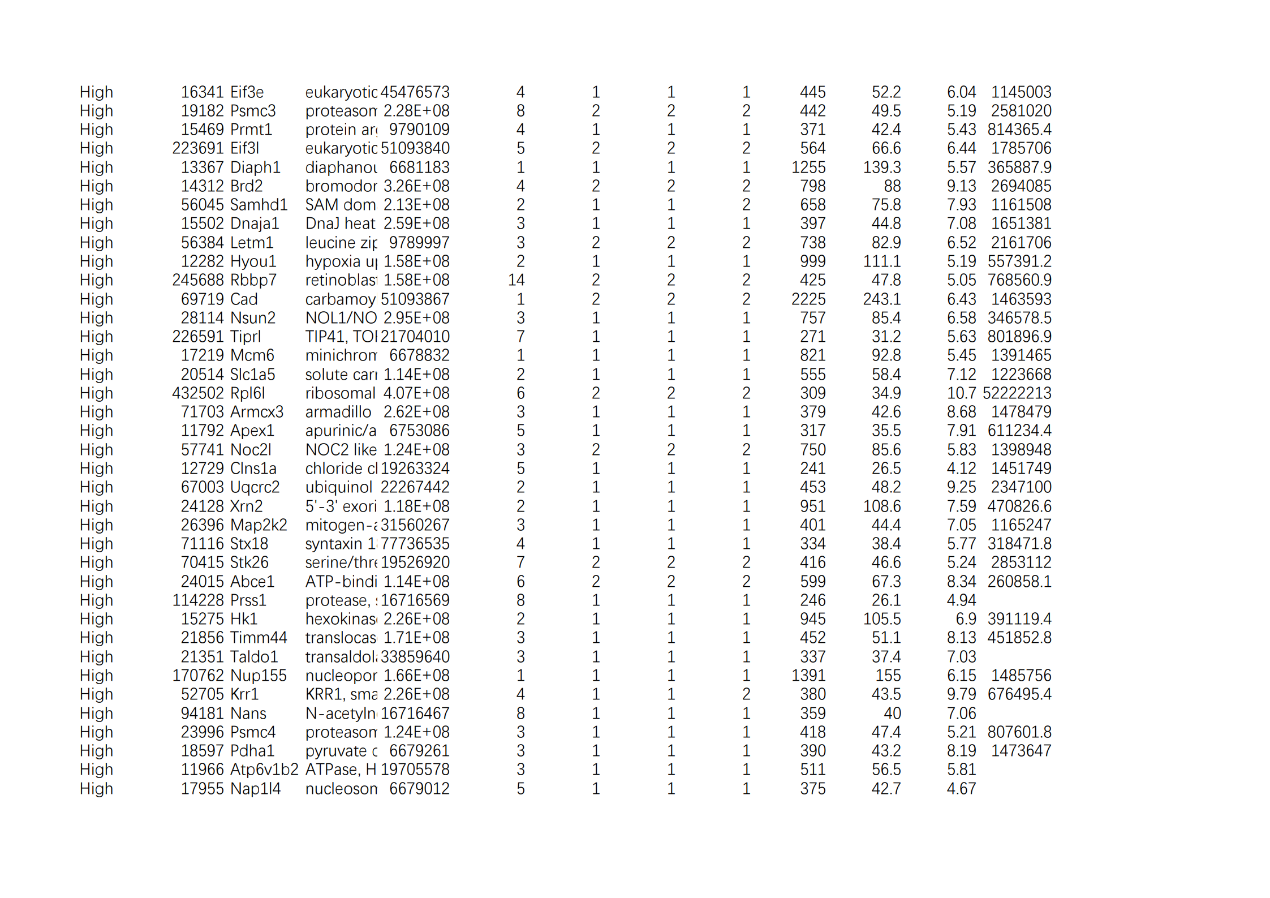
**

**
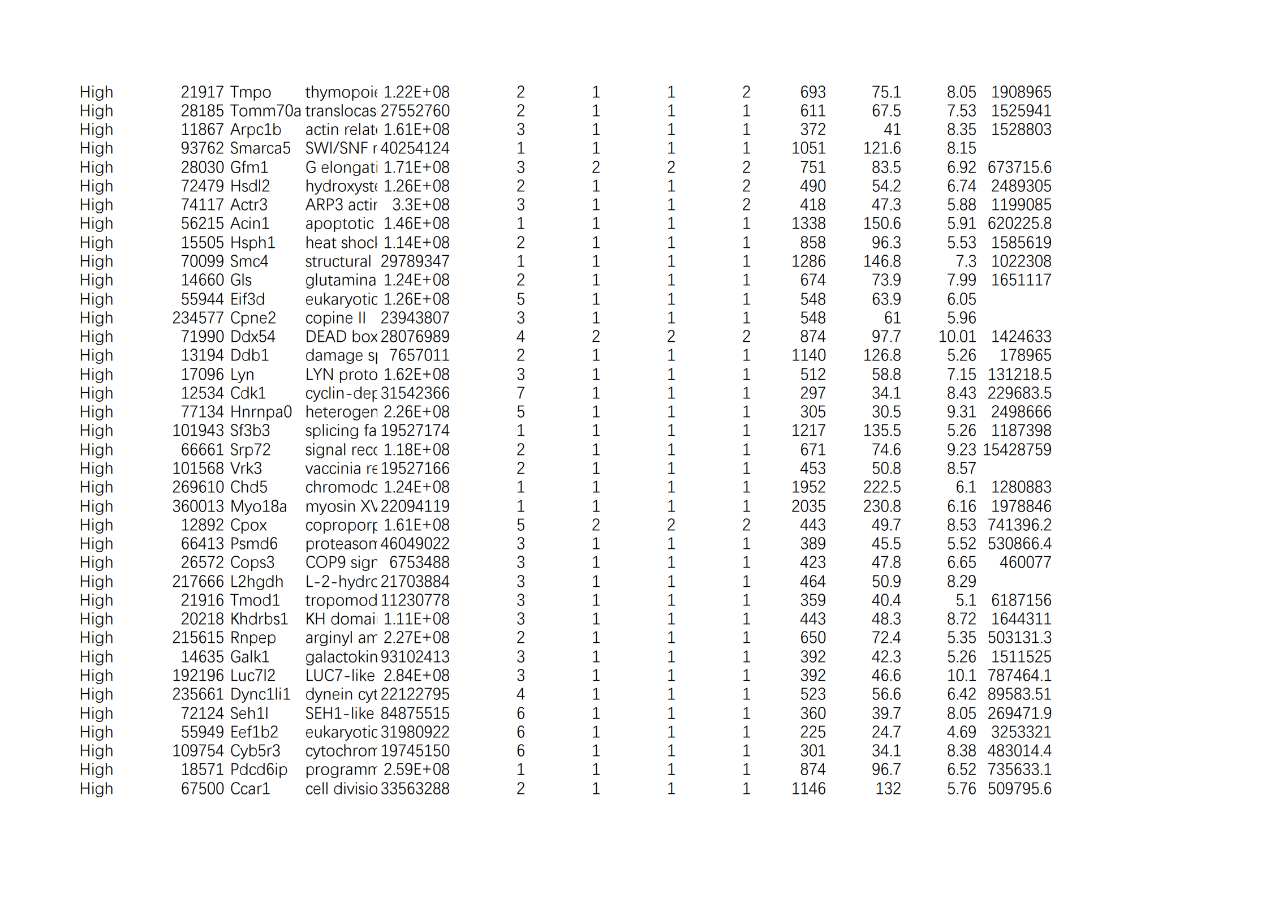
**

**
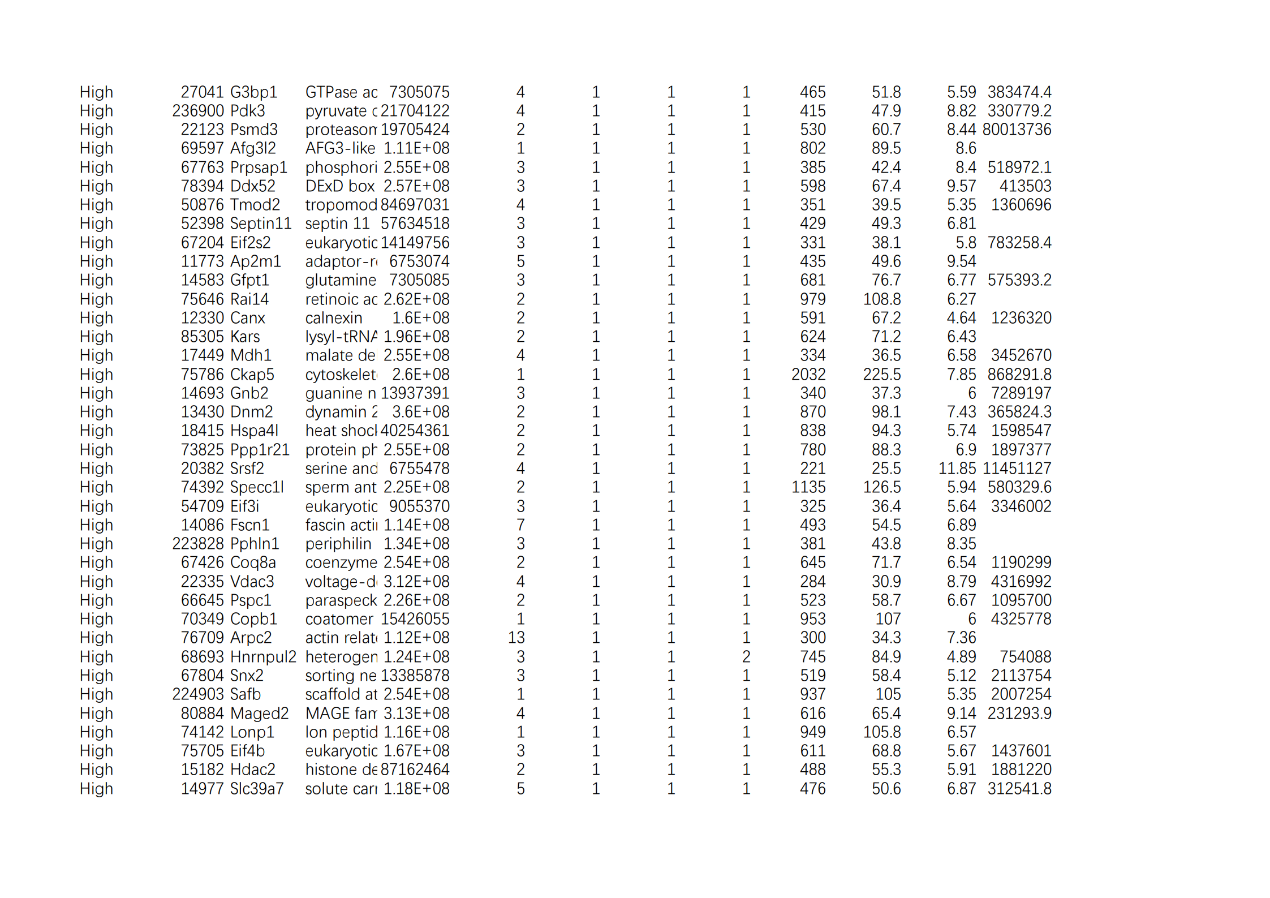
**

**
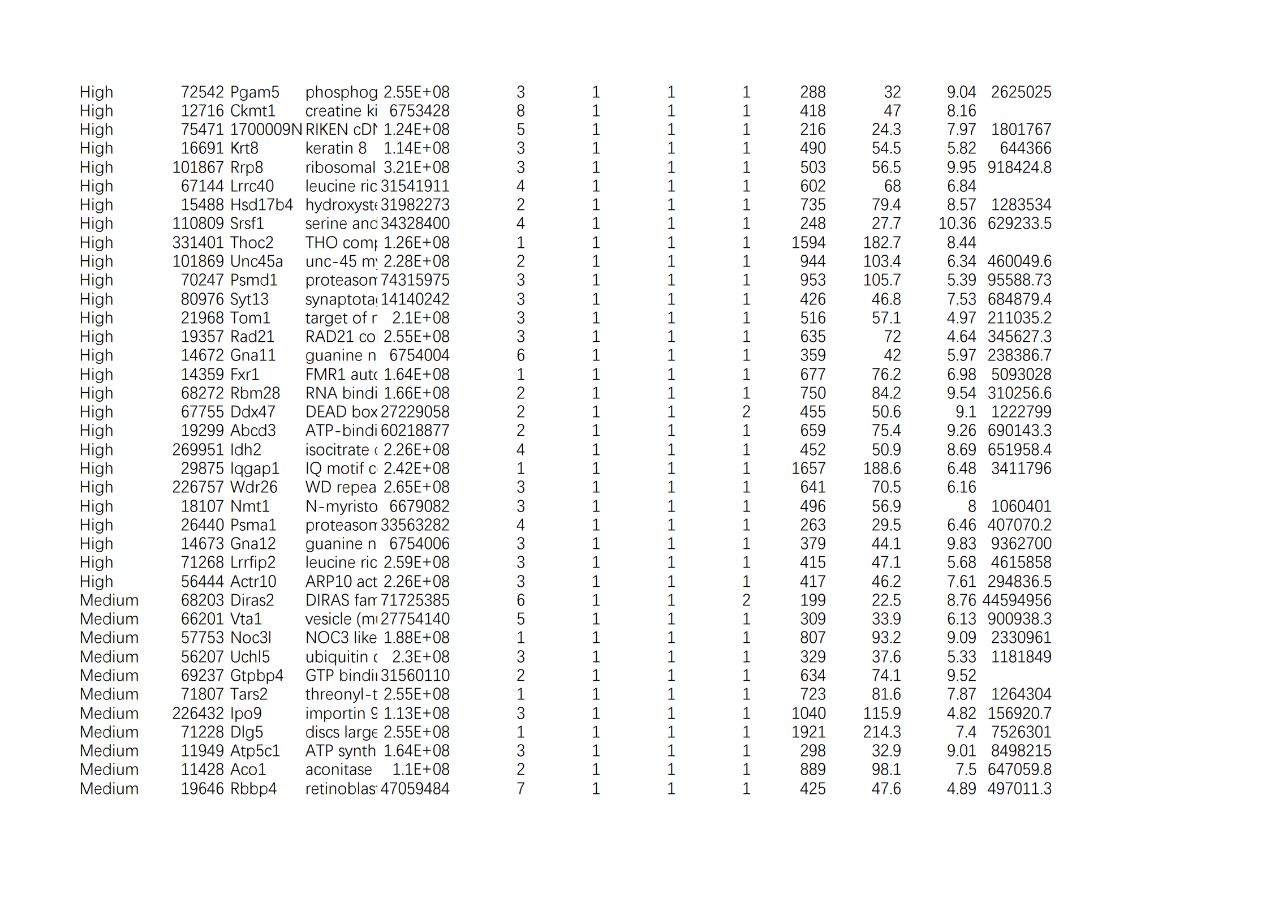
**

**
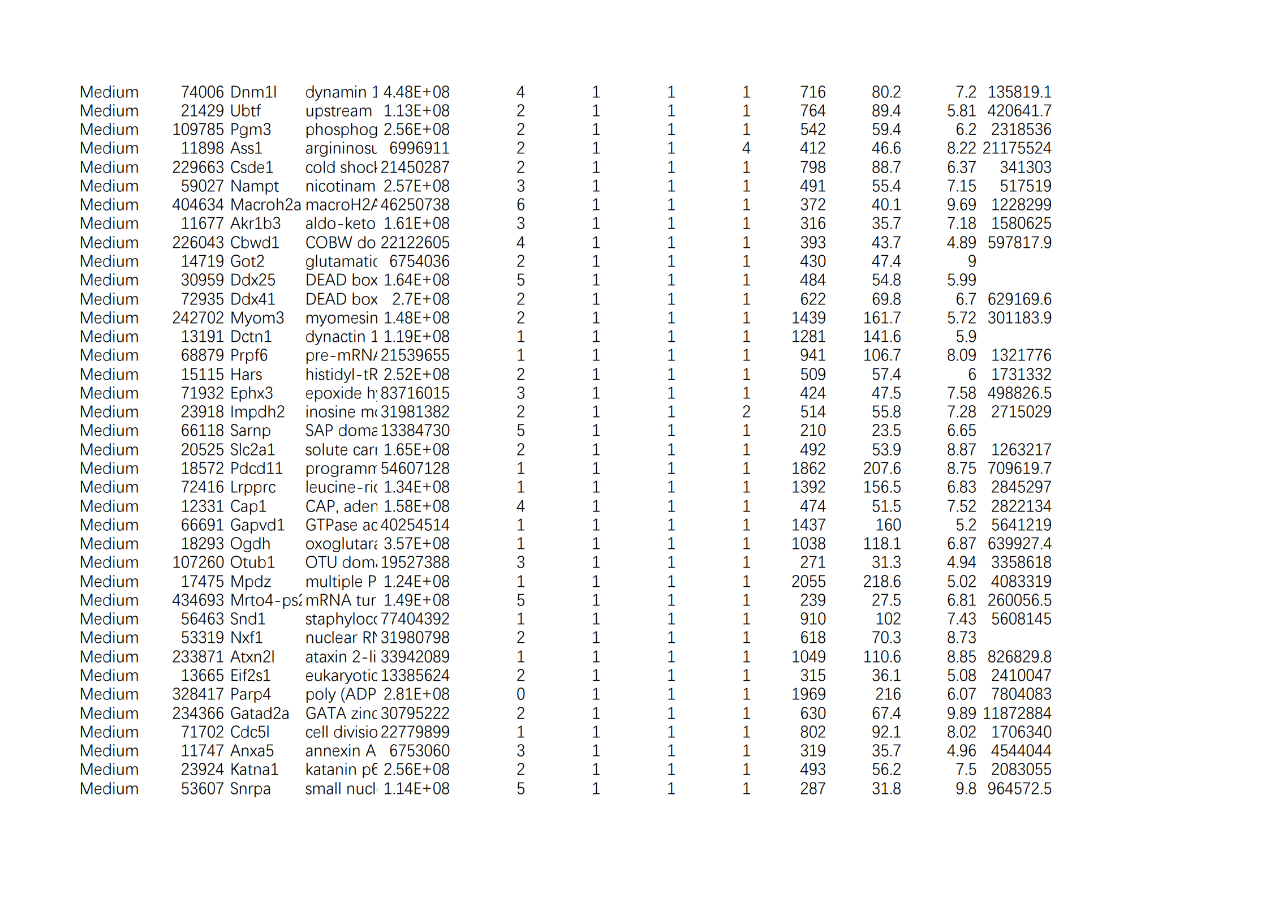
**

**
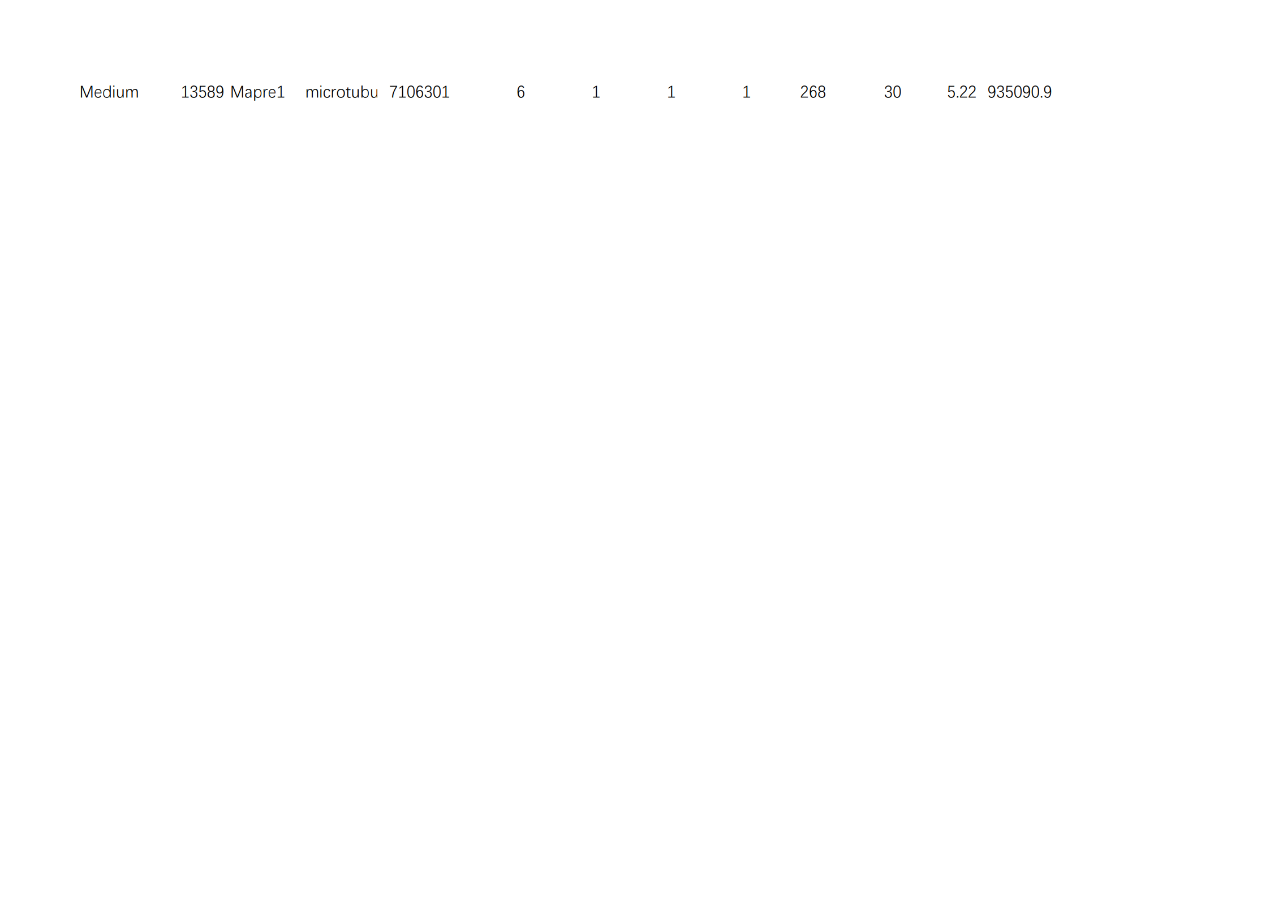
**

**Related to Fig.S6I**

**
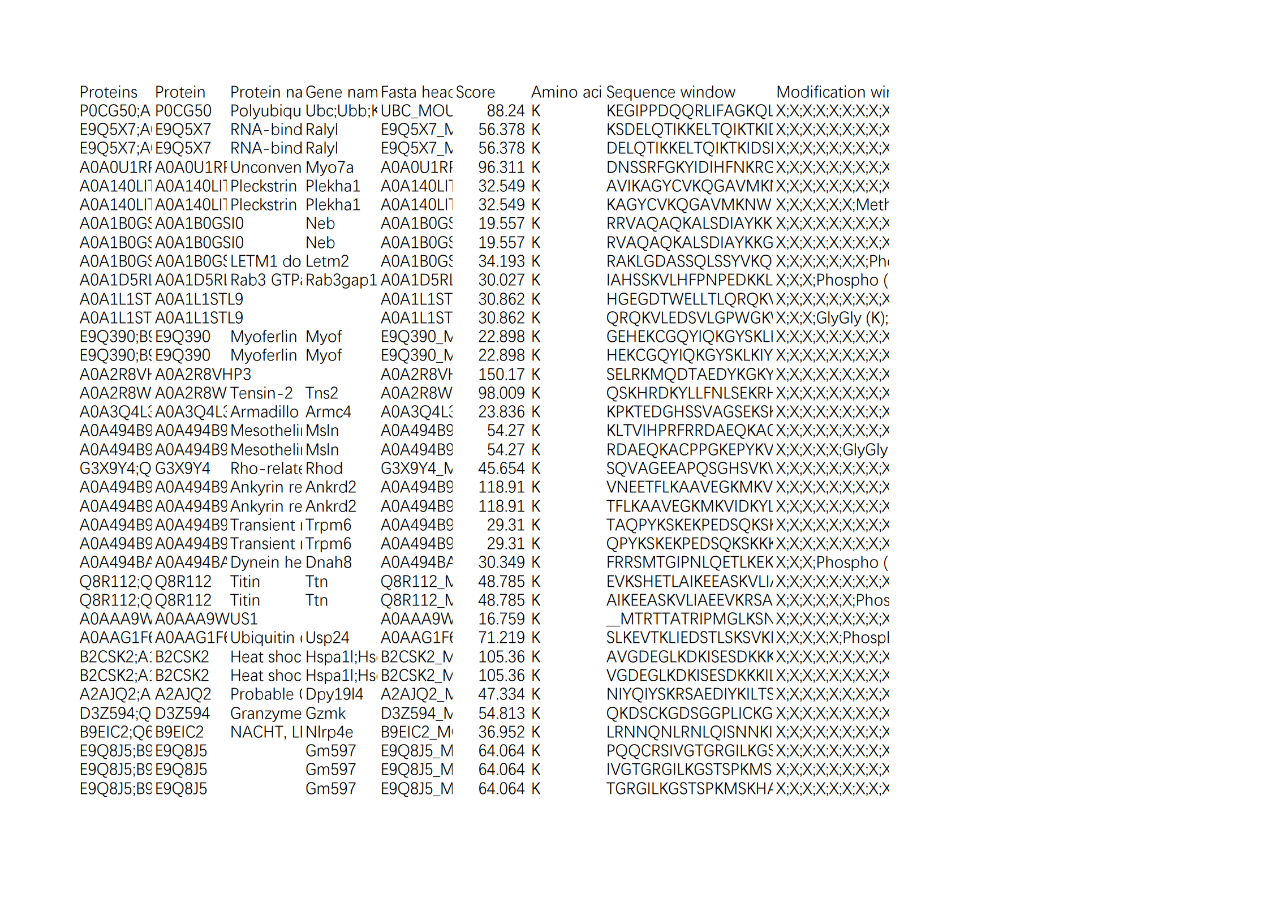
**

**
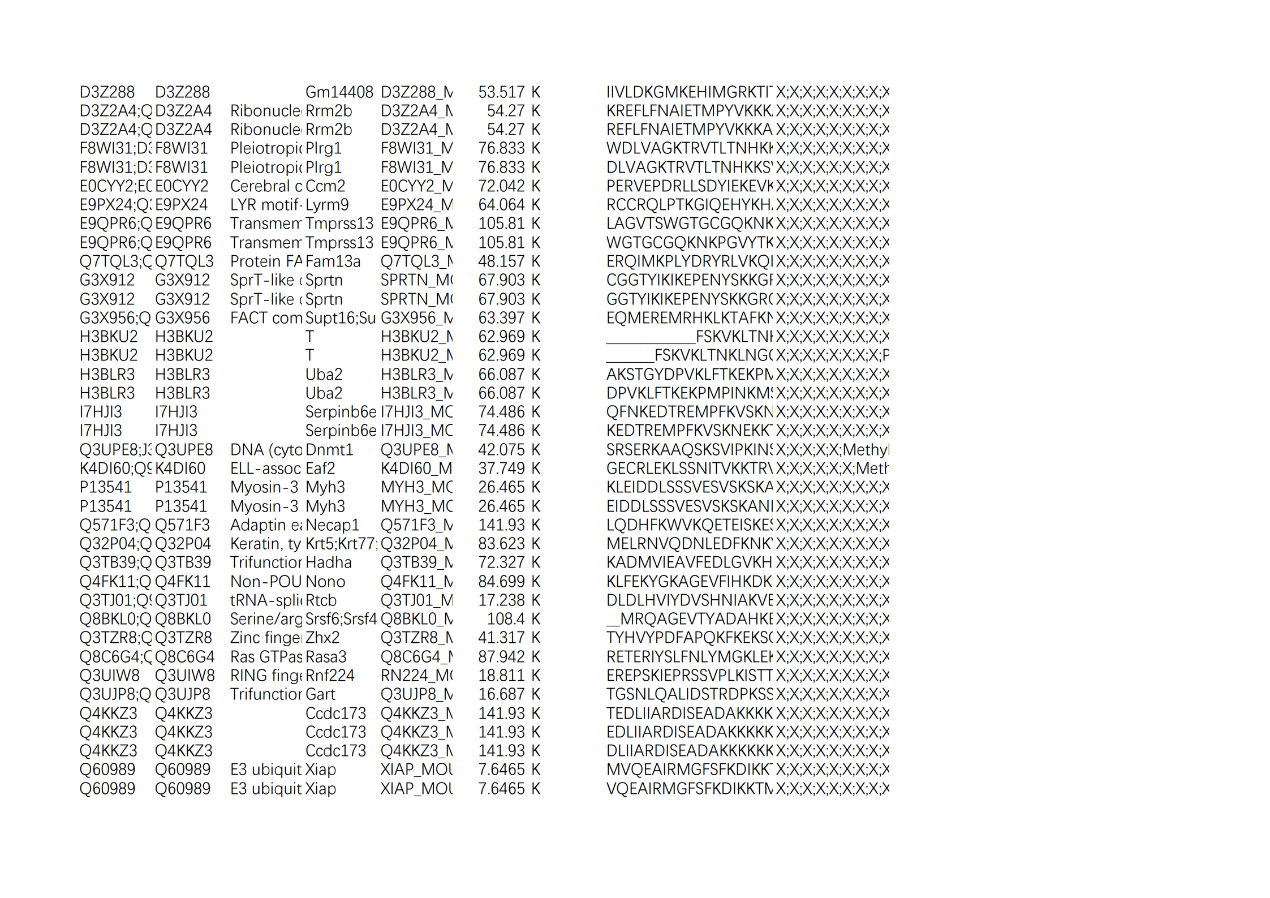
**

**
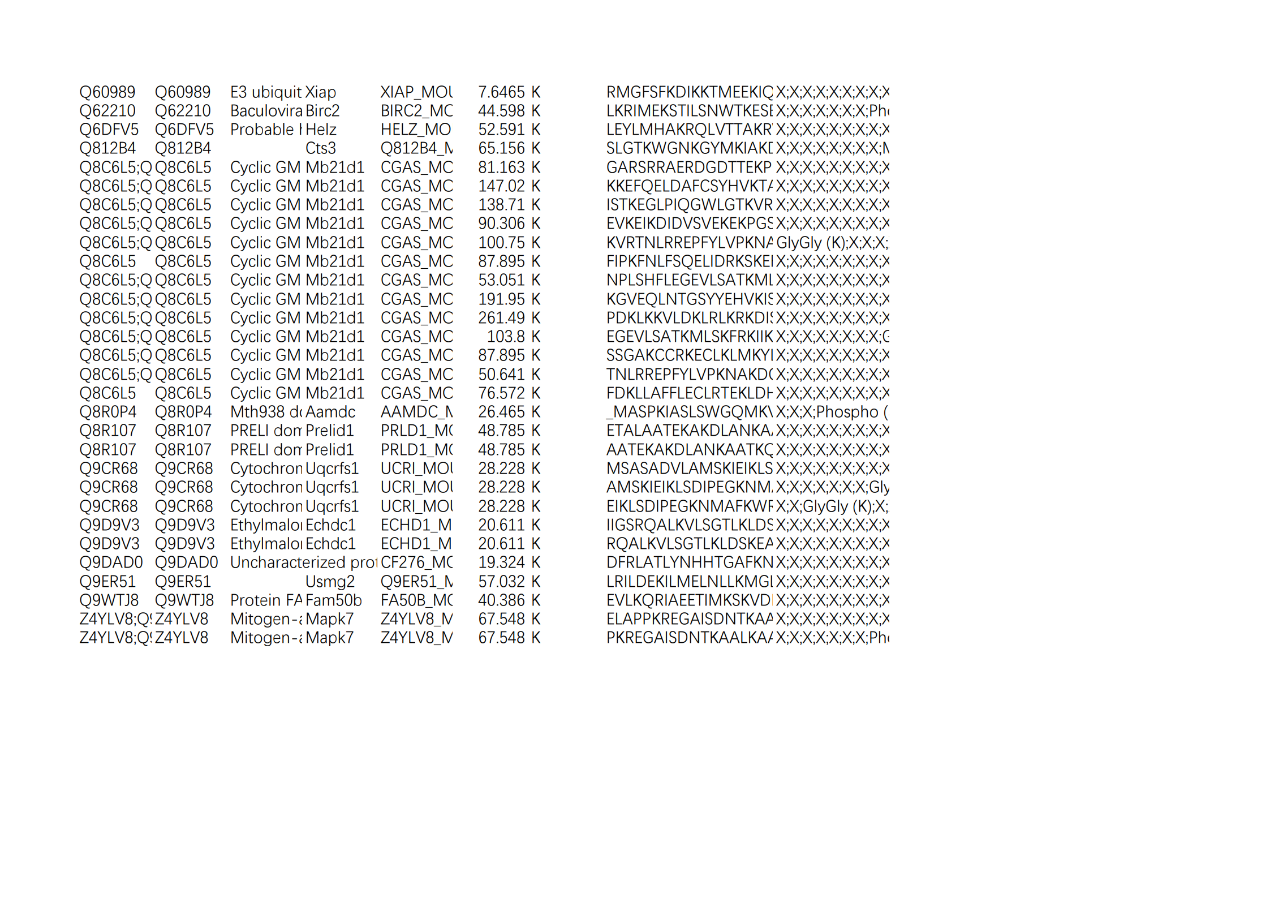
**

**
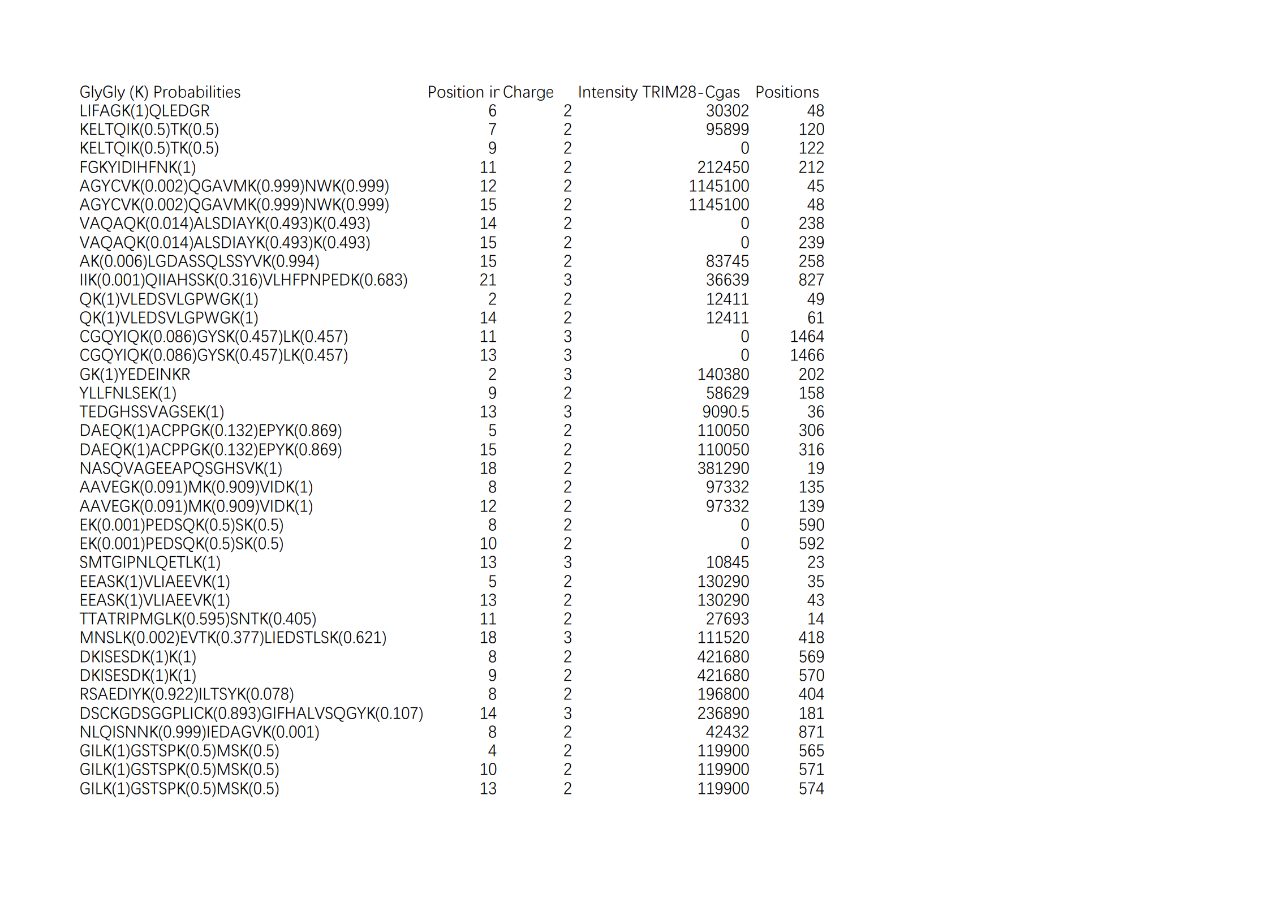
**

**
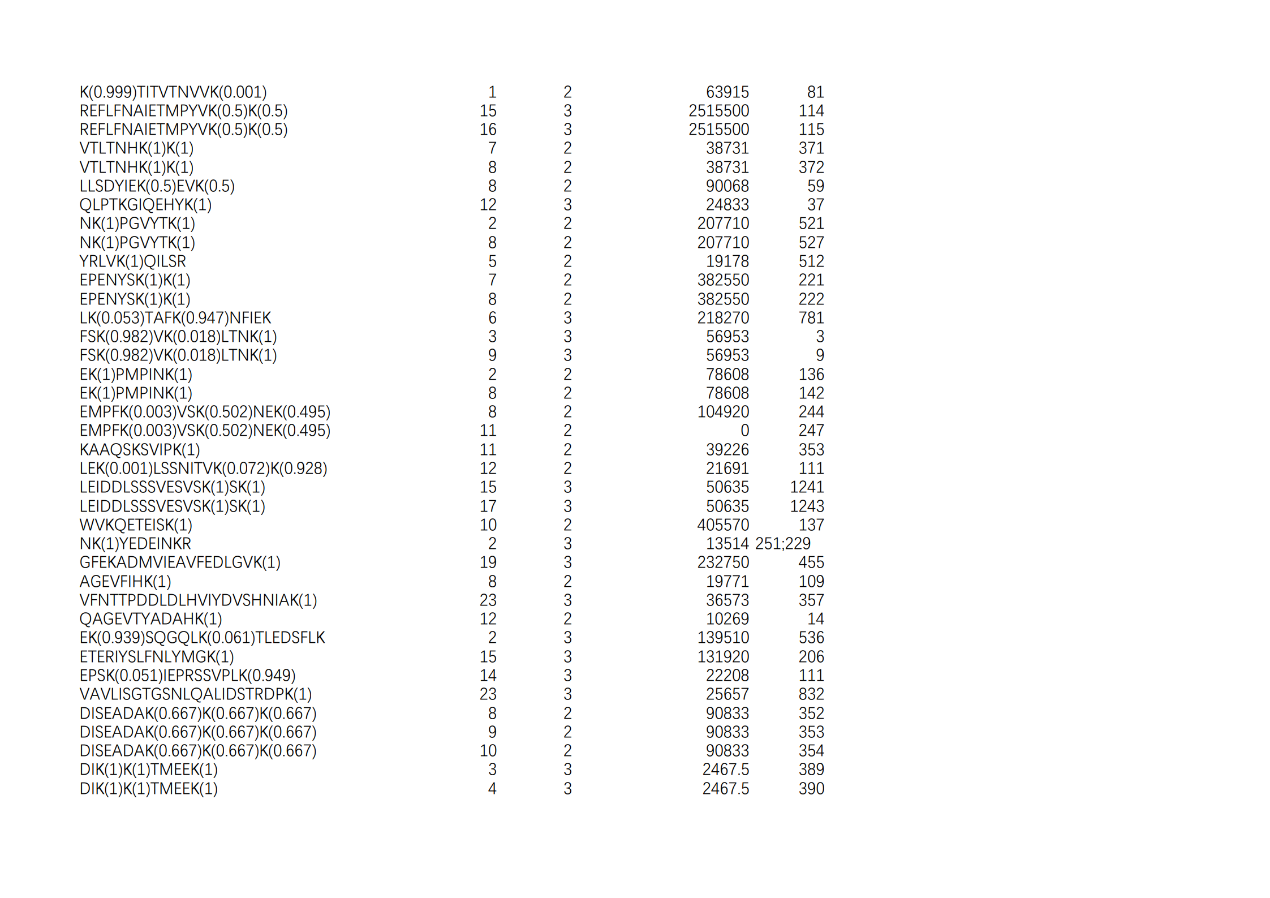
**

**
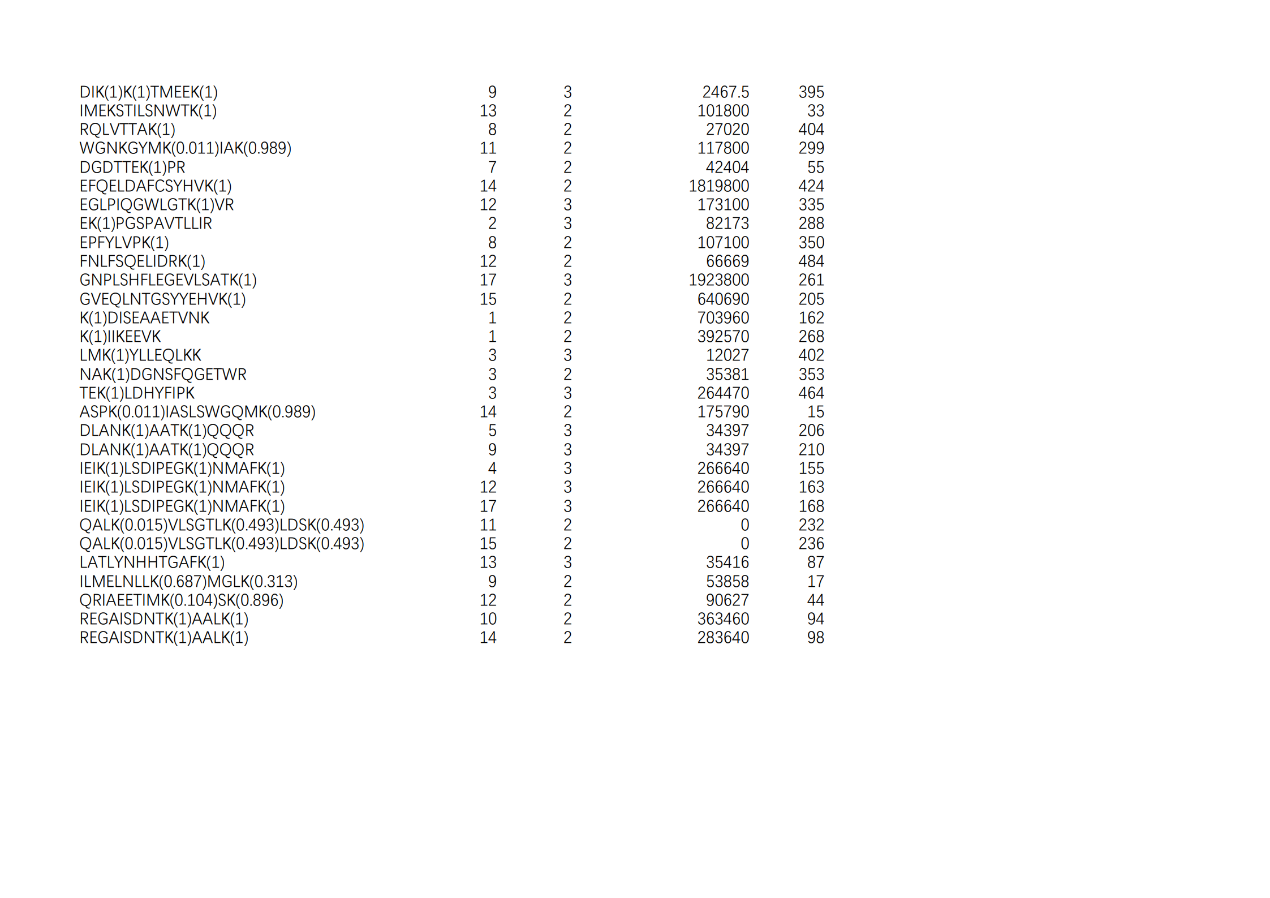
**
